# Cornification of nail keratinocytes requires autophagy for bulk degradation of intracellular proteins while sparing components of the cytoskeleton

**DOI:** 10.1007/s10495-018-1505-4

**Published:** 2018-12-14

**Authors:** Karin Jaeger, Supawadee Sukseree, Shaomin Zhong, Brett S. Phinney, Veronika Mlitz, Maria Buchberger, Marie Sophie Narzt, Florian Gruber, Erwin Tschachler, Robert H. Rice, Leopold Eckhart

**Affiliations:** 1 Research Division of Biology and Pathobiology of the Skin, Department of Dermatology, Medical University of Vienna, Lazarettgasse 14, 1090 Vienna, Austria; 2 Proteomics Core Facility, UC Davis Genome Center, University of California, Davis, CA USA; 3 Christian Doppler Laboratory on Biotechnology of Skin Aging, Vienna, Austria; 4 Department of Environmental Toxicology, University of California, One Shields Avenue, Davis, CA 95616-8588 USA

**Keywords:** Cornification, Autophagy, Proteomics, Keratinocytes, Keratin, Nail

## Abstract

Epidermal keratinocytes undergo cornification to form the cellular building blocks of hard skin appendages such as nails and the protective layer on the surface of the skin. Cornification requires the cross-linking of structural proteins and the removal of other cellular components to form mechanically rigid and inert corneocytes. Autophagy has been proposed to contribute to this intracellular remodelling process, but its molecular targets in keratinocytes, if any, have remained elusive. Here, we deleted the essential autophagy factor Atg7 in K14-positive epithelia of mice and determined by proteomics the impact of this deletion on the abundance of individual proteins in cornified nails. The genetic suppression of autophagy in keratinocytes resulted in a significant increase in the number of proteins that survived cornification and in alterations of their abundance in the nail proteome. A broad range of enzymes and other non-structural proteins were elevated whereas the amounts of cytoskeletal proteins of the keratin and keratin-associated protein families, cytolinker proteins and desmosomal proteins were either unaltered or decreased in nails of mice lacking epithelial autophagy. Among the various types of non-cytoskeletal proteins, the subunits of the proteasome and of the TRiC/CCT chaperonin were most strongly elevated in mutant nails, indicating a particularly important role of autophagy in removing these large protein complexes during normal cornification. Taken together, the results of this study suggest that autophagy is active during nail keratinocyte cornification and its substrate specificity depends on the accessibility of proteins outside of the cytoskeleton and their presence in large complexes.

**Electronic supplementary material:**

The online version of this article (10.1007/s10495-018-1505-4) contains supplementary material, which is available to authorized users.

## Introduction

**Fig. 1:**
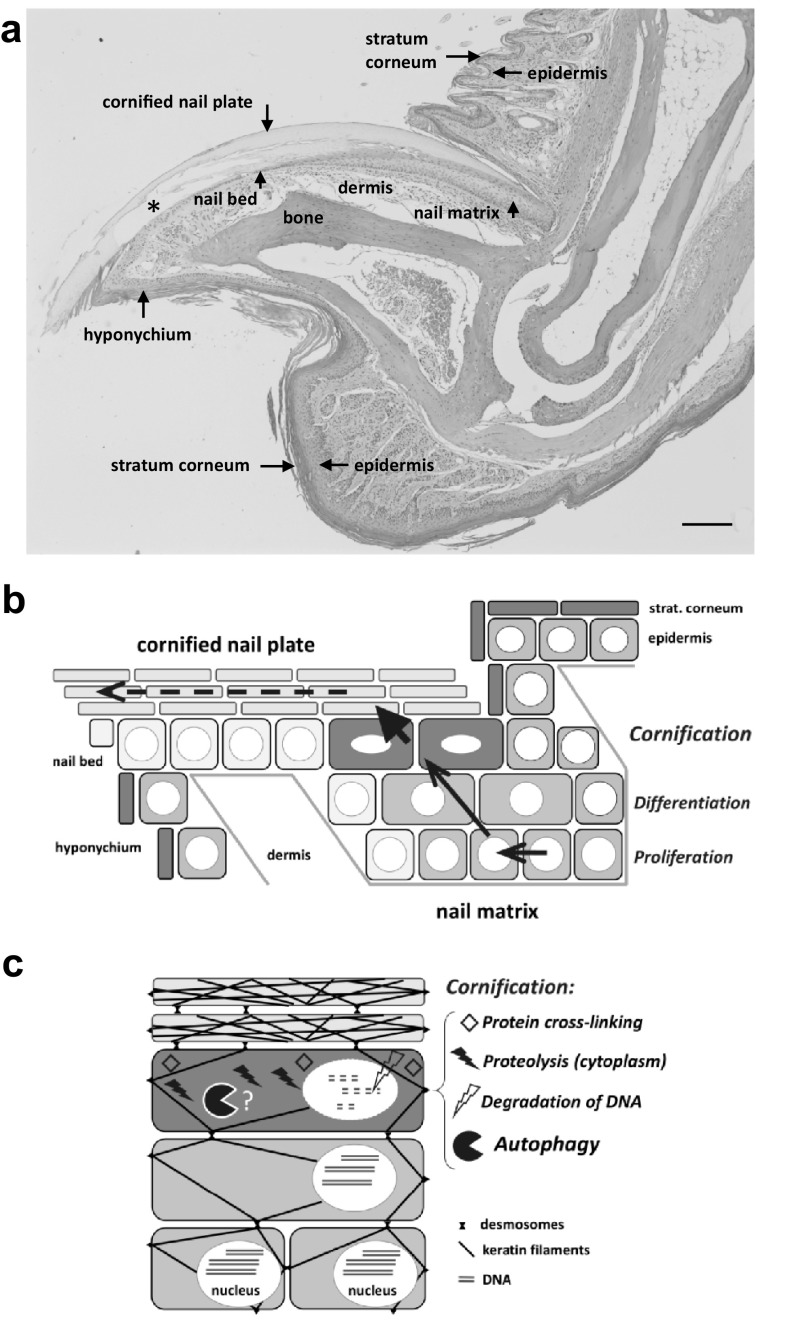
Cornification of nail keratinocytes involves intracellular remodelling. **a** Hematoxylin and eosin (H&E) staining of a sagittal section through a mouse toe. Parts of the nail plate (*) have been lost in the course of sample processing. Size bar, 200 µm. **b** Schematic depiction of keratinocyte differentiation in the nail matrix (indicated by arrows). **c** Schematic depiction of intracellular remodelling during keratinocyte differentiation and cornification. The aim of the study is to determine whether autophagy in cornifying keratinocytes influences the proteome of the cornified nail

Cornification is a special form of programmed cell death that does not lead to the disintegration or phagocytosis of dead cells but to the conversion of keratinocytes into “corneocytes” that are integrated into hard skin appendages such as nails and into the protective cornified layer (stratum corneum) on the surface of the epidermis (Fig. 1a, b) [1–4]. Corneocytes are densely packed with keratin intermediate filaments and associated proteins that are cross-linked by transglutamination and disulfide bonds. Other intracellular components of keratinocytes, including the nucleus, are removed whereas cell junctions via desmosomes are maintained during cornification (Fig. 1c). The proteolytic cleavage of cell junctions in the superficial layers of the stratum corneum causes the shedding of epidermal corneocytes (desquamation), and continued presence of intercellular connections facilitates the growth of cornified nails.

The degradative processes during cornification have similar effects as autophagy, i.e. the ubiquitous intracellular program for delivery of cellular components to lysosomes for digestion [5–8]. Autophagy was suggested to remove organelles during terminal differentiation of keratinocytes [4, 9–12] and specifically to degrade the nucleus (nucleophagy) [13]. However, the deletion of *autophagy-related 7 (Atg7)*, which is essential for macroautophagy, the main form of autophagy in mammalian cells, in keratin K14-positive epithelial cells (*Atg7*^*f*/*f*^*K14-Cre*) did not impair DNA degradation in the interfollicular epidermis of mice [14]. Suppression of epithelial autophagy even led to premature degradation of the nucleus in sebocytes [15, 16]. In addition, the stratum corneum of *Atg7*^*f*/*f*^*K14-Cre* mice was thickened [14], and the protein turnover in thymic epithelial cells [17], sweat glands [18], Merkel cells [19], and sebaceous glands [20] was altered. The autophagy adapter and substrate proteins microtubule-associated proteins 1A/1B light chain 3A (LC3) and sequestosome 1 (Sqstm1)/p62 were elevated in isolated epidermal keratinocytes upon deletion of autophagy genes [15, 21], but a comprehensive catalog of keratinocyte proteins targeted by autophagy has remained elusive.

In the present study, we determined the protein composition of the cornified nails of *Atg7*^*f*/*f*^*K14-Cre* mice and autophagy-competent control mice. We provide in vivo evidence for the hypothesis that autophagy shapes the proteome of cornifying nail keratinocytes.

## Materials and methods

### Mice

The generation of *Atg7*^*f*/*f*^*K14-Cre* (KO) mice by crossing *Atg7*^*f*/*f*^ (WT) mice [22] and *K14-cre* mice, strain Tg(KRT14-cre)1Amc/J (Jackson Laboratories, Bar Harbour, ME) on a B6/CBA background has been reported previously [14, 17]. *GFP-LC3* transgenic mice [23] were crossed with *Atg7*^*f*/*f*^*K14-Cre* mice to obtain *Atg7*^*f*/*f*^*GFP-LC3* and *Atg7*^*f*/*f*^*K14-Cre GFP-LC3* mice. Genotyping and maintenance of mice was done as reported [14]. Animal procedures were performed according to the guidelines of the Ethics Review Committee for Animal Experimentation of the Medical University of Vienna, Austria (approval number BMWF-66.009/0124-II/10b/2010).

### Nail morphology, histology, immunofluorescence and fluorescence analysis

Toes were cut off the feet of sacrificed WT and KO mice and photographed under a Leica MZ 16 stereomicroscope using a Leica IC 3D CCD camera (Leica Microsystems, Wetzlar, Germany). Tissues were fixed in formaldehyde, embedded in paraffin, sectioned and stained with hematoxylin and eosin (H&E). DNase1L2 was detected by immunofluorescence labeling according to a published protocol [24]. DNA fragments were detected in formaldehyde-fixed tissue sections by terminal deoxynucleotidyl transferase dUTP nick end labeling (TUNEL) according to a published protocol [25]. Nuclei were counter-labeled with Hoechst dye. The presence of recombinant GFP-LC3 puncta was investigated on cryosections using an LSM700 confocal laser microscope (Zeiss) [17].

### Sample preparation for proteomic analysis

Immediately after mice were sacrificed, the nails were cut off from toes. Only non-pigmented nails were used. The adhering tissue was removed by heating the samples in 2% SDS in phosphate-buffered (PBS) for 1 h. Subsequently the nails were washed in PBS 3 times followed by final washes in 70% ethanol and 100% ethanol. The nails were air-dried and stored at room temperature prior to further processing. Samples from each mouse were processed separately. The nails were incubated for 1.5 h at 70 °C in 2% SDS, 0.1 M sodium phosphate (pH 7.8), 20 mM dithioerythritol, and then alkylated with 40 mM iodoacetamide. Total protein was recovered by ethanol precipitation, rinsed in 67% ethanol and digested with reductively methylated trypsin [26] in fresh 0.05 M ammonium bicarbonate containing 5% acetonitrile for 3 days at room temperature.

### LC-MS/MS analysis

Peptides were separated on a Michrom Paradigm HPLC (Michrom Corporation) with a Michrom Easy-Spray source. The digested peptides were reconstituted in 2% acetonitrile/0.1% trifluoroacetic acid and roughly 3 µg of each sample was loaded onto a 100 micron × 25 mm Magic C18 100 Å 5U reversed-phase trap where they were desalted online before being separated on a 200 micron × 150 mm Magic C18 200 Å 3U reversed-phase column. Peptides were eluted using a gradient of 0.1% formic acid (A) and 100% acetonitrile (B) with a flow rate of 300 nL/min. A 60 min gradient was run with 5–35% B over 110 min, 35–80% B over 3 min, 80% B for 1 min, 80–5% B over 1 min, and finally held at 5% B for 5 min.

Mass spectra were collected on an Orbitrap Q Exactive mass spectrometer (Thermo Fisher Scientific) in a data-dependent mode with one MS precursor scan followed by 15 MS/MS scans. A dynamic exclusion of 15 s was used. MS spectra were acquired with a resolution of 70,000 and a target of 1 × 10^6^ ions or a maximum injection time of 30 ms. MS/MS spectra were acquired with a resolution of 17,500 and a target of 5 × 10^4^ ions or a maximum injection time of 50 ms. Peptide fragmentation was performed using higher-energy collision dissociation (HCD) with a normalized collision energy (NCE) value of 27. Unassigned charge states as well as + 1 and ions > + 5 were excluded from MS/MS fragmentation.

### Peptide spectrum matching

MS/MS data were analyzed using using X! Tandem (http://www.thegpm.org; version CYCLONE (2013.02.01.1)) assuming the digestion enzyme trypsin. X! Tandem was set up to search the Uniprot mouse database (8 July 2013, 86,032 entries) appended to an identical but reversed peptide database for estimating false discovery rate. X! Tandem was searched with a fragment ion tolerance of 20 ppm (monoisotopic) and a parent ion tolerance of 20 ppm (monoisotopic). Iodoacetamide derivative of cysteine was specified in X! Tandem as a fixed modification. Variable Modifications: N-terminal Glu → pyro-Glu and loss of ammonia, deamidation of N and Q, oxidation and dioxidation of M and W, and acetylation of the N-terminus. Scaffold (version Scaffold_4.8.4, Proteome Software, Inc., Portland, OR) was used to validate MS/MS based peptide and protein identifications. Protein probabilities were assigned by the Protein Prophet algorithm [27]. Peptide FDR: 0.5% (Decoy), Protein FDR: 2.8% (Decoy). Proteins that contained peptides that could not be distinguished by MS/MS analysis were grouped for parsimony. Peptides potentially derived from contamination by human material were removed from the analysis.

### Intensity-based absolute quantification (iBAQ)

iBAQ values of individual proteins were used to calculate the relative molar amount of each protein [28]. iBAQ values were calculated by searching the mass spectrometry data with Maxquant (1.5.7.4) against the Uniprot Mouse proteome (3AUP000000589, Dec 2017) using Maxquant’s default settings with the exception that match between runs was turned on. Peptides and proteins were filtered with a decoy false discovery rate of 1%. Maxquant MS1 intensity values and identification results were imported into Scaffold (version 4.82) and Scaffold was used to calculate the iBAQ values for each sample. To this end, average calculated values for each protein were normalized to the total values for a given genotype.

### Data availability

Mass spectrometry and proteomics data were uploaded to the Massive Proteomics data repository (massive.ucsd.edu) and Proteome Exchange (http://www.proteomexchange.org/) and are avalaible under the identification numbers MSV000082333 and PXD009663, respectively.

### Reverse transcription polymerase chain reactions (RT-PCR)

The two biggest claws of each paw were pulled out immediately after sacrificing mice (8 claws per animal). The claws and the adhering nail matrix were incubated in 1 ml TriFast (VWR, Radnor, PA) for 1 h at 4 °C. RNA was extracted from the nail matrix and, as control, of homogenized sole skin, using TriFast according to the manufacturer´s instructions and reverse-transcribed to cDNA with the Iscript™ kit (Biorad, Hercules, CA).

Transcripts of *Atg7* and the housekeeping gene *Beta2 microglobulin* (*B2m*) were amplified using DreamTaq™ DNA polymerase (Thermo Scientific, Waltham, CA) and the primer pairs Atg7_f, agcttggctgctacttctgc and Atg7_r, tcattcatgcggtcatcact, and B2m_f, attcacccccactgagactg and B2m_r, tgctatttctttctgcgtgc. *B2m* was previously established as a house-keeping gene equally expressed in WT and KO keratinocytes [29]. The PCR products were electrophoresed through 1.5% agarose gels and labeled with GelRed nucleic acid stain (Biotium, Fremont, CA).

Transcripts of the following genes were quantified by qPCR, using the LightCycler → (LC480) technology (Roche Applied Science, Basel, Switzerland) and the LightCycler 480 DNA SYBR Green I Master Kit (Roche Applied Science) according to a published protocol [30] with the indicated primer pairs: *Aloxe3* (Aloxe3_f, aggcacctgcctacaaacag and Aloxe3_r, atcagtgggcagaaagatgg), *Cct3* (Cct3_f, agtcatcagtcggtggtcct and Cct3_r, taatatagcggcgcatcctt), *Hspa5* (Hspa5_f, agcgacaagcaaccaaagat and Hspa5_r, atgacccgctgatcaaagtc), *Krt86* (Krt86_f, ggagcagaggttgtgtgagg and Krt86_r, tgcagcattgtgacctccta), *Lrrc15* (Lrrc15_f, ccgcctccttcttattgacc and Lrrc15_r, ggagttcggtgatgtgtgtg), *Lyg1* (Lyg1_f, tggggatgctatggaaacat and Lyg1_r, cgaccacatagttgcctcct), *Pdia3* (Pdia3_f, tatgatgggcctaggactgc and Pdia3_r, tgctggctgcttttaggaat), *Pdia6* (Pdia6_f, gccaccatgaatcaggttct and Pdia6_r, ctctggaggaggggcattat), and *Psmd2* (Psmd2_f, agcaggagctgtctgaggag and Psmd2_r, ggacgcagaaatttgagagg). The relative expression of each gene was normalized to that of *B2m*, which was amplified with the primer pair indicated above and quantified using a mathematical model described previously [31].

### Statistical analysis

Data are presented as mean ± standard deviation (SD). Standard deviations of subtractions (KO-WT) and divisions (KO/WT) of means were calculated according to the rules of error propagation. The significance of difference between groups was determined with the two-tailed t-test. To reduce effects of multiple testing, we focused our comparative analysis on a small number of protein groups instead of a large number of individual proteins and based major conclusions only on differences supported by *p* values smaller than 0.01 instead of the standard *p* < 0.05.

## Results

### *Atg7*-deficiency does not abrogate differentiation of nail keratinocytes in *Atg7*^*f*/*f*^*K14-*Cre mice

As the constant turnover of proteins in living cells limits the power of proteomic analysis, we chose the cornified nails as a model system in which the proteome of nail-forming cells is “frozen” by protein cross-linking. To study the role of autophagy in the cornification process, we compared *Atg7*^*f*/*f*^ (wildtype, WT) and *Atg7*^*f*/*f*^*K14-Cre* (knockout, KO) mice in which the essential autophagy factor *Atg7* is deleted in keratinocytes [14].

**Fig. 2:**
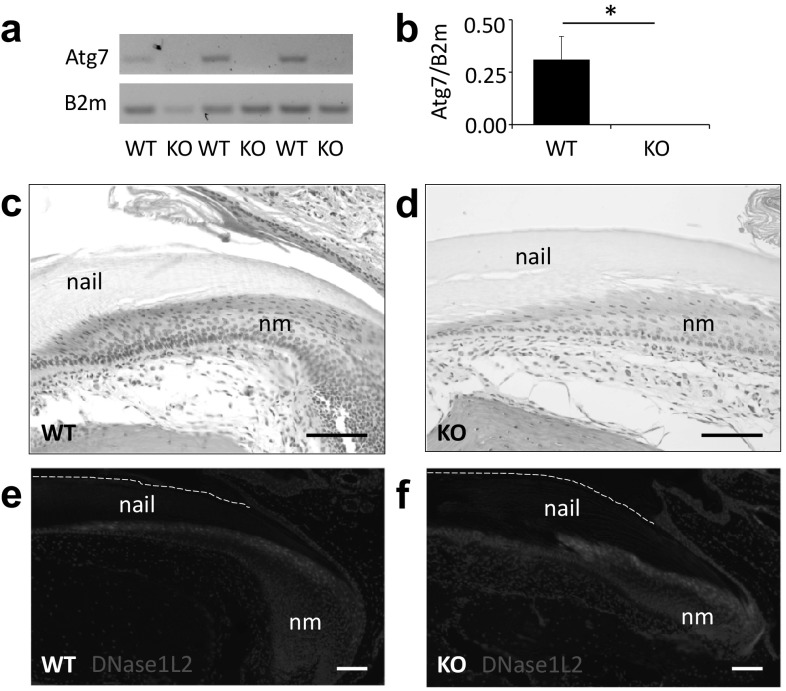
K14-Cre-mediated deletion of *Atg7* in the nail matrix is compatible with histologically normal nail formation. **a** RT-PCR analysis of *Atg7* and *B2m* expression in the nail matrix of mice carrying floxed *Atg7* alleles either in the absence (WT) or presence (KO) of *K14-Cre*. **b** RT-PCR band intensities were quantified. The graph shows the means of the intensity ratios *Atg7*/*B2m*, error bars show standard deviations. The ratio was significantly reduced (*p* < 0.05, 2-sided t test, *) in *Atg7*^*f*/*f*^*K14-Cre* (KO) mice. **c**, **d** H&E staining of the nail matrix (nm) of *Atg7*^*f*/*f*^ (WT) and *Atg7*^*f*/*f*^*K14-Cre* (KO) mice. **e**, **f** Immunofluorescence labeling of DNase1L2 (red) in the nail matrix. Scale bars, 100 µm

To determine the expression of *Atg7* in nail keratinocytes, we extracted RNA from cells of the nail matrix of *Atg7*^*f*/*f*^ (wildtype, WT) and *Atg7*^*f*/*f*^*K14-Cre* (knockout, KO) mice and performed RT-PCR. Atg7 was readily detected in the nail matrix of WT but not of KO mice, confirming efficient inactivation of *Atg7* expression in the cells that form nails (Fig. 2a, b). To monitor autophagosomes *in situ*, the transgenic reporter protein GFP-LC3 was introduced into a subline of *Atg7*^*f*/*f*^ and *Atg7*^*f*/*f*^*K14-Cre* mice. GFP-LC3-labelled autophagosomes were detected in the nail matrix of WT (*Atg7*^*f*/*f*^*GFP-LC3*) mice (Suppl. Fig. S2a, b) but not in the nail matrix of KO (*Atg7*^*f*/*f*^*K14-Cre GFP-LC3*) mice (Suppl. Fig. S2c), confirming the suppression of autophagy upon epithelial deletion of *Atg7*. Terminal deoxynucleotidyl transferase dUTP nick end labeling (TUNEL) showed that the blockade of autophagy did not lead to premature cell death in differentiating nail keratinocytes (Suppl. Fig. S3). TUNEL-positive DNA 3´-OH ends remained confined to the layer of cornifying keratinocytes adjacent to the nail plate in both WT and KO mice (Suppl. Fig. S3).

WT and KO mice were maintained under standard housing conditions in which the nails are exposed to only weak mechanical stress. The nails of both mouse genotypes had the same shape, suggesting that growth and wear and tear were not altered by the deletion of *Atg7* (Suppl. Fig. S1). H&E staining of sagittal sections through the nail unit showed the normal stages of keratinocyte differentiation including a cornification-associated change from eosinophilic keratinocytes to essentially unstained nail corneocytes in both WT and KO mice (Fig. 2c, d). Immunofluorescence labeling demonstrated normal expression of the terminal differentiation marker DNase1L2 in the nail matrix and spatial correlation of DNase1L2 expression with the breakdown of nuclear DNA in nails of both genotypes (Fig. 2e, f; Suppl. Fig. S3) [32, 33]. Gene expression analysis by quantitative RT-PCR showed that expression of DNase1L2 and other genes in the nail matrix was not significantly different between WT and KO mice (Suppl. Fig. S4). Thus deletion of *Atg7* was compatible with the normal execution of the genetic program of nail keratinocyte differentiation.

### Suppression of autophagy leads to an increase in protein diversity and a decrease of the fraction of cytoskeletal proteins in nails

To determine the role of autophagy in the re-shaping of the proteome during cornification, 3 WT and 3 KO mice were sacrificed and cornified nails were prepared, taking care to remove all surrounding tissue. The protein composition of the nails of each mouse was determined using a proteomics approach described previously for cornified skin and appendages [34, 35]. In brief, the proteins were vigorously extracted with SDS buffer containing dithioerythritol to break disulfide bonds followed by digestion with trypsin, separation by reversed-phase chromatography and tandem mass spectrometry (MS). Relative amounts of a given protein in different samples were compared by intensity-based absolute quantification (iBAQ) [28].

In nails of WT mice, a total of 518 ± 11 (mean ± SD) proteins were identified whereas 633 ± 2 proteins were identified in nails of KO mice (Suppl. Table S1), representing an increase of the protein diversity by more than 20% (*p* < 0.01, t test). Similarly, the number of highly abundant proteins (iBAQ > 10^7^) was increased by 20% in KO nails relative to WT nails (WT, n = 284; KO, n = 345; *p* < 0.01).

**Fig. 3:**
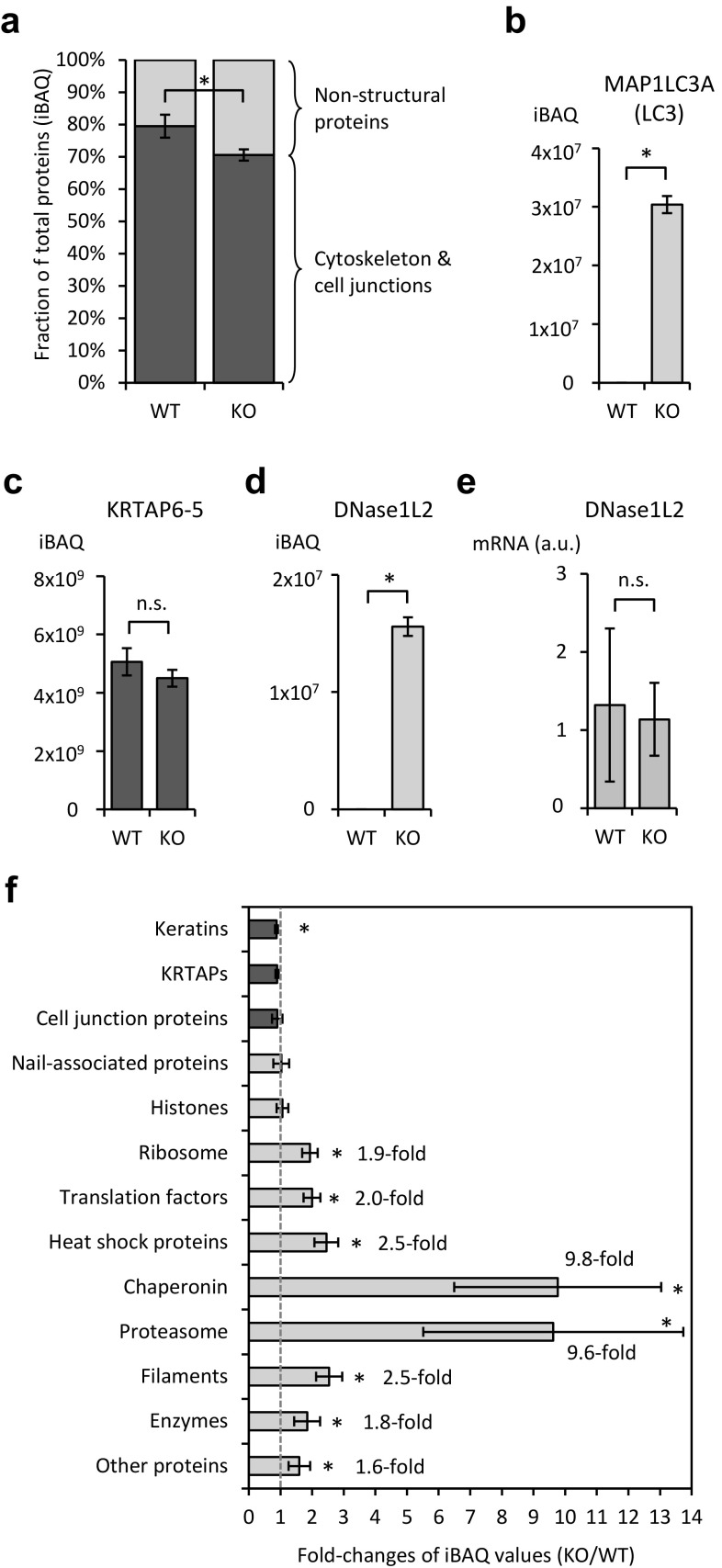
Knockout of *Atg7* in keratinocytes leads to significantly increased abundance of non-structural proteins in cornified nails. **a** iBAQ values of structural proteins (keratins, KRTAPs, cell junctions) (dark blue) and other proteins (light blue) in WT and KO nails. **b–d** iBAQ values of MAP1LC3A, KRTAP6-5, and DNase1L2 in WT and KO nails. **e** Real-time PCR quantification of DNase1L2 mRNAs in the nail matrix of WT and KO mice (a.u., arbitrary units). The graphs **a–e** show the means (n = 3 per genotype) and the error bars indicate standard deviations. *, *p* < 0.05 (t-test); n.s., not significant. **f** Fold-changes of iBAQ values corresponding to groups of proteins in KO *versus* WT nails. The vertical grey dotted line marks a fold-change of 1 (equal abundance in WT and KO nails). The graph shows values calculated from means and the error bars indicate standard deviations. (Color figure online)

To determine the influence of autophagy on the abundance of different proteins in cornified nails, we compared the iBAQ values of protein groups and individual proteins in the nails from WT and KO mice. First, we categorized proteins according to their contribution to the primary feature of nails, that is mechanical resilience. This characteristic trait of nails depends on stable protein structures that consist of the keratin cytoskeleton and stable cell junctions. As a proxy, we calculated the sum of iBAQ values of “structural proteins” including keratins, keratin-associated proteins (KRTAPs), and cell junction proteins (desmosome and cytolinker proteins), relative to all other proteins, here termed “non-structural proteins” (Table 1). These structural proteins accounted for 79 ± 4% (mean ± SD) of the iBAQ values of WT nails but only for 71 ± 2% of the iBAQ values of nails formed by Atg7 KO keratinocytes (Fig. 3a). Conversely, 21 ± 4% and 29 ± 2% iBAQ values corresponded to non-structural proteins in WT and KO nails, respectively, with the difference being statistically significant. The ratio of iBAQ values of non-structural *versus* structural proteins was increased from 0.26 ± 0.06 in WT nails to 0.42 ± 0.03 in KO mice (*p* < 0.01).

**Table 1:** Proteomics of WT and *Atg7* KO mouse nails

Protein group	Protein abundance (iBAQ /1E + 06)^a^						Abundance, mean		Abundance (%)		Statistics^b^	
	WT1	WT2	WT3	KO1	KO2	KO3	WT	KO	WT	KO	*p* value	sign
Keratins	33,210	30,099	33,914	29,132	28,246	27,479	32,408	28,285	27.56%	24.08%	0.0312	*
KRTAPs	63,897	56,865	60,692	56,250	53,144	52,928	60,485	54,107	51.44%	46.06%	0.0501	n.s
Cell junction proteins	464	698	593	469	571	539	585	526	0.50%	0.45%	0.4697	n.s
Nail-associated proteins	5546	8206	5990	5365	6746	8132	6581	6748	5.60%	5.74%	0.8913	n.s
Histones	4692	6911	5184	5913	6259	5646	5596	5940	4.76%	5.06%	0.6471	n.s
Ribosome	2786	3721	3098	6088	6468	5992	3202	6183	2.72%	5.26%	0.0007	*
Translation factors	749	1008	894	1628	1842	1818	883	1763	0.75%	1.50%	0.0010	*
Heat shock proteins	261	365	290	707	740	798	305	748	0.26%	0.64%	0.0004	*
Chaperonin	5	9	12	77	98	88	9	87	0.01%	0.07%	0.0002	*
Proteasome	6	20	13	114	128	139	13	127	0.01%	0.11%	0.0001	*
Filaments	443	642	515	1282	1333	1442	533	1353	0.45%	1.15%	0.0004	*
Enzymes	1462	2375	1666	3134	3383	3620	1834	3379	1.56%	2.88%	0.0076	*
Other proteins	4115	6558	4786	7463	8415	8795	5153	8224	4.38%	7.00%	0.0208	*
Total	117,637	117,478	117,647	117,623	117,372	117,415	117,587	117,470	100.00%	100.00%	0.2838	n.s

*iBAQ* Intensity-based absolute quantification, *KO* knockout, *KRTAP* keratin-associated protein, *n.s*. not significant, *sign* significance, *WT* wildtype

^a^Protein abundance corresponds to the sum of the iBAQ values divided by 10^6^

^b^Statistic comparisons were made using the 2-tailed t-test. *, significant

At the level of individual proteins, the autophagy adapter and substrate protein LC3 (microtubule-associated proteins 1A/1B light chain 3A, MAP1LC3A) was significantly increased in KO nails (Fig. 3b), confirming an effective blockade of autophagic turnover in Atg7-deficient keratinocytes. The abundance of all structural proteins, as illustrated by the example of KRTAP6-5 in Fig. 3c, was uniformly decreased in KO mouse nails (Suppl. Table S1) whereas Atg7-dependent changes in abundance of non-structural proteins were more variable, as will be described in detail in the next section, and included multiple cases of more than 10-fold elevations (Suppl. Table S1). DNase1L2, which is expressed in differentiated keratinocytes of both genotypes (Fig. 2e, f), was absent in the proteome of cornified WT nails, indicating degradation of DNase1L2 during cornification, but abundantly present in KO nails (Fig. 3d). The increase of DNase1L2 protein levels in KO nails was not caused by an elevation of *Dnase1l2* gene expression in the nail matrix, because equal amounts of DNase1L2 mRNA were detected by quantitative RT-PCR in WT and KO mice (Fig. 3e). Likewise, other changes in protein abundance were not accompanied by changes in the levels of corresponding mRNAs (Suppl. Fig. S4), suggesting that the K14-Cre-mediated deletion of *Atg7* altered the composition of nails by affecting protein turnover but not gene expression in the nail matrix.

### Subunits of the proteasome and chaperonin are most strongly elevated upon genetic suppression of epithelial autophagy

We further classified proteins according to their roles in nail matrix cells (e.g., ribosomal proteins, translation factors [translation initiation and elongation proteins], proteasomal proteins, and enzymes) and compared the abundance (iBAQ values) of these protein groups in WT and KO nails. Keratins and KRTAP were the predominant proteins in both WT and KO nails but their share of total peptides detected (iBAQ) was reduced by 3.5% and 5.4%, respectively, in KO nails (Table 1) (Suppl. Fig. S5). This corresponded to fold-changes of 0.87 and 0.90, respectively (Fig. 3f). Histones and “nail-associated proteins” LRRC15 and LYG1, expressed in the nail matrix but not in the epidermis of the sole (Suppl. Fig. S4), were the next most abundant protein groups. The iBAQ values of these proteins were not significantly different in WT and KO nail samples (Table 1; Fig. 3f).

The decrease in the abundance of keratins and KRTAPs was balanced by increases in the abundance of 8 groups of non-structural proteins: Ribosomal proteins, translation factors, chaperones, chaperonin, proteasomal proteins, filament proteins (microfilaments, microtubules, and associated proteins), enzymes, and the remaining other proteins increased by at least 50% of their respective iBAQ value when autophagy was suppressed, and the increases were significant (*p* < 0.05) for all these protein groups, hereafter referred to as putative autophagy substrates.

**Fig. 4:**
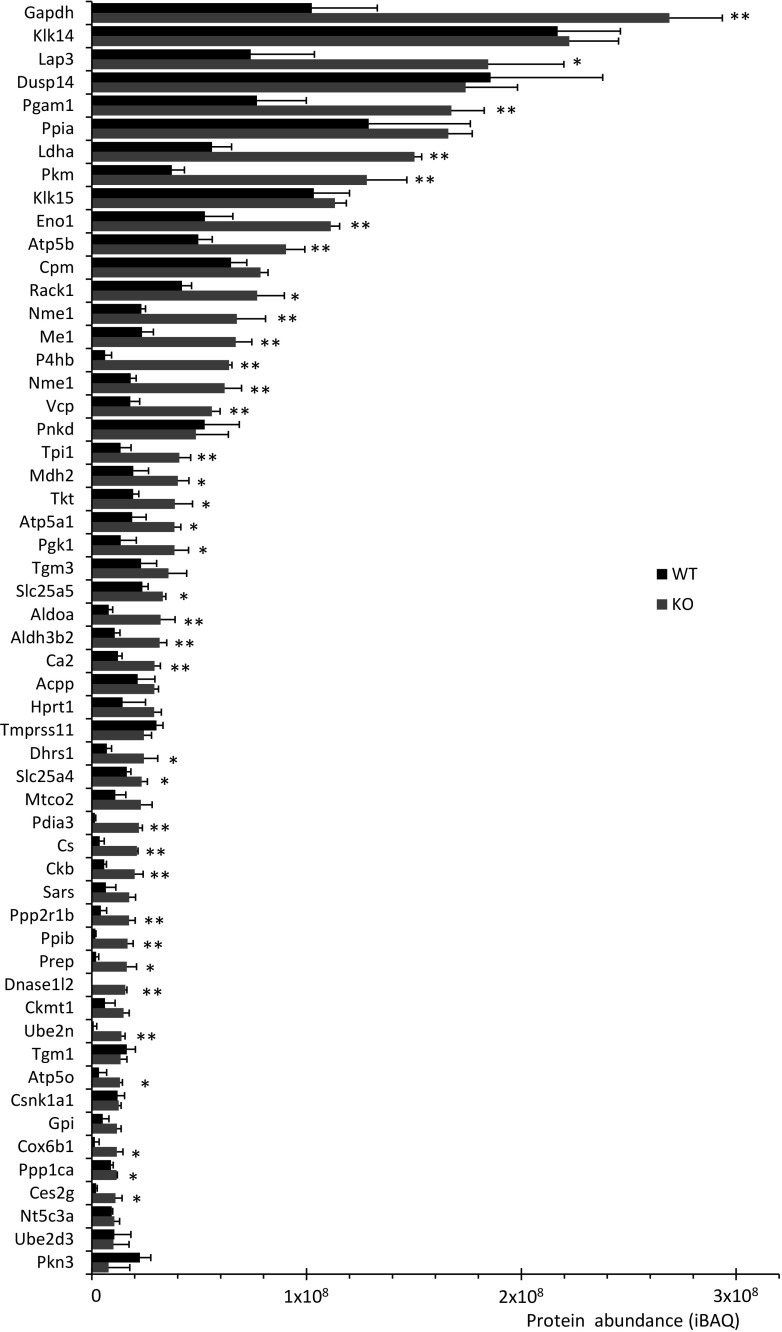
Cornified nails of *Atg7* KO mice contain aberrantly high amounts of functionally diverse enzymes. The graph shows the abundance of enzymes that have a mean iBAQ value of at least 10^7^ in either WT (black bars) or KO (red bars) nails (n = 3 per genotype). Error bars indicate standard deviations. *, *p* < 0.05; **, *p* < 0.01 (two-tailed t-test). (Color figure online)

Ribosomal proteins were present at higher abundance than any other type of putative autophagy substrates in WT nails, and at even at higher abundance in KO nails (Table 1). The genetic suppression of autophagy increased their share (based on iBAQ) of the total proteins from 2.7 to 5.3%, representing a 1.9-fold elevation (Fig. 3f). The finding that 73 of 76 detectable ribosomal proteins yielded higher iBAQ values in KO than WT nails confirmed the significance of this change. The largest group of putative autophagy substrates, according to our classification, were the enzymes. Among 177 enzymes detected in at least one of the nail samples, 155 (83% of all enzymes) were elevated in the absence of keratinocyte autophagy (Fig. 4).

**Fig. 5:**
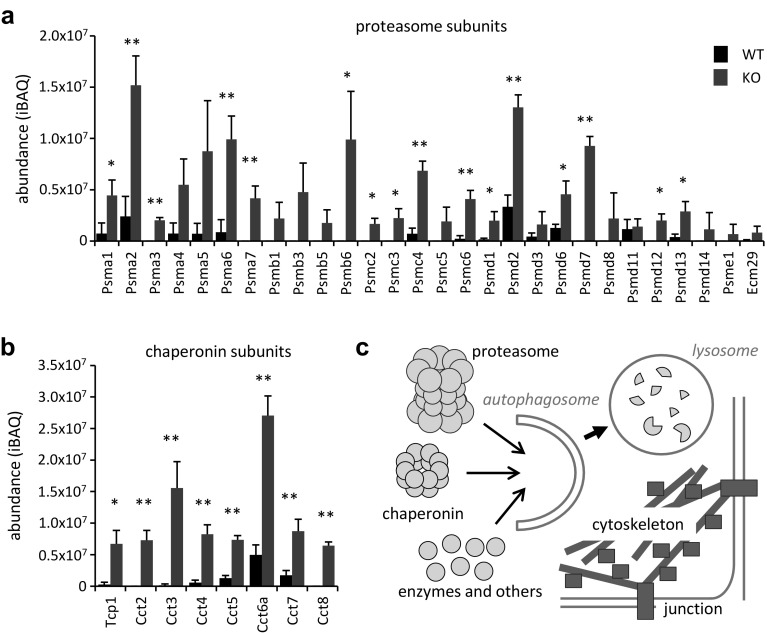
Proteasomal proteins and all subunits of the CCT chaperonin accumulate in cornified nails of *Atg7* KO mice. **a** iBAQ values of proteasome subunits in WT (black bars) and KO (red bars) nails. **b** iBAQ values of chaperonin subunits in WT (black bars) and KO (red bars) nails. Error bars in **a** and **b** indicate standard deviations. *, *p* < 0.05; **, *p* < 0.01 (two-tailed t test). **c** Schematic model of autophagic degradation of non-structural proteins in cornifying keratinocytes. (Color figure online)

Among the proteins accumulating in nails of KO mice, the subunits of two large protein complexes stood out by particularly strong increases: the subunit proteins of CCT/TRiC chaperonin, which increased 9.8-fold and the protein components of proteasomes, which increased 9.6-fold (Fig. 3f). 8 of 8 chaperonin components and 28 of 28 detectable proteasome components were present at increased levels when autophagy was suppressed (Fig. 5a, b). The proteasome and the CCT/TRiC chaperonin were previously reported as preferential substrates of basal autophagy in cultured cells [36].

Thus, our results are compatible with the hypothesis that, in normal nail keratinocytes, a wide variety of non-structural proteins is degraded by autophagy in a manner that is primarily determined by the accessibility of substrates, a process known as bulk autophagy (Fig. 5c), whereas deletion of *Atg7* suppresses this process and leads to aberrant retention of non-structural proteins in nails of KO mice.

## Discussion

The results of this study suggest that autophagy contributes to the intracellular remodeling during cornification of nail keratinocytes and provide new insights into the substrate specificity of autophagy in vivo. We utilized nail cornification as a model system because the products of this type of cornification, i.e. nails, can be prepared in pure form by removing non-cornified material, and remain stable during storage prior to analysis. Accordingly, the variation between mass spectrometry results of nail samples from mice of the same genotype was low and the identification of autophagy-dependent changes in the abundance of numerous proteins was possible.

The deletion of the essential autophagy gene *Atg7* in epithelial cells of *Atg7*^*f*/*f*^*K14-Cre* (KO) mice was demonstrated in previous studies [14, 17]. Here, we confirmed the expectation that expression of *Atg7* is also abrogated in the nail matrix (Fig. 2a), where keratinocytes are converted by cornification into building blocks of the nail. It is likely that autophagy is normally active in more than one or even in all differentiation stages (from the K14-positive stem cell stage to cornification) and, consequently, disturbances in protein turnover accumulate and lead to an altered nail proteome in KO mice. In agreement with the concept of an autophagy-dependent proteome phenotype, changes in the abundance of several nail proteins were not caused by changes in the levels of the corresponding mRNAs in the nail matrix (Suppl. Fig. S4).

Non-structural proteins of many functions from the synthesis of proteins to catalysis of metabolic reactions were elevated in nails of KO mice, arguing against a receptor-dependent specificity of autophagy during nail keratinocyte differentiation. Previous proteomics-based screening for autophagy substrates have provided evidence both for bulk degradation of cytoplasmic proteins (bulk autophagy), similar to the broad spectrum of functionally unrelated enzymes in our study (Fig. 4), and for substrate-specific receptor-mediated autophagy (selective autophagy) [37–42]. In nail keratinocytes, some proteins were more strongly affected by autophagy (or its absence) than others. The multiprotein complexes of the CCT chaperonin and the proteasome displayed the highest relative accumulation in the absence of autophagy. These results are in agreement with findings of a protein flux study [36] in which the proteasome and the CCT/TCP-1/TRiC chaperonin were demonstrated to be substrates of basal autophagy in cultured cells. Thus our study provides in vivo support for a hypothesis raised by *in vitro* data. Interestingly, autophagic degradation of proteasomes was recently shown to be an evolutionarily conserved process [43].

The turnover of large protein complexes such as chaperonin and the proteasome depends perhaps entirely on autophagy whereas many isolated proteins are degraded by both Atg7-dependent autophagy and proteasomes. Therefore, suppression of autophagy leads to particularly strong accumulations of large protein complexes in Atg7 KO nails and variable accumulations of other proteins depending on the accessibilities to autophagosomes and on the existence of alternative modes of breakdown for particular proteins. We propose that nail keratinocyte cornification employs bulk autophagy without signs of substrate-specific receptor-mediated degradation mechanisms. However, the precise degradation pathways of individual keratinocyte proteins remain to be dissected.

The elevations of proteasomes, chaperonin, and ribosomes in the absence of autophagy do not necessarily indicate increased activities of these molecular machines in differentiating keratinocytes and certainly not in corneocytes. However, it is at least conceivable that increased proteasomal degradation of specific proteins contributes to the proteome imbalance observed in Atg7 KO nails. In this regard it is interesting that keratins and KRTAPs are substrates of proteasomes [44, 45] and, accordingly, elevation of proteasomal activity may decrease the amounts of keratins in KO keratinocytes and nails. The increased concentration of large protein complexes, in particular ribosomes, may also influence the intracellular milieu independently of their activity. A twofold difference in the concentration of ribosomes, corresponding to the increase of ribosomal protein abundance in Atg7 KO corneocytes (Fig. 3f), was shown to enhance protein phase separation in the cytoplasm [46], a physical process likely relevant for cornification [47].

The decrease in the keratin and KRTAP fraction is perhaps the physiologically most important phenotype of the cornified nails in Atg7 KO mice. Keratins must be spared from autophagic degradation processes in WT nail keratinocytes to facilitate their function as cytoskeletal proteins while other proteins become dispensable, if not obstructive, in corneocytes. The activation of autophagy prior to death of cultured cells was previously shown to be compatible with the maintenance of the cytoskeleton whereas apoptosis led to the breakdown of cytoskeletal proteins [48]. Similarly, our results show that cytoskeletal proteins are maintained during autophagic protein turnover in cornifying keratinocytes in vivo and, in line with other articles [2, 49, 50], refute the hypothesis that cornification of keratinocytes is related to apoptosis. In Atg7 KO nails, the cytoskeletal protein content is reduced due to the incomplete degradation of non-structural proteins and possibly due to enhanced breakdown (via proteasomes) of keratins and KRTAPs. Keratins and associated proteins are the predominant material component of nails and the partial replacement of these proteins by non-structural proteins may cause defects in hardness of nails. However, under normal housing conditions there was no overt change in the shape of nails that would indicate increased abrasion or mechanically induced deformation in Atg7 KO mice (Suppl. Fig. S1). As mouse nails are too small and irregular in shape for standard assays of material hardness, sophisticated in vitro measurements or in vivo stress tests of nails remain to be applied to test whether autophagy contributes substantially to the hardening of the nail during cornification. It will also be interesting to subject the stratum corneum of WT and KO mice to comprehensive comparative proteomics and to perform stress tests that go beyond the basic characterization performed previously [14].

This study utilized nails primarily as a model system of cornification with many features advantageous for proteomics. Besides providing new insights into the mechanisms of intracellular remodeling during keratinocyte cornification, the results reported here may also have implications on therapies and diagnosis of skin diseases. Drugs that suppress autophagy, such as hydroxychloroquine, and drugs that increase autophagic activity, such as mTOR inhibitors, are currently being used or tested for the treatment of a variety of skin diseases including some with cornification abnormalities [51, 52]. Inhibitors of mTOR, which affect autophagy and other processes, are associated with nail dystrophy [53], pointing to dysregulation of cornification-associated autophagy as a potentially relevant side-effect of these drugs. In patients with psoriasis, keratinocytes forming the epidermal stratum corneum are affected by deregulation of autophagy [13, 54], suggesting that modulation of autophagy might be used in therapies of skin inflammation or cornification defects. Finally, our demonstration that suppression of autophagy correlates with changes in nail protein composition indicates that proteomic analysis of nails, and perhaps other cornified epidermal structures, may be developed further to uncover disease-associated aberrations in protein turnover for diagnostic applications.

In conclusion, this study suggests that autophagy is active during normal nail formation and that suppression of autophagy alters the proteome of cornified nails with differential effects on cytoskeletal and non-cytoskeletal proteins.

## Electronic supplementary material

Below is the link to the electronic supplementary material.

Supplementary material 1 (PDF 3001 KB)
