# Supplementary material for: Cornification of nail keratinocytes requires autophagy for bulk degradation of intracellular proteins while sparing components of the cytoskeleton
Source: Apoptosis. 2018 Dec 14;24(1):62–73. doi: 10.1007/s10495-018-1505-4 (PMC6373260; doi:10.1007/s10495-018-1505-4)
Supplement: Supplementary file 1 — Supplementary material 1 (PDF 3001 KB) [file 10495_2018_1505_MOESM1_ESM.pdf]

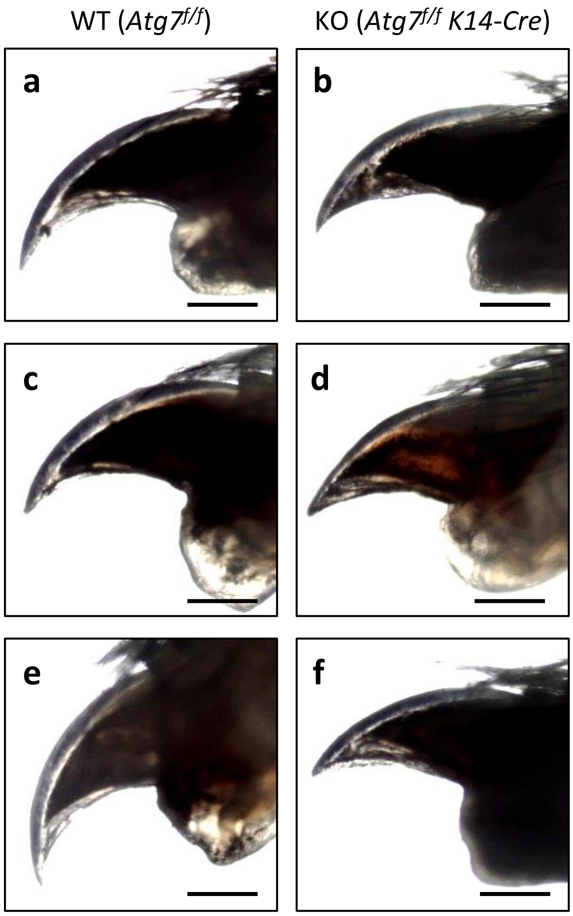

Supplementary Figure S1. Shape of nails of WT and Atg7 KO mice. Toes were prepared from hindlimbs of sacrificed mice (WT: a, c, e; KO: b, d, f) and photographed under a stereomicroscope. Three representative nails per genotype are shown. More than 10 mice per genotype were investigated. There were no consistent differences in nail shape between the genotypes. Size bars, 500 µm.

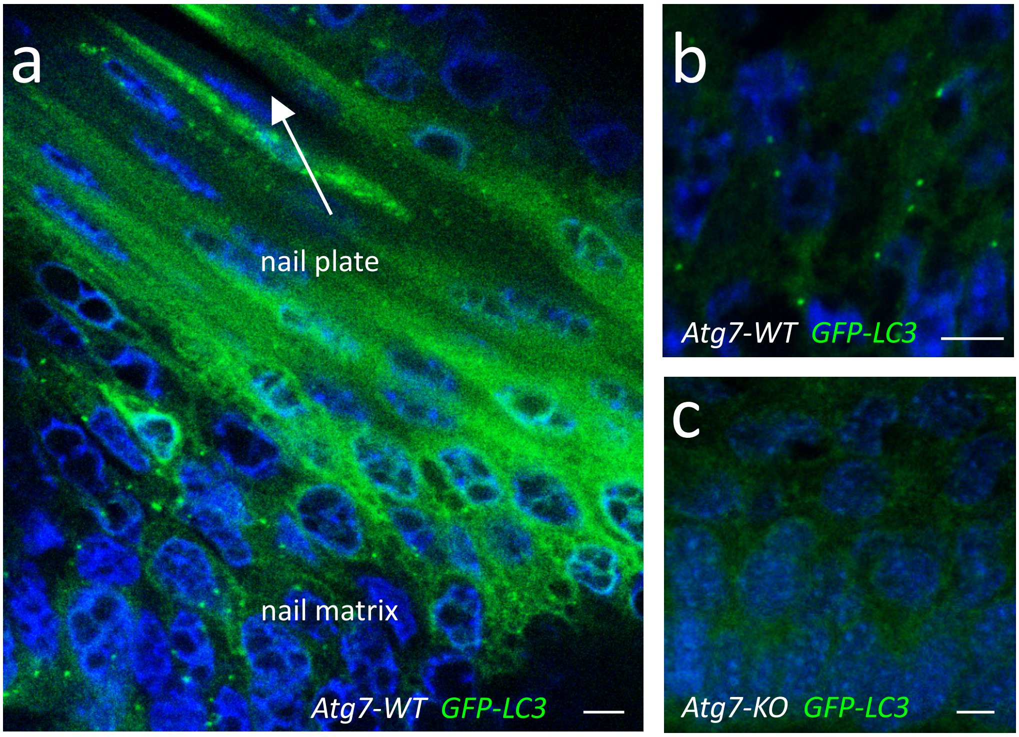

Supplementary Figure S2. GFP-LC3 puncta indicate autophagosomes in nail matrix keratinocytes of *Atg7$^{f/f}$ GFP-LC3* but not *Atg7$^{f/f}$ K14-Cre GFP-LC3* mice. The transgene *GFP-LC3* (Mizushima et al., 2004), encoding the recombinant fusion of green fluorescent protein (GFP) and the autophagy adapter LC3, was introduced into *Atg7$^{f/f}$* and *Atg7$^{f/f}$ K14-Cre* mice as reported previously (Sukeree et al., 2012). The nail matrix samples of *Atg7$^{f/f}$ (Atg7-WT) GFP-LC3* (**a**, **b**) and *Atg7$^{f/f}$ K14-Cre* (*Atg7-KO*) *GFP-LC3* (**c**) mice were embedded in optimal cutting temperature (OCT) compound in liquid nitrogen. Cryosections were counterstained with DNA-specific Hoechst dye (blue) and viewed under a laser scanning microscope. GFP-LC3-labeled autophagosomes are visible as green puncta. Note the absence of puncta in the nail matrix of *Atg7-KO GFP-LC3* mice. Scale bars, 5 µm.

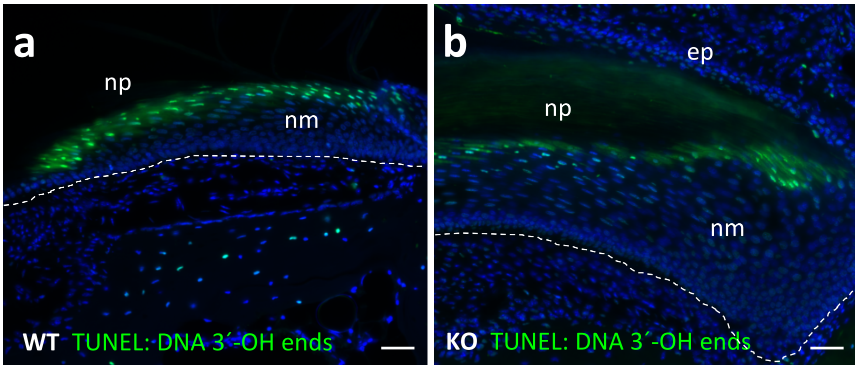

Supplementary Figure S3. Cell death occurs during cornification of nail keratinocytes but not in cells of the nail matrix in *Atg7*$^{f/f}$ (*WT*) and *Atg7*$^{f/f}$ *K14-Cre* (*KO*) mice. Formaldehyde-fixed and paraffin-embedded toes of WT (**a**) and KO (**b**) mice were sectioned and subjected to terminal deoxynucleotidyl transferase dUTP nick end labeling (TUNEL) according to a published protocol (Fischer et al., 2005). DNA 3´-OH ends are labeled green. DNA was labeled with Hoechst dye (blue). The images are representative for n=3 mice per genotype. The dermo-epidermal junction is indicated by a broken white line. ep, eponychium; nm, nail matrix; np, nail plate. Scale bars, 20 µm.

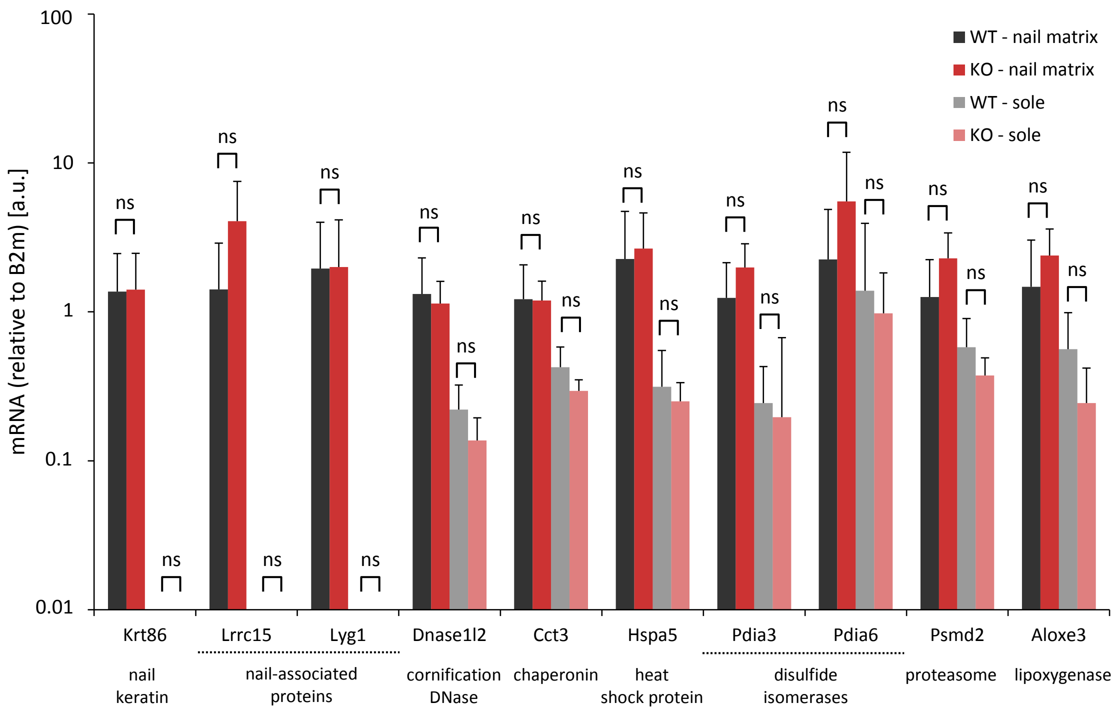

Supplementary Figure S4. Quantification of mRNAs in the nail matrix and sole skin. RNA was extracted from the nail matrix (cells attaching to isolated nails) and from the sole skin of WT and KO mice (n=5 per genotype), reverse-transcribed, and subjected to quantitative PCR (qPCR) as described in the Methods section. The mRNA levels of the target genes relative to mRNA of the house-keeping gene *B2m* [29] are shown. qPCR Ct values of *B2m* were not significantly different between WT and KO samples from the same tissue (nail matrix or sole). Bars show means and error bars show standard deviations. a.u., arbitrary units; n.s., not significant (*P*>0.05, 2-sided t-test).

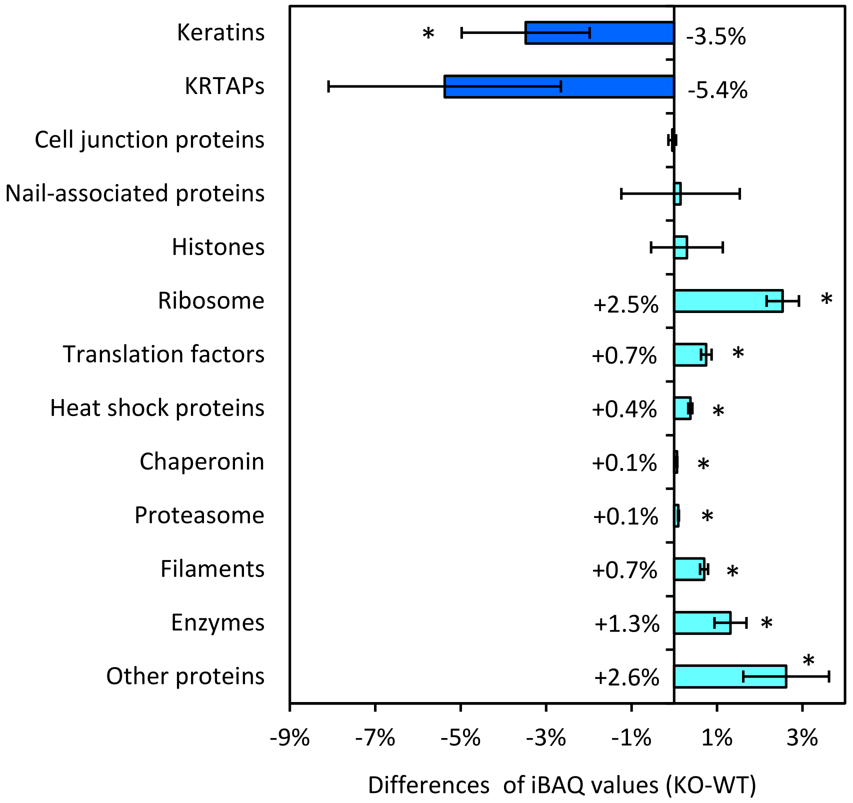

Supplementary Figure S5. The abundance of non-structural proteins is significantly increased in cornified nails of Atg7 KO (*Atg7$^{f/f}$ K14-Cre*) mice. Bars indicate the difference in the abundance (% of total IBAQ values) of the main protein groups in KO and WT nails, as indicated in Table 1. 2-tailed t-test. *, significantly different (p<0.05, t-test). IBAQ, intensity-based absolute quantification; KO, knockout; KRTAP, keratin-associated protein; WT, wildtype.

#	Identified proteins	Protein group	Accession number	Symbol	iBAQ						
					WT1	WT2	WT3	KO1	KO2	KO3	Ratio KO/WT
1	**Cluster of Keratin, type II cuticular Hb1 OS=Mus musculus GN=Krt81 PE=2 SV=2 (KRT81_MOUSE)**	Keratins	**KRT81_MOUSE [8]**	**Krt81 Cluster**	1,46E+10	1,23E+10	1,49E+10	1,19E+10	1,30E+10	1,12E+10	0,86
1,1	Keratin, type II cuticular 87 OS=Mus musculus GN=Krt87 PE=2 SV=2	Keratins	KRT87_MOUSE	Krt87	1,43E+10	1,15E+10	1,48E+10	1,14E+10	1,25E+10	1,07E+10	0,85
1,2	Keratin, type II cuticular Hb5 OS=Mus musculus GN=Krt85 PE=1 SV=2	Keratins	KRT85_MOUSE	Krt85	9,05E+09	6,89E+09	9,71E+09	6,69E+09	7,25E+09	6,34E+09	0,79
1,3	Keratin, type II cuticular Hb6 OS=Mus musculus GN=Krt86 PE=2 SV=2	Keratins	KRT86_MOUSE	Krt86	1,69E+10	1,51E+10	1,72E+10	1,43E+10	1,60E+10	1,34E+10	0,89
1,4	Keratin 83 OS=Mus musculus GN=Krt83 PE=1 SV=1	Keratins	E9Q1Y9_MOUSE (+1)	Krt83	1,62E+10	1,33E+10	1,57E+10	1,30E+10	1,37E+10	1,21E+10	0,86
1,5	Keratin, type II cuticular Hb1 OS=Mus musculus GN=Krt81 PE=2 SV=2	Keratins	KRT81_MOUSE	Krt81	1,68E+10	1,48E+10	1,72E+10	1,43E+10	1,58E+10	1,33E+10	0,89
2	**Cluster of Keratin, type I cuticular Ha3-II OS=Mus musculus GN=Krt33b PE=1 SV=2 (KT33B_MOUSE)**	Keratins	**KT33B_MOUSE [11]**	**Krt33b Cluster**	1,25E+10	1,22E+10	1,28E+10	9,74E+09	1,07E+10	1,17E+10	0,85
2,1	Keratin, type I cuticular Ha3-II OS=Mus musculus GN=Krt33b PE=1 SV=2	Keratins	KT33B_MOUSE	Krt33b	1,36E+10	1,37E+10	1,36E+10	1,09E+10	1,14E+10	1,28E+10	0,86
2,4	Keratin, type I cuticular Ha3-I OS=Mus musculus GN=Krt33a PE=1 SV=1	Keratins	KT33A_MOUSE	Krt33a	1,15E+10	1,06E+10	1,20E+10	8,60E+09	9,97E+09	1,05E+10	0,85
3	**Cluster of Keratin, type II cuticular Hb4 OS=Mus musculus GN=Krt84 PE=2 SV=2 (KRT84_MOUSE)**	Keratins	**KRT84_MOUSE [12]**	**Krt84 Cluster**	1,02E+09	1,07E+09	1,08E+09	1,30E+09	1,11E+09	9,82E+08	1,07
3,1	Keratin, type II cuticular Hb4 OS=Mus musculus GN=Krt84 PE=2 SV=2	Keratins	KRT84_MOUSE	Krt84	1,71E+09	1,82E+09	1,79E+09	1,82E+09	1,92E+09	1,55E+09	0,99
3,2	Keratin 90 OS=Mus musculus GN=Krt90 PE=1 SV=1	Keratins	E9Q1Z0_MOUSE	Krt90	9,57E+08	9,82E+08	1,01E+09	1,41E+09	1,03E+09	9,51E+08	1,15
3,7	Keratin, type II cytoskeletal 6B OS=Mus musculus GN=Krt6b PE=1 SV=3	Keratins	K2C6B_MOUSE (+1)	Krt6b	4,09E+08	3,98E+08	4,40E+08	6,63E+08	3,76E+08	4,47E+08	1,19
4,3	Keratin, type I cytoskeletal 14 OS=Mus musculus GN=Krt14 PE=1 SV=2	Keratins	K1C14_MOUSE	Krt14	6,35E+08	5,45E+08	6,41E+08	6,95E+08	3,74E+08	4,58E+08	0,84
4,5	Keratin 15, isoform CRA_a OS=Mus musculus GN=Krt15 PE=1 SV=1	Keratins	B1AQ77_MOUSE (+2)	Krt15	4,84E+08	2,57E+08	4,97E+08	3,96E+08	2,13E+08	2,16E+08	0,67
5	Desmoplakin OS=Mus musculus GN=Dsp PE=1 SV=1	Cell junctional proteins	DESP_MOUSE	Dsp	9,40E+07	1,33E+08	1,12E+08	1,11E+08	1,14E+08	1,23E+08	1,03
6	Leucine-rich repeat-containing protein 15 OS=Mus musculus GN=Lrrc15 PE=2 SV=1	Nail-associated proteins	LRC15_MOUSE	Lrrc15	5,10E+09	7,39E+09	5,35E+09	4,71E+09	6,05E+09	7,41E+09	1,02
7	**Cluster of Keratin-associated protein 4-16 OS=Mus musculus GN=Krtap4-16 PE=1 SV=1 (Q91W93_MOUSE)**	KRTAP	**Q91W93_MOUSE [13]**	**Krtap4-16 Cluster**	2,65E+09	1,78E+09	2,79E+09	2,13E+09	1,93E+09	1,30E+09	0,74
7,1	Predicted gene 11555 OS=Mus musculus GN=Gm11555 PE=4 SV=1	KRTAP	E9PUW3_MOUSE	Gm11555	2,15E+09	1,51E+09	2,20E+09	1,94E+09	2,05E+09	1,55E+09	0,94
7,1	Keratin-associated protein 4-16 OS=Mus musculus GN=Krtap4-16 PE=1 SV=1	KRTAP	Q91W93_MOUSE	Krtap4-16	1,31E+09	5,75E+08	1,51E+09	2,93E+08	7,59E+08	7,21E+08	0,52
7,11	Keratin-associated protein 4-9 OS=Mus musculus GN=Krtap4-9 PE=1 SV=1	KRTAP	B1AQA9_MOUSE	Krtap4-9	2,92E+09	2,02E+09	3,12E+09	2,01E+09	1,72E+09	1,21E+09	0,61
7,12	Keratin-associated protein 4-7 OS=Mus musculus GN=Krtap4-7 PE=2 SV=1	KRTAP	Q9D732_MOUSE	Krtap4-7	3,69E+09	2,64E+09	3,86E+09	3,46E+09	2,75E+09	1,77E+09	0,78
7,13	Predicted gene 11554 OS=Mus musculus GN=Gm11554 PE=4 SV=1	KRTAP	B1AQB2_MOUSE	Gm11554	2,09E+09	1,33E+09	2,28E+09	1,46E+09	1,01E+09	6,27E+08	0,54
7,2	MCG140290 OS=Mus musculus GN=Gm11595 PE=4 SV=1	KRTAP	B1AQA7_MOUSE	Gm11595	2,72E+09	1,62E+09	2,84E+09	2,03E+09	1,64E+09	8,44E+08	0,63
7,3	Keratin-associated protein 4-6 OS=Mus musculus GN=Krtap4-6 PE=1 SV=1	KRTAP	Q3V4B7_MOUSE	Krtap4-6	3,92E+09	2,55E+09	3,93E+09	2,96E+09	2,52E+09	1,48E+09	0,67
7,5	Predicted gene 11563 OS=Mus musculus GN=Gm11563 PE=4 SV=1	KRTAP	B1AQ90_MOUSE	Gm11563	2,84E+09	1,88E+09	2,85E+09	2,71E+09	2,97E+09	1,96E+09	1,01
7,6	Keratin-associated protein 4-1 OS=Mus musculus GN=Krtap4-1 PE=2 SV=1	KRTAP	Q3UUY3_MOUSE	Krtap4-1	3,35E+09	2,53E+09	3,61E+09	3,04E+09	2,36E+09	1,70E+09	0,75
7,8	Keratin-associated protein 4-2 OS=Mus musculus GN=Krtap4-2 PE=4 SV=1	KRTAP	B1AQ85_MOUSE	Krtap4-2	2,30E+09	1,62E+09	2,37E+09	2,19E+09	2,43E+09	1,68E+09	1,00
7,9	MCG140213 OS=Mus musculus GN=Gm11596 PE=4 SV=1	KRTAP	B1AQB0_MOUSE	Gm11596	1,91E+09	1,31E+09	2,11E+09	1,28E+09	1,02E+09	8,00E+08	0,58
8	Krtap2-4 protein OS=Mus musculus GN=Gm11562 PE=2 SV=1	KRTAP	A0PK51_MOUSE (+1)	Gm11562	1,20E+10	8,24E+09	8,84E+09	9,68E+09	9,14E+09	1,02E+10	1,00
9	**Cluster of Keratin, type I cuticular Ha6 OS=Mus musculus GN=Krt36 PE=1 SV=1 (KRT36_MOUSE)**	Keratins	**KRT36_MOUSE [5]**	**Krt36 Cluster**	2,06E+09	2,50E+09	2,16E+09	3,47E+09	2,01E+09	2,05E+09	1,12
9,1	Keratin, type I cuticular Ha6 OS=Mus musculus GN=Krt36 PE=1 SV=1	Keratins	KRT36_MOUSE	Krt36	3,41E+09	4,37E+09	3,61E+09	3,47E+09	3,48E+09	3,52E+09	0,92
9,3	Keratin, type I cuticular Ha2 OS=Mus musculus GN=Krt32 PE=1 SV=1	Keratins	B1ATJ5_MOUSE (+2)	Krt32	7,09E+08	6,20E+08	7,16E+08	0	5,35E+08	5,79E+08	0,54
10	Keratin-associated protein 6-5 OS=Mus musculus GN=Krtap6-5 PE=2 SV=1	KRTAP	KRA65_MOUSE (+1)	Krtap6-5	5,20E+09	4,43E+09	5,54E+09	4,65E+09	4,10E+09	4,75E+09	0,89
11	2310061N02Rik protein OS=Mus musculus GN=2310061N02Rik PE=1 SV=1	KRTAP	Q9D6S9_MOUSE	Krtatp 2310061N02	1,55E+09	1,13E+09	2,01E+09	1,49E+09	1,78E+09	1,36E+09	0,99
12	**Cluster of Actin, alpha cardiac muscle 1 OS=Mus musculus GN=Actc1 PE=1 SV=1 (ACTC_MOUSE)**	Filaments	**ACTC_MOUSE [4]**	**Actc1 Cluster**	2,72E+08	3,99E+08	2,90E+08	8,16E+08	8,30E+08	9,54E+08	2,71
12,1	Actin, alpha cardiac muscle 1 OS=Mus musculus GN=Actc1 PE=1 SV=1	Filaments	ACTC_MOUSE (+1)	Actc1	1,92E+08	2,55E+08	2,17E+08	5,81E+08	5,37E+08	6,13E+08	2,61
12,2	Actin, cytoplasmic 1 OS=Mus musculus GN=Actb PE=1 SV=1	Filaments	ACTB_MOUSE	Actb	3,12E+08	4,70E+08	3,26E+08	9,37E+08	9,79E+08	1,13E+09	2,75
12,3	Actin, cytoplasmic 2 OS=Mus musculus GN=Actg1 PE=1 SV=1	Filaments	ACTG_MOUSE	Actg1	3,12E+08	4,71E+08	3,27E+08	9,31E+08	9,74E+08	1,12E+09	2,73
13	**Cluster of Junction plakoglobin OS=Mus musculus GN=Jup PE=1 SV=3 (PLAK_MOUSE)**	Cell junctional proteins	**PLAK_MOUSE [2]**	**Jup Cluster**	1,85E+08	3,10E+08	2,42E+08	1,29E+08	2,37E+08	1,49E+08	0,70
13,1	Junction plakoglobin OS=Mus musculus GN=Jup PE=1 SV=3	Cell junctional proteins	PLAK_MOUSE	Jup	1,85E+08	3,10E+08	2,42E+08	2,39E+08	2,37E+08	2,66E+08	1,01
13,2	Catenin beta-1 OS=Mus musculus GN=Ctnnb1 PE=1 SV=1	Cell junctional proteins	CTNB1_MOUSE	Ctnnb1	0	0	0	2,04E+07	0	3,11E+07	>30,00
14	**Cluster of MCG128973 OS=Mus musculus GN=2310057N15Rik PE=2 SV=1 (Q9D6T8_MOUSE)**	KRTAP	**Q9D6T8_MOUSE [2]**	**2310057N15Rik Clu**	3,52E+09	2,65E+09	3,67E+09	3,19E+09	3,62E+09	2,81E+09	0,98
14,1	MCG128973 OS=Mus musculus GN=2310057N15Rik PE=2 SV=1	KRTAP	Q9D6T8_MOUSE	Krtap 2310057N15	3,74E+09	2,82E+09	3,88E+09	3,32E+09	3,76E+09	3,01E+09	0,97
14,2	Predicted gene 5965 OS=Mus musculus GN=Gm5965 PE=4 SV=1	KRTAP	D3Z724_MOUSE	Krtap Gm5965	3,30E+09	2,48E+09	3,46E+09	3,06E+09	3,49E+09	2,61E+09	0,99
15	Keratin-associated protein 13-1 OS=Mus musculus GN=Krtap13-1 PE=1 SV=1	KRTAP	Q8K198_MOUSE	Krtap13-1	2,56E+09	4,13E+09	2,69E+09	2,59E+09	3,43E+09	3,62E+09	1,03
16	Elongation factor 2 OS=Mus musculus GN=Eef2 PE=1 SV=2	Translation factors	EF2_MOUSE	Eef2	1,12E+08	1,36E+08	1,22E+08	2,09E+08	2,43E+08	2,72E+08	1,96
17	Elongation factor 1-alpha 1 OS=Mus musculus GN=Eef1a1 PE=1 SV=3	Translation factors	EF1A1_MOUSE	Eef1a1	3,56E+08	4,41E+08	4,14E+08	7,81E+08	8,26E+08	7,34E+08	1,93
18	Selenium-binding protein 1 OS=Mus musculus GN=Selenbp1 PE=1 SV=2	Other proteins	SBP1_MOUSE	Selenbp1	1,12E+08	1,60E+08	1,24E+08	2,39E+08	3,77E+08	3,13E+08	2,35
19	Serum albumin OS=Mus musculus GN=Alb PE=1 SV=3	Other proteins	ALBU_MOUSE	Alb	6,34E+07	9,06E+07	8,99E+07	1,01E+08	1,06E+08	1,40E+08	1,42
20	**Cluster of Histone H2B type 1-B OS=Mus musculus GN=Hist1h2bb PE=1 SV=3 (H2B1B_MOUSE)**	Histones	**H2B1B_MOUSE [10]**	**Hist1h2bb Cluster**	8,14E+08	1,14E+09	8,24E+08	7,82E+08	9,05E+08	9,32E+08	0,94
20,1	Histone H2B type 1-B OS=Mus musculus GN=Hist1h2bb PE=1 SV=3	Histones	H2B1B_MOUSE (+5)	Hist1h2bb	8,14E+08	1,14E+09	8,24E+08	7,82E+08	9,05E+08	9,32E+08	0,94
20,2	Histone H2B type 1-C/E/G OS=Mus musculus GN=Hist1h2bc PE=1 SV=3	Histones	H2B1C_MOUSE (+3)	Hist1h2bc	8,14E+08	1,14E+09	8,24E+08	7,82E+08	9,05E+08	9,32E+08	0,94
21	Keratin-associated protein 7-1 OS=Mus musculus GN=Krtap7-1 PE=3 SV=1	KRTAP	KRA71_MOUSE	Krtap7-1	6,83E+09	1,22E+10	5,73E+09	8,15E+09	7,58E+09	9,92E+09	1,04
22	Keratin, type I cytoskeletal 10 OS=Mus musculus GN=Krt10 PE=1 SV=1	Keratins	A2A513_MOUSE (+4)	Krt10	6,14E+08	5,94E+08	5,35E+08	5,38E+08	2,39E+08	3,44E+08	0,64
23	RIKEN cDNA 2310034C09 OS=Mus musculus GN=2310034C09Rik PE=1 SV=1	KRTAP	Q9D746_MOUSE	Krtap 2310034C09	1,86E+09	1,85E+09	2,03E+09	1,59E+09	1,98E+09	1,86E+09	0,95
25	Keratin-associated protein 13 OS=Mus musculus GN=Krtap13 PE=2 SV=1	KRTAP	O88375_MOUSE	Krtap13	3,45E+09	2,72E+09	3,53E+09	3,30E+09	3,64E+09	2,30E+09	0,95
26	Lysozyme g-like protein 1 OS=Mus musculus GN=Lyg1 PE=2 SV=1	Nail-associated proteins	LYG1_MOUSE	Lyg1	4,49E+08	8,16E+08	6,41E+08	6,60E+08	7,00E+08	7,18E+08	1,09
27	14-3-3 protein sigma OS=Mus musculus GN=Sfn PE=1 SV=2	Other proteins	1433S_MOUSE	Sfn	2,72E+08	3,64E+08	2,25E+08	4,96E+08	5,31E+08	6,18E+08	1,91
28	Histone H4 OS=Mus musculus GN=Hist1h4a PE=1 SV=2	Histones	H4_MOUSE	Hist1h4a	1,54E+09	1,96E+09	1,75E+09	1,84E+09	1,80E+09	1,85E+09	1,05
29	**Cluster of Tubulin alpha-1C chain OS=Mus musculus GN=Tuba1c PE=1 SV=1 (TBA1C_MOUSE)**	Filaments	**TBA1C_MOUSE [3]**	**Tuba1c Cluster**	5,77E+07	9,77E+07	7,71E+07	7,93E+07	1,02E+08	1,06E+08	1,23
29,1	Tubulin alpha-1C chain OS=Mus musculus GN=Tuba1c PE=1 SV=1	Filaments	TBA1C_MOUSE	Tuba1c	6,37E+07	1,07E+08	8,43E+07	8,64E+07	1,13E+08	1,18E+08	1,25
29,2	Tubulin alpha chain (Fragment) OS=Mus musculus GN=Tuba4a PE=1 SV=1	Filaments	A0A0A0MQA5_MOUSE (+1)	Tuba4a	5,17E+07	8,86E+07	6,98E+07	7,22E+07	9,00E+07	9,35E+07	1,22
30	Galectin OS=Mus musculus GN=Lgals7 PE=1 SV=1	Other proteins	Q9CRB1_MOUSE	Lgals7	5,05E+08	7,16E+08	6,68E+08	7,94E+08	8,31E+08	1,16E+09	1,48
31	Pyruvate kinase PKM OS=Mus musculus GN=Pkm PE=1 SV=4	Enzymes	sp|P52480|KPYM_MOUSE	Pkm	2,89E+07	4,14E+07	4,14E+07	1,53E+08	1,24E+08	1,08E+08	3,44
32	Heat shock protein beta-1 OS=Mus musculus GN=Hspb1 PE=1 SV=3	Heat shock proteins	sp|P14602|HSPB1_MOUSE	Hspb1	2,00E+08	2,62E+08	2,10E+08	5,29E+08	5,61E+08	5,92E+08	2,50
33	Plakophilin-1 OS=Mus musculus GN=Pkp1 PE=1 SV=1	Cell junctional proteins	PKP1_MOUSE	Pkp1	6,75E+07	6,36E+07	8,08E+07	7,48E+07	7,33E+07	8,44E+07	1,10
34	Isoform 2 of Cytosol aminopeptidase OS=Mus musculus GN=Lap3	Enzymes	sp|Q9CPY7-2|AMPL_MOUSE (+1)	Lap3	5,08E+07	1,16E+08	5,54E+07	1,47E+08	1,74E+08	2,32E+08	2,49
35	Keratin, type II cytoskeletal 1 OS=Mus musculus GN=Krt1 PE=1 SV=4	Keratins	K2C1_MOUSE	Krt1	2,43E+08	3,16E+08	2,31E+08	3,30E+08	2,40E+08	3,06E+08	1,11
36	Keratin-associated protein 11-1 OS=Mus musculus GN=Krtap11-1 PE=1 SV=1	KRTAP	E9Q2E9_MOUSE	Krtap11-1	1,91E+09	1,11E+09	2,06E+09	1,54E+09	1,94E+09	2,44E+08	0,73
37	**Desmoglein-4 OS=Mus musculus GN=Dsg4 PE=1 SV=1**	Cell junctional proteins	**DSG4_MOUSE**	**Dsg4**	5,74E+07	9,64E+07	6,62E+07	6,23E+07	6,06E+07	9,88E+07	1,01
39	**Cluster of Tubulin beta-4B chain OS=Mus musculus GN=Tubb4b PE=1 SV=1 (TBB4B_MOUSE)**	Filaments	**TBB4B_MOUSE [3]**	**Tubb4b Cluster**	4,70E+07	8,05E+07	7,11E+07	7,47E+07	7,76E+07	1,04E+08	1,29
39,1	Tubulin beta-4B chain OS=Mus musculus GN=Tubb4b PE=1 SV=1	Filaments	TBB4B_MOUSE	Tubb4b	4,62E+07	8,20E+07	7,15E+07	7,63E+07	7,84E+07	1,07E+08	1,31
39,2	Tubulin beta-2A chain OS=Mus musculus GN=Tubb2a PE=1 SV=1	Filaments	TBB2A_MOUSE	Tubb2a	4,49E+07	7,48E+07	6,99E+07	7,03E+07	7,21E+07	1,04E+08	1,30
39,3	Tubulin beta-5 chain OS=Mus musculus GN=Tubb5 PE=1 SV=1	Filaments	TBB5_MOUSE	Tubb5	5,00E+07	8,48E+07	7,20E+07	7,77E+07	8,22E+07	9,96E+07	1,25
40	Fatty acid-binding protein, epidermal OS=Mus musculus GN=Fabp5 PE=1 SV=3	Other proteins	FABP5_MOUSE	Fabp5	2,78E+08	6,14E+08	2,73E+08	7,10E+08	7,85E+08	1,00E+09	2,14
41	**Cluster of 14-3-3 protein zeta/delta OS=Mus musculus GN=Ywhaz PE=1 SV=1 (1433Z_MOUSE)**	Other proteins	**1433Z_MOUSE [7]**	**Ywhaz Cluster**	8,10E+07	1,24E+08	1,01E+08	1,70E+08	1,78E+08	1,82E+08	1,74
41,1	14-3-3 protein zeta/delta OS=Mus musculus GN=Ywhaz PE=1 SV=1	Other proteins	1433Z_MOUSE	Ywhaz	1,16E+08	1,68E+08	1,49E+08	2,45E+08	2,62E+08	2,55E+08	1,76
41,2	14-3-3 protein theta (Fragment) OS=Mus musculus GN=Ywhaq PE=1 SV=1	Other proteins	F6VW30_MOUSE (+3)	Ywhaq	5,13E+07	9,20E+07	6,60E+07	1,13E+08	1,17E+08	1,27E+08	1,71
41,3	Isoform Short of 14-3-3 protein beta/alpha OS=Mus musculus GN=Ywhab	Other proteins	sp|Q9CQV8-2|1433B_MOUSE (+	Ywhab	7,54E+07	1,11E+08	8,65E+07	1,54E+08	1,54E+08	1,62E+08	1,72
42	Alpha-actinin-4 OS=Mus musculus GN=Actn4 PE=1 SV=1	Filaments	ACTN4_MOUSE	Actn4	3445000	7972700	7446800	8,14E+07	9,26E+07	7,17E+07	13,02
44	**Cluster of Eukaryotic initiation factor 4A-I OS=Mus musculus GN=Eif4a1 PE=1 SV=1 (IF4A1_MOUSE)**	Translation factors	**IF4A1_MOUSE [4]**	**Eif4a1 Cluster**	3,90E+07	5,81E+07	4,14E+07	6,75E+07	7,68E+07	7,26E+07	1,57
44,1	Eukaryotic initiation factor 4A-I OS=Mus musculus GN=Eif4a1 PE=1 SV=1	Translation factors	IF4A1_MOUSE	Eif4a1	5,17E+07	8,86E+07	5,66E+07	1,01E+08	1,13E+08	1,08E+08	1,64
44,2	Eukaryotic initiation factor 4A-II OS=Mus musculus GN=Eif4a2 PE=1 SV=1	Translation factors	Q8BTU6_MOUSE (+2)	Eif4a2	2,64E+07	2,75E+07	2,62E+07	3,37E+07	4,10E+07	3,69E+07	1,39
45	**Cluster of Histone H3.2 OS=Mus musculus GN=Hist2h3c1 PE=1 SV=1 (A0A1W2P768_MOUSE)**	Histones	**A0A1W2P768_MOUSE [5]**	**Hist2h3c1 Cluster**	9,83E+08	1,08E+09	1,02E+09	1,14E+09	1,03E+09	1,02E+09	1,03
45,1	Histone H3.2 OS=Mus musculus GN=Hist2h3c1 PE=1 SV=1	Histones	A0A1W2P768_MOUSE (+1)	Hist2h3c1	1,13E+09	8,59E+08	9,01E+08	1,28E+09	1,43E+09	1,33E+09	1,40
45,2	Histone H3.3 OS=Mus musculus GN=H3f3a PE=1 SV=2	Histones	H33_MOUSE (+1)	H3f3a	1,19E+09	1,64E+09	1,38E+09	1,35E+09	8,70E+08	1,37E+09	0,85
45,3	Histone H3.1 OS=Mus musculus GN=Hist1h3a PE=1 SV=2	Histones	H31_MOUSE	Hist1h3a	6,25E+08	7,41E+08	7,79E+08	7,79E+08	7,79E+08	3,78E+08	0,90
46	ATP synthase subunit beta, mitochondrial OS=Mus musculus GN=Atp5b PE=1 SV=2	Enzymes	ATPB_MOUSE	Atp5b	4,15E+07	5,74E+07	4,99E+07	8,00E+07	9,02E+07	1,01E+08	1,82
47	Kallikrein-14 OS=Mus musculus GN=Klk14 PE=2 SV=1	Enzymes	KLK14_MOUSE	Klk14	1,77E+08	2,47E+08	2,27E+08	1,96E+08	2,52E+08	2,19E+08	1,02
48	Keratinocyte proline-rich protein OS=Mus musculus GN=Kprp PE=2 SV=1	Other proteins	KPRP_MOUSE	Kprp	3,64E+07	3,32E+07	3,80E+07	5,71E+07	5,55E+07	3,45E+07	1,37
49	Carboxypeptidase M OS=Mus musculus GN=Cpm PE=1 SV=2	Enzymes	sp|Q80V42|CBPM_MOUSE	Cpm	5,75E+07	7,49E+07	6,21E+07	7,40E+07	7,93E+07	8,24E+07	1,21
50	**Cluster of Histone H2A type 2-A OS=Mus musculus GN=Hist2h2aa1 PE=1 SV=3 (H2A2A_MOUSE)**	Histones	**H2A2A_MOUSE [3]**	**Hist2h2aa1 Cluster**	8,19E+08	2,14E+09	1,02E+09	1,35E+09	1,54E+09	1,38E+09	1,08
50,1	Histone H2A type 2-A OS=Mus musculus GN=Hist2h2aa1 PE=1 SV=3	Histones	H2A2A_MOUSE (+1)	Hist2h2aa1	9,70E+08	1,96E+09	1,08E+09	1,41E+09	1,72E+09	1,17E+09	1,07
50,2	Histone H2AX OS=Mus musculus GN=H2afx PE=1 SV=2	Histones	H2AX_MOUSE	H2afx	6,67E+08	2,32E+09	9,54E+08	1,28E+09	1,36E+09	1,59E+09	1,08
51	Polyadenylate-binding protein 1 OS=Mus musculus GN=Pabpc1 PE=1 SV=2	Other proteins	PABP1_MOUSE	Pabpc1	3,23E+07	5,22E+07	3,30E+07	6,26E+07	7,53E+07	7,90E+07	1,85
52	Glyceraldehyde-3-phosphate dehydrogenase OS=Mus musculus GN=Gapdh PE=1 SV=1	Enzymes	A0A0A0MQF6_MOUSE (+1)	Gapdh	6,95E+07	1,43E+08	9,49E+07	2,63E+08	2,42E+08	3,02E+08	2,62
53	Dual specificity protein phosphatase 14 OS=Mus musculus GN=Dusp14 PE=2 SV=2	Enzymes	DUS14_MOUSE	Dusp14	1,54E+08	2,59E+08	1,44E+08	1,60E+08	1,54E+08	2,08E+08	0,94
54	Peptidyl-prolyl cis-trans isomerase A OS=Mus musculus GN=Ppia PE=1 SV=2	Enzymes	PPIA_MOUSE	Ppia	9,91E+07	1,96E+08	9,20E+07	1,62E+08	1,55E+08	1,81E+08	1,29
55	40S ribosomal protein S4, X isoform OS=Mus musculus GN=Rps4x PE=1 SV=2	Ribosome	RS4X_MOUSE	Rps4x	5,06E+07	7,04E+07	5,90E+07	1,11E+08	1,31E+08	1,04E+08	1,92
56	Transitional endoplasmic reticulum ATPase OS=Mus musculus GN=Vcp PE=1 SV=4	Enzymes	TERA_MOUSE	Vcp	1,39E+07	2,41E+07	1,58E+07	5,05E+07	5,76E+07	5,96E+07	3,11
57	Alpha-enolase OS=Mus musculus GN=Eno1 PE=1 SV=3	Enzymes	ENOA_MOUSE	Eno1	4,52E+07	7,10E+07	4,19E+07	1,06E+08	1,12E+08	1,16E+08	2,11
58	Protein POF1B OS=Mus musculus GN=Pof1b PE=2 SV=3	Other proteins	POF1B_MOUSE	Pof1b	3,61E+07	6,38E+07	4,31E+07	5,37E+07	5,72E+07	6,26E+07	1,21
59	60S ribosomal protein L4 OS=Mus musculus GN=Rpl4 PE=1 SV=3	Ribosome	RL4_MOUSE	Rpl4	3,18E+07	4,78E+07	3,96E+07	6,77E+07	7,24E+07	7,30E+07	1,79
60	Keratin-associated protein 1-5 OS=Mus musculus GN=Krtap1-5 PE=2 SV=1	KRTAP	A2A590_MOUSE	Krtap1-5	5,86E+09	3,50E+09	6,19E+09	3,85E+09	2,04E+09	3,33E+09	0,59
61	**Cluster of Keratin-associated protein 1-4 OS=Mus musculus GN=Krtap1-4 PE=2 SV=1 (Q3V2D6_MOUSE)**	KRTAP	**Q3V2D6_MOUSE [2]**	**Krtap1-4 Cluster**	5,85E+09	3,67E+09	6,19E+09	3,57E+09	1,65E+09	3,36E+09	0,55
61,1	Keratin-associated protein 1-4 OS=Mus musculus GN=Krtap1-4 PE=2 SV=1	KRTAP	Q3V2D6_MOUSE	Krtap1-4	4,68E+09	2,94E+09	4,95E+09	2,85E+09	1,32E+09	2,69E+09	0,55
61,2	Keratin-associated protein 9-3 OS=Mus musculus GN=Krtap9-3 PE=2 SV=1	KRTAP	KRA93_MOUSE	Krtap9-3	7,02E+09	4,41E+09	7,43E+09	4,28E+09	1,99E+09	4,03E+09	0,55
62	**Cluster of Desmoglein-1-alpha OS=Mus musculus GN=Dsg1a PE=2 SV=2 (DSG1A_MOUSE)**	Cell junctional proteins	**DSG1A_MOUSE [2]**	**Dsg1a Cluster**	2,39E+07	3,48E+07	3,01E+07	2,89E+07	2,22E+07	2,33E+07	0,84
62,1	Desmoglein-1-alpha OS=Mus musculus GN=Dsg1a PE=2 SV=2	Cell junctional proteins	DSG1A_MOUSE	Dsg1a	2,49E+07	3,74E+07	3,10E+07	3,10E+07	2,33E+07	2,27E+07	0,83
62,2	Desmoglein-1-beta OS=Mus musculus GN=Dsg1b PE=1 SV=1	Cell junctional proteins	DSG1B_MOUSE	Dsg1b	2,30E+07	3,23E+07	2,92E+07	2,67E+07	2,11E+07	2,38E+07	0,85
63	L-lactate dehydrogenase OS=Mus musculus GN=Ldha PE=1 SV=1	Enzymes	A0A1B0GSX0_MOUSE (+2)	Ldha	4,41E+07	6,59E+07	5,80E+07	1,46E+08	1,52E+08	1,53E+08	2,68
64	40S ribosomal protein S2 OS=Mus musculus GN=Rps2 PE=1 SV=3	Ribosome	RS2_MOUSE	Rps2	3,99E+07	4,43E+07	5,13E+07	8,45E+07	8,59E+07	8,81E+07	1,91
65	**Cluster of Heat shock cognate 71 kDa protein OS=Mus musculus GN=Hspa8 PE=1 SV=1 (HSP7C_MOUSE)**	Heat shock proteins	**HSP7C_MOUSE [2]**	**Hspa8 Cluster**	2,18E+07	3,01E+07	2,51E+07	5,74E+07	5,32E+07	5,31E+07	2,13
65,1	Heat shock cognate 71 kDa protein OS=Mus musculus GN=Hspa8 PE=1 SV=1	Heat shock proteins	HSP7C_MOUSE	Hspa8	2,73E+07	3,97E+07	3,10E+07	7,24E+07	6,62E+07	6,62E+07	2,09
65,2	Heat shock-related 70 kDa protein 2 OS=Mus musculus GN=Hspa2 PE=1 SV=2	Heat shock proteins	HSP72_MOUSE	Hspa2	1,62E+07	2,06E+07	1,92E+07	4,24E+07	4,02E+07	4,00E+07	2,19
66	Prelamin-A/C OS=Mus musculus GN=Lmna PE=1 SV=2	Other proteins	sp|P48678|LMNA_MOUSE	Lmna	2,02E+07	3,29E+07	2,00E+07	3,70E+07	3,25E+07	2,88E+07	1,34
67	40S ribosomal protein SA OS=Mus musculus GN=Rpsa PE=1 SV=4	Ribosome	RSSA_MOUSE	Rpsa	6,38E+07	7,85E+07	7,50E+07	1,32E+08	1,69E+08	1,53E+08	2,09
68	Protein S100-A3 OS=Mus musculus GN=S100a3 PE=2 SV=1	Other proteins	S10A3_MOUSE	S100a3	4,00E+08	9,10E+08	4,47E+08	6,93E+08	9,76E+08	6,12E+08	1,30
69	NADP-dependent malic enzyme OS=Mus musculus GN=Me1 PE=1 SV=2	Enzymes	MAOX_MOUSE	Me1	1,75E+07	3,04E+07	2,23E+07	6,15E+07	7,77E+07	6,19E+07	2,87
70	Receptor of activated protein C kinase 1 OS=Mus musculus GN=Rack1 PE=1 SV=3	Enzymes	RACK1_MOUSE	Rack1	4,24E+07	3,64E+07	4,73E+07	8,33E+07	8,85E+07	5,93E+07	1,83
71	60S ribosomal protein L6 OS=Mus musculus GN=Rpl6 PE=1 SV=3	Ribosome	RL6_MOUSE	Rpl6	4,84E+07	3,78E+07	4,89E+07	8,58E+07	8,48E+07	7,86E+07	1,84
72	Proliferation-associated 2G4 OS=Mus musculus GN=Pa2g4 PE=1 SV=1	Other proteins	Q3TGU7_MOUSE (+1)	Pa2g4	3,07E+07	3,73E+07	3,09E+07	6,58E+07	8,36E+07	8,26E+07	2,34
73	Clathrin heavy chain 1 OS=Mus musculus GN=Cltc PE=1 SV=3	Other proteins	CLH1_MOUSE (+1)	Cltc	1,05E+07	1,27E+07	1,02E+07	1,51E+07	1,94E+07	2,11E+07	1,67
74	Serpin B5 OS=Mus musculus GN=Serpinb5 PE=1 SV=1	Other proteins	SPB5_MOUSE	Serpinb5	2,99E+07	4,18E+07	3,59E+07	6,77E+07	8,14E+07	9,06E+07	2,23
75	Annexin A2 OS=Mus musculus GN=Anxa2 PE=1 SV=2	Other proteins	ANXA2_MOUSE	Anxa2	2,02E+07	3,25E+07	2,76E+07	4,78E+07	3,79E+07	3,88E+07	1,55
76	Elongation factor 1-gamma OS=Mus musculus GN=Eef1g PE=1 SV=3	Translation factors	EF1G_MOUSE	Eef1g	1,90E+07	2,65E+07	3,36E+07	7,00E+07	6,89E+07	6,39E+07	2,57
77	Beta-globin OS=Mus musculus GN=Hbbt1 PE=1 SV=1	Other proteins	A8DUK4_MOUSE	Hbbt1	9,43E+07	1,33E+08	1,27E+08	1,39E+08	1,57E+08	1,86E+08	1,36
78	60S ribosomal protein L3 OS=Mus musculus GN=Rpl3 PE=1 SV=3	Ribosome	RL3_MOUSE	Rpl3	3,24E+07	8,20E+07	4,23E+07	9,68E+07	1,51E+08	1,22E+08	2,36
79	40S ribosomal protein S3 OS=Mus musculus GN=Rps3 PE=1 SV=1	Ribosome	RS3_MOUSE	Rps3	5,89E+07	6,85E+07	6,39E+07	8,85E+07	1,17E+08	1,10E+08	1,65
80	60S acidic ribosomal protein P0 OS=Mus musculus GN=Rplp0 PE=1 SV=3	Ribosome	RLA0_MOUSE	Rplp0	4,59E+07	5,62E+07	4,64E+07	1,01E+08	1,00E+08	1,15E+08	2,13
82	40S ribosomal protein S7 OS=Mus musculus GN=Rps7 PE=2 SV=1	Ribosome	RS7_MOUSE	Rps7	6,37E+07	9,68E+07	7,12E+07	1,48E+08	1,55E+08	1,96E+08	2,15
83	Kallikrein-related-peptidase 15 OS=Mus musculus GN=Klk15 PE=2 SV=1	Enzymes	Q8CGR4_MOUSE	Klk15	9,24E+07	9,09E+07	1,27E+08	1,17E+08	1,17E+08	1,06E+08	1,10
84	Protein-glutamine gamma-glutamyltransferase E OS=Mus musculus GN=Tgm3 PE=1 SV=2	Enzymes	TGM3_MOUSE	Tgm3	1,63E+07	3,31E+07	1,94E+07	3,36E+07	2,65E+07	4,69E+07	1,56
86	40S ribosomal protein S3a OS=Mus musculus GN=Rps3a PE=1 SV=3	Ribosome	RS3A_MOUSE	Rps3a	3,49E+07	4,09E+07	3,79E+07	7,37E+07	7,65E+07	8,03E+07	2,03
87	**Cluster of Heat shock protein HSP 90-beta OS=Mus musculus GN=Hsp90ab1 PE=1 SV=3 (HS90B_MOUSE)**	Heat shock proteins	**HS90B_MOUSE [2]**	**Hsp90ab1 Cluster**	1,01E+07	1,88E+07	1,71E+07	2,27E+07	3,44E+07	3,38E+07	1,97
87,1	Heat shock protein HSP 90-beta OS=Mus musculus GN=Hsp90ab1 PE=1 SV=3	Heat shock proteins	HS90B_MOUSE	Hsp90ab1	1,77E+07	3,13E+07	2,74E+07	3,86E+07	5,70E+07	5,30E+07	1,95
87,2	Heat shock protein HSP 90-alpha OS=Mus musculus GN=Hsp90aa1 PE=1 SV=4	Heat shock proteins	HS90A_MOUSE	Hsp90aa1	2606200	6302400	6799200	6785800	1,18E+07	1,45E+07	2,11
88	Galectin-3 OS=Mus musculus GN=Lgals3 PE=1 SV=3	Other proteins	LEG3_MOUSE (+1)	Lgals3	2,07E+08	2,95E+08	1,81E+08	2,80E+08	3,15E+08	2,72E+08	1,27
89	Keratin-associated protein 3-1 OS=Mus musculus GN=Krtap3-1 PE=3 SV=1	KRTAP	KRA31_MOUSE (+1)	Krtap3-1	2,44E+09	3,50E+09	2,24E+09	2,74E+09	2,59E+09	2,66E+09	0,98
91	40S ribosomal protein S8 OS=Mus musculus GN=Rps8 PE=1 SV=2	Ribosome	RS8_MOUSE	Rps8	6,53E+07	1,13E+08	6,92E+07	1,04E+08	1,26E+08	1,39E+08	1,49
92	Phosphoglycerate mutase 1 OS=Mus musculus GN=Pgam1 PE=1 SV=3	Enzymes	PGAM1_MOUSE	Pgam1	5,26E+07	1,08E+08	7,04E+07	1,47E+08	1,72E+08	1,83E+08	2,18
93	Peroxiredoxin-2 OS=Mus musculus GN=Prdx2 PE=1 SV=3	Other proteins	PRDX2_MOUSE	Prdx2	3,85E+07	2,75E+07	3,03E+07	1,11E+08	1,02E+08	1,10E+08	3,35
94	60S ribosomal protein L7 OS=Mus musculus GN=Rpl7 PE=1 SV=2	Ribosome	RL7_MOUSE	Rpl7	9,62E+07	1,44E+08	1,09E+08	1,86E+08	2,29E+08	2,33E+08	1,86
95	Ribosomal protein L15 OS=Mus musculus GN=Gm10020 PE=3 SV=1	Ribosome	E9QAZ2_MOUSE (+1)	Rpl15	5,01E+07	4,30E+07	6,61E+07	1,09E+08	1,17E+08	9,86E+07	2,03
96	Isoform 2 of Plakophilin-3 OS=Mus musculus GN=Pkp3	Cell junctional proteins	sp|Q9QY23-2|PKP3_MOUSE (+1)	Pkp3	1,18E+07	2,36E+07	1,49E+07	1,45E+07	1,67E+07	1,78E+07	0,97
97	**Cluster of Histone H1.4 OS=Mus musculus GN=Hist1h1e PE=1 SV=2 (H14_MOUSE)**	Histones	**H14_MOUSE [4]**	**Hist1h1e Cluster**	5,71E+07	5,04E+07	5,72E+07	7,14E+07	7,13E+07	4,16E+07	1,12
97,1	Histone H1.4 OS=Mus musculus GN=Hist1h1e PE=1 SV=2	Histones	H14_MOUSE	Hist1h1e	5,67E+07	5,74E+07	6,18E+07	7,80E+07	7,28E+07	3,99E+07	1,08
97,2	Histone H1.1 OS=Mus musculus GN=Hist1h1a PE=1 SV=2	Histones	H11_MOUSE	Hist1h1a	4,51E+07	2,18E+07	3,49E+07	3,93E+07	4,41E+07	0	0,82
97,3	Histone H1.2 OS=Mus musculus GN=Hist1h1c PE=1 SV=2	Histones	H12_MOUSE	Hist1h1c	6,60E+07	6,39E+07	6,89E+07	8,77E+07	8,79E+07	4,44E+07	1,11
97,4	Histone H1.3 OS=Mus musculus GN=Hist1h1d PE=1 SV=2	Histones	H13_MOUSE	Hist1h1d	6,05E+07	5,85E+07	6,31E+07	8,04E+07	8,06E+07	4,07E+07	1,11
98	AHNAK nucleoprotein (desmoyokin) OS=Mus musculus GN=Ahnak PE=1 SV=1	Other proteins	E9Q616_MOUSE	Ahnak	3282800	5190100	5867800	3622500	7327000	7298900	1,27
99	40S ribosomal protein S9 OS=Mus musculus GN=Rps9 PE=1 SV=3	Ribosome	RS9_MOUSE	Rps9	5,35E+07	3,01E+07	5,32E+07	1,09E+08	1,11E+08	5,60E+07	2,02
100	Protein disulfide-isomerase OS=Mus musculus GN=P4hb PE=1 SV=2	Enzymes	PDIA1_MOUSE	P4hb	2503900	9773100	6559000	6,20E+07	6,49E+07	6,49E+07	10,18
101	Periplakin OS=Mus musculus GN=Ppl PE=1 SV=1	Cell junctional proteins	G5E898_MOUSE (+1)	Ppl	5341200	5007300	4545600	4793400	6188800	4101800	1,01
102	RalA-binding protein 1 OS=Mus musculus GN=Ralbp1 PE=1 SV=4	Other proteins	RBP1_MOUSE	Ralbp1	2943200	6106400	6996000	4064800	1,68E+07	7785200	1,79
103	60S ribosomal protein L7a OS=Mus musculus GN=Rpl7a PE=1 SV=2	Ribosome	RL7A_MOUSE	Rpl7a	5,21E+07	5,43E+07	4,90E+07	1,14E+08	1,35E+08	8,90E+07	2,18
104	Epiplakin OS=Mus musculus GN=Eppk1 PE=1 SV=2	Cell junctional proteins	EPIPL_MOUSE	Eppk1	4678500	7816200	6859800	1,02E+07	1,15E+07	1,25E+07	1,77
105	40S ribosomal protein S10 OS=Mus musculus GN=Rps10 PE=1 SV=1	Ribosome	Q3UW83_MOUSE (+1)	Rps10	2,40E+07	4,47E+07	2,52E+07	5,12E+07	6,30E+07	6,78E+07	1,94
106	60S ribosomal protein L9 OS=Mus musculus GN=Rpl9 PE=2 SV=2	Ribosome	RL9_MOUSE	Rpl9	4,39E+07	7,77E+07	4,30E+07	1,16E+08	1,23E+08	1,09E+08	2,12
107	Cytoplasmic dynein 1 heavy chain 1 OS=Mus musculus GN=Dync1h1 PE=1 SV=2	Filaments	DYHC1_MOUSE	Dync1h1	1525100	3119000	2072800	3991600	4352000	4683300	1,94
108	Transketolase OS=Mus musculus GN=Tkt PE=1 SV=1	Enzymes	TKT_MOUSE	Tkt	1,96E+07	2,23E+07	1,58E+07	2,71E+07	4,50E+07	4,40E+07	2,01
109	ATP synthase subunit alpha, mitochondrial OS=Mus musculus GN=Atp5a1 PE=1 SV=1	Enzymes	ATPA_MOUSE	Atp5a1	1,14E+07	2,70E+07	1,82E+07	3,61E+07	4,27E+07	3,67E+07	2,04

#	Identified proteins	Protein group	Accession number	Symbol	WT1	WT2	WT3	KO1	KO2	KO3	Ratio KO/WT
110	Keratin, type I cytoskeletal 24 OS=Mus musculus GN=Krt24 PE=2 SV=2	Keratins	K1C24_MOUSE	Krt24	4,90E+08	2,04E+08	5,20E+08	3,75E+08	2,07E+08	1,64E+08	0,61
111	Phosphoglycerate kinase 1 OS=Mus musculus GN=Pgk1 PE=1 SV=4	Enzymes	PGK1_MOUSE	Pgk1	7178700	2,36E+07	9754600	3,36E+07	3,41E+07	4,78E+07	2,85
112	RIKEN cDNA 2310079G19 gene OS=Mus musculus GN=2310079G19Rik PE=1 SV=1	KRTAP	Q9D6L6_MOUSE	Krtap 2310079G19	2,84E+08	1,81E+08	2,58E+08	1,68E+08	1,69E+08	3,23E+08	0,91
114	Annexin A1 OS=Mus musculus GN=Anxa1 PE=1 SV=2	Other proteins	ANXA1_MOUSE	Anxa1	1,61E+07	2,17E+07	2,18E+07	3,44E+07	2,62E+07	3,57E+07	1,61
115	40S ribosomal protein S16 OS=Mus musculus GN=Rps16 PE=1 SV=4	Ribosome	RS16_MOUSE	Rps16	3,19E+07	4,13E+07	3,47E+07	7,95E+07	7,98E+07	6,73E+07	2,10
116	40S ribosomal protein S15a OS=Mus musculus GN=Rps15a PE=1 SV=2	Ribosome	RS15A_MOUSE	Rps15a	3,82E+07	5,24E+07	3,92E+07	6,99E+07	8,14E+07	8,88E+07	1,85
117	78 kDa glucose-regulated protein OS=Mus musculus GN=Hspa5 PE=1 SV=3	Heat shock proteins	GRP78_MOUSE	Hspa5	9114500	1,51E+07	1,11E+07	4,81E+07	4,35E+07	4,84E+07	3,96
**118**	**Cluster of Predicted gene 11568 OS=Mus musculus GN=Gm11568 PE=4 SV=1 (A2A4M2_MOUSE)**	KRTAP	**A2A4M2_MOUSE [2]**	**Krtap Gm11568 Clu**	2,76E+08	1,12E+08	1,86E+08	1,90E+08	2,28E+08	1,28E+08	0,95
118,1	Predicted gene 11568 OS=Mus musculus GN=Gm11568 PE=4 SV=1	KRTAP	A2A4M2_MOUSE	Krtap Gm11568	3,31E+08	1,36E+08	2,38E+08	2,29E+08	2,73E+08	1,56E+08	0,93
118,2	Keratin-associated protein 9-1 OS=Mus musculus GN=Krtap9-1 PE=2 SV=1	KRTAP	Q64526_MOUSE	Krtap9-1	2,22E+08	8,83E+07	1,33E+08	1,52E+08	1,83E+08	1,01E+08	0,98
119	Protein-glutamine gamma-glutamyltransferase K OS=Mus musculus GN=Tgm1 PE=1 SV=1	Enzymes	A0A0R4J293_MOUSE (+1)	Tgm1	1,41E+07	2,19E+07	1,30E+07	1,18E+07	1,07E+07	1,76E+07	0,82
120	Eukaryotic translation initiation factor 3 subunit A OS=Mus musculus GN=Eif3a PE=1 SV=5	Translation factors	EIF3A_MOUSE	Eif3a	2624100	3176700	2347900	1,12E+07	7814300	8517000	3,38
**121**	**Cluster of Nucleoside diphosphate kinase OS=Mus musculus GN=Gm20390 PE=3 SV=1 (E9PZF0_MOUSE)**	Enzymes	**E9PZF0_MOUSE [2]**	**NDK Gm20390 Clus**	1,80E+07	2,33E+07	2,06E+07	5,35E+07	6,15E+07	7,90E+07	3,13
121,1	Nucleoside diphosphate kinase OS=Mus musculus GN=Gm20390 PE=3 SV=1	Enzymes	E9PZF0_MOUSE	NDK Gm20390	1,55E+07	2,15E+07	1,76E+07	5,34E+07	5,98E+07	7,24E+07	3,40
121,2	Nucleoside diphosphate kinase A OS=Mus musculus GN=Nme1 PE=1 SV=1	Enzymes	NDKA_MOUSE	Nme1	2,05E+07	2,51E+07	2,37E+07	5,35E+07	6,33E+07	8,57E+07	2,92
**122**	**Cluster of ADP/ATP translocase 2 OS=Mus musculus GN=Slc25a5 PE=1 SV=3 (ADT2_MOUSE)**	Enzymes	**ADT2_MOUSE [2]**	**Slc25a5 Cluster**	1,68E+07	2,24E+07	2,05E+07	2,98E+07	2,89E+07	2,58E+07	1,42
122,1	ADP/ATP translocase 2 OS=Mus musculus GN=Slc25a5 PE=1 SV=3	Enzymes	ADT2_MOUSE	Slc25a5	1,97E+07	2,62E+07	2,46E+07	3,51E+07	3,20E+07	3,22E+07	1,41
122,2	ADP/ATP translocase 1 OS=Mus musculus GN=Slc25a4 PE=1 SV=4	Enzymes	ADT1_MOUSE	Slc25a4	1,38E+07	1,87E+07	1,63E+07	2,45E+07	2,57E+07	1,94E+07	1,43
123	60S acidic ribosomal protein P2 OS=Mus musculus GN=Rplp2 PE=1 SV=3	Ribosome	RLA2_MOUSE	Rplp2	9,25E+07	1,32E+08	7,36E+07	1,45E+08	1,52E+08	1,81E+08	1,60
**124**	**Cluster of ADP-ribosylation factor 1 OS=Mus musculus GN=Arf1 PE=1 SV=2 (ARF1_MOUSE)**	Other proteins	**ARF1_MOUSE [3]**	**Arf1 Cluster**	1,63E+07	2,94E+07	1,83E+07	4,58E+07	6,15E+07	5,07E+07	2,47
124,1	ADP-ribosylation factor 1 OS=Mus musculus GN=Arf1 PE=1 SV=2	Other proteins	ARF1_MOUSE (+1)	Arf1	2,04E+07	3,19E+07	2,04E+07	5,36E+07	6,85E+07	5,39E+07	2,42
124,2	ADP-ribosylation factor 4 OS=Mus musculus GN=Arf4 PE=1 SV=2	Other proteins	ARF4_MOUSE	Arf4	1,22E+07	2,68E+07	1,63E+07	3,80E+07	5,44E+07	4,75E+07	2,53
125	Eukaryotic translation initiation factor 5A-1 OS=Mus musculus GN=Eif5a PE=1 SV=2	Translation factors	IF5A1_MOUSE	Eif5a	1,08E+08	1,53E+08	1,43E+08	1,46E+08	2,16E+08	2,37E+08	1,48
126	Triosephosphate isomerase OS=Mus musculus GN=Tpi1 PE=1 SV=4	Enzymes	TPIS_MOUSE	Tpi1	9953200	2,03E+07	1,01E+07	3,46E+07	4,02E+07	4,75E+07	3,03
128	Malate dehydrogenase, mitochondrial OS=Mus musculus GN=Mdh2 PE=1 SV=3	Enzymes	MDHM_MOUSE	Mdh2	1,20E+07	2,89E+07	1,72E+07	3,53E+07	3,75E+07	4,73E+07	2,07
129	Transmembrane protease serine OS=Mus musculus GN=Tmprss11c PE=3 SV=1	Enzymes	A0A0G2JET7_MOUSE (+1)	Tmprss11c	2,66E+07	3,40E+07	2,98E+07	1,97E+07	2,45E+07	2,86E+07	0,80
130	60S ribosomal protein L23 OS=Mus musculus GN=Rpl23 PE=1 SV=1	Ribosome	RL23_MOUSE	Rpl23	3,52E+07	4,58E+07	3,46E+07	7,59E+07	7,64E+07	8,71E+07	2,07
131	60S ribosomal protein L18 OS=Mus musculus GN=Rpl18 PE=1 SV=3	Ribosome	RL18_MOUSE	Rpl18	6,34E+07	5,65E+07	6,45E+07	1,20E+08	9,25E+07	1,05E+08	1,72
132	Prolyl endopeptidase OS=Mus musculus GN=Prep PE=1 SV=1	Enzymes	PPCE_MOUSE	Prep	1494700	806090	3735000	1,11E+07	2,21E+07	1,56E+07	8,09
133	Peroxiredoxin-1 OS=Mus musculus GN=Prdx1 PE=1 SV=1	Other proteins	PRDX1_MOUSE	Prdx1	2,19E+07	4,51E+07	3,27E+07	7,11E+07	7,78E+07	8,61E+07	2,36
134	60S ribosomal protein L12 OS=Mus musculus GN=Rpl12 PE=1 SV=2	Ribosome	RL12_MOUSE	Rpl12	4,74E+07	3,57E+07	4,69E+07	9,43E+07	1,02E+08	1,09E+08	2,34
135	Keratin-associated protein 6-2 OS=Mus musculus GN=Krtap6-2 PE=4 SV=1	KRTAP	A0A087WP75_MOUSE (+1)	Krtap6-2	3,75E+09	1,45E+09	3,79E+09	4,04E+09	3,91E+09	1,37E+09	1,04
136	Fructose-bisphosphate aldolase A OS=Mus musculus GN=Aldoa PE=1 SV=2	Enzymes	ALDOA_MOUSE	Aldoa	9912700	5374900	8412700	3,84E+07	3,49E+07	2,27E+07	4,05
137	Crystallin beta-gamma domain-containing 1 OS=Mus musculus GN=Crybg1 PE=1 SV=1	Other proteins	A0A0G2JG52_MOUSE (+1)	Crybg1	3773000	9849800	4734500	5578200	4522700	6520400	0,91
138	Profilin-1 OS=Mus musculus GN=Pfn1 PE=1 SV=2	Other proteins	PROF1_MOUSE	Pfn1	2,52E+07	2,80E+07	1,96E+07	5,35E+07	5,40E+07	4,44E+07	2,09
139	Alpha globin 1 OS=Mus musculus GN=Hba-a2 PE=1 SV=1	Other proteins	Q91VB8_MOUSE	Hba-a2	1,01E+08	1,37E+08	1,46E+08	1,37E+08	1,70E+08	2,25E+08	1,39
140	40S ribosomal protein S19 OS=Mus musculus GN=Rps19 PE=1 SV=3	Ribosome	RS19_MOUSE	Rps19	1,90E+07	2,09E+07	2,07E+07	4,47E+07	4,34E+07	3,35E+07	2,01
141	MCG120169 OS=Mus musculus GN=2310050C09Rik PE=1 SV=1	Other proteins	G5E8Z3_MOUSE	C1orf68/LCE	1,12E+08	1,01E+08	1,07E+08	1,37E+08	1,46E+08	8,05E+07	1,13
142	Predicted gene 10260 OS=Mus musculus GN=Gm10260 PE=3 SV=2	Ribosome	F6YVP7_MOUSE (+2)	Rps18	4,43E+07	4,99E+07	5,49E+07	9,06E+07	8,82E+07	7,53E+07	1,70
143	60S ribosomal protein L13 OS=Mus musculus GN=Rpl13 PE=1 SV=3	Ribosome	RL13_MOUSE	Rpl13	1,70E+07	2,49E+07	1,54E+07	8,19E+07	5,96E+07	3,04E+07	3,01
144	40S ribosomal protein S6 OS=Mus musculus GN=Rps6 PE=1 SV=1	Ribosome	RS6_MOUSE	Rps6	3,00E+07	5,69E+07	2,83E+07	6,55E+07	7,07E+07	8,76E+07	1,94
145	40S ribosomal protein S5 (Fragment) OS=Mus musculus GN=Rps5 PE=1 SV=1	Ribosome	D3YYM6_MOUSE (+2)	Rps5	5,92E+07	1,77E+08	8,16E+07	1,58E+08	2,48E+08	2,20E+08	1,97
146	Calmodulin-4 OS=Mus musculus GN=Calm4 PE=2 SV=2	Other proteins	CALM4_MOUSE	Calm4	4,57E+07	5,21E+07	3,73E+07	5,34E+07	3,77E+07	9,18E+07	1,35
147	Cofilin-1 OS=Mus musculus GN=Cfl1 PE=1 SV=3	Other proteins	COF1_MOUSE	Cfl1	1,18E+07	1,81E+07	1,69E+07	5,71E+07	5,31E+07	4,74E+07	3,36
148	Rab GDP dissociation inhibitor beta OS=Mus musculus GN=Gdi2 PE=1 SV=1	Other proteins	sp|Q61598|GDIB_MOUSE	Gdi2	9393100	2,54E+07	1,09E+07	2,76E+07	3,52E+07	3,74E+07	2,19
149	Destrin OS=Mus musculus GN=Dstn PE=1 SV=3	Other proteins	DEST_MOUSE	Dstn	1,88E+07	3,72E+07	1,77E+07	6,66E+07	7,22E+07	8,16E+07	2,99
150	Gasdermin-A OS=Mus musculus GN=Gsdma PE=2 SV=1	Other proteins	GSDMA_MOUSE	Gsdma	2,24E+07	3,01E+07	2,35E+07	2,92E+07	3,99E+07	4,27E+07	1,47
151	60S ribosomal protein L30 OS=Mus musculus GN=Rpl30 PE=1 SV=2	Ribosome	RL30_MOUSE	Rpl30	4,20E+07	7,90E+07	4,31E+07	8,41E+07	9,09E+07	1,11E+08	1,74
152	Ubiquitin-40S ribosomal protein S27a OS=Mus musculus GN=Rps27a PE=1 SV=2	Ribosome	RS27A_MOUSE	Rps27a	5,28E+07	8,29E+07	4,28E+07	9,10E+07	6,47E+07	8,92E+07	1,37
153	Isoform 3B of Desmocollin-3 OS=Mus musculus GN=Dsc3	Cell junctional proteins	sp|P55850-2|DSC3_MOUSE (+1)	Dsc3	6482700	7536700	1,61E+07	1,27E+07	1,24E+07	1,00E+07	1,17
154	Hornerin OS=Mus musculus GN=Hrnr PE=1 SV=1	Other proteins	E9QNP3_MOUSE (+1)	Hrnr	3950600	4845700	2924500	1,06E+07	2658100	8956000	1,89
155	Heat shock 70 kDa protein 1A OS=Mus musculus GN=Hspa1a PE=1 SV=2	Heat shock proteins	HS71A_MOUSE (+1)	Hspa1a	1,51E+07	1,79E+07	1,56E+07	3,34E+07	3,37E+07	3,47E+07	2,09
156	60S ribosomal protein L8 OS=Mus musculus GN=Rpl8 PE=1 SV=2	Ribosome	RL8_MOUSE	Rpl8	1,74E+07	1,68E+07	2,77E+07	4,15E+07	3,18E+07	4,93E+07	1,98
157	60S acidic ribosomal protein P1 OS=Mus musculus GN=Rplp1 PE=1 SV=1	Ribosome	RLA1_MOUSE	Rplp1	1,41E+08	3,38E+08	2,05E+08	3,12E+08	3,64E+08	4,02E+08	1,58
158	40S ribosomal protein S13 OS=Mus musculus GN=Rps13 PE=1 SV=1	Ribosome	Q921R2_MOUSE (+1)	Rps13	3,43E+07	5,14E+07	3,06E+07	9,42E+07	1,00E+08	1,07E+08	2,60
159	Predicted gene 10036 OS=Mus musculus GN=Gm10036 PE=3 SV=1	Ribosome	E9PYL9_MOUSE (+1)	Rpl11	2,93E+07	2,13E+07	3,13E+07	4,87E+07	5,25E+07	5,07E+07	1,85
160	40S ribosomal protein S20 OS=Mus musculus GN=Rps20 PE=1 SV=1	Ribosome	RS20_MOUSE	Rps20	8,44E+07	8,74E+07	7,15E+07	1,32E+08	1,72E+08	1,22E+08	1,75
161	40S ribosomal protein S12 OS=Mus musculus GN=Rps12 PE=1 SV=1	Ribosome	F7AEH4_MOUSE (+1)	Rps12	6,09E+07	1,27E+08	8,72E+07	1,90E+08	1,86E+08	1,79E+08	2,02
162	60S ribosomal protein L10 (Fragment) OS=Mus musculus GN=Rpl10 PE=1 SV=1	Ribosome	I7HLV2_MOUSE (+1)	Rpl10	4,11E+07	2,00E+07	3,59E+07	9,00E+07	5,81E+07	3,37E+07	1,87
163	Keratin-associated protein 8-1 OS=Mus musculus GN=Krtap8-1 PE=2 SV=2	KRTAP	KRA81_MOUSE	Krtap8-1	1,30E+09	2,58E+09	1,26E+09	1,70E+09	1,61E+09	1,83E+09	1,00
164	14-3-3 protein epsilon OS=Mus musculus GN=Ywhae PE=1 SV=1	Other proteins	1433E_MOUSE	Ywhae	7,15E+07	1,09E+08	8,63E+07	1,56E+08	1,58E+08	1,58E+08	1,76
165	Serine--tRNA ligase, cytoplasmic OS=Mus musculus GN=Sars PE=1 SV=1	Enzymes	Q8C483_MOUSE (+1)	Sars	2992000	1,31E+07	3658500	1,35E+07	1,81E+07	2,07E+07	2,65
166	40S ribosomal protein S26 OS=Mus musculus GN=Rps26 PE=1 SV=3	Ribosome	RS26_MOUSE	Rps26	5,26E+07	4,61E+07	5,12E+07	8,73E+07	8,14E+07	7,30E+07	1,61
167	60S ribosomal protein L18a (Fragment) OS=Mus musculus GN=Rpl18a PE=1 SV=1	Ribosome	A0A1D5RM85_MOUSE (+1)	Rpl18a	2,39E+07	1,40E+07	2,22E+07	4,54E+07	3,71E+07	3,11E+07	1,89
168	40S ribosomal protein S25 OS=Mus musculus GN=Rps25 PE=1 SV=1	Ribosome	A0A1L1SQA8_MOUSE (+1)	Rps25	4,01E+07	5,10E+07	4,85E+07	7,90E+07	9,04E+07	6,97E+07	1,71
169	Eukaryotic translation initiation factor 4 gamma 1 OS=Mus musculus GN=Eif4g1 PE=1 SV=1	Translation factors	A0A0J9YUS5_MOUSE (+5)	Eif4g1	2972500	6944700	1844600	8836000	1,14E+07	1,32E+07	2,84
170	Eukaryotic translation initiation factor 3 subunit C OS=Mus musculus GN=Eif3c PE=1 SV=1	Translation factors	EIF3C_MOUSE	Eif3c	3556400	7482600	5698500	1,10E+07	1,18E+07	1,14E+07	2,04
171	Eukaryotic translation initiation factor 3 subunit M OS=Mus musculus GN=Eif3m PE=1 SV=1	Translation factors	EIF3M_MOUSE	Eif3m	1,77E+07	2,83E+07	1,84E+07	4,35E+07	5,84E+07	3,91E+07	2,19
172	60S ribosomal protein L14 OS=Mus musculus GN=Rpl14 PE=1 SV=3	Ribosome	RL14_MOUSE	Rpl14	1,80E+07	4,62E+07	2,15E+07	4,00E+07	5,92E+07	6,51E+07	1,92
173	40S ribosomal protein S11 OS=Mus musculus GN=Rps11 PE=1 SV=3	Ribosome	RS11_MOUSE	Rps11	3,16E+07	2,47E+07	3,74E+07	6,12E+07	5,05E+07	3,23E+07	1,54
174	40S ribosomal protein S17 OS=Mus musculus GN=Rps17 PE=1 SV=2	Ribosome	RS17_MOUSE	Rps17	5,42E+07	6,55E+07	6,83E+07	1,11E+08	1,09E+08	1,17E+08	1,79
175	60S ribosomal protein L27 OS=Mus musculus GN=Rpl27 PE=1 SV=2	Ribosome	RL27_MOUSE	Rpl27	5,43E+07	8,49E+07	5,79E+07	1,07E+08	1,42E+08	1,01E+08	1,77
176	Protein disulfide-isomerase A3 OS=Mus musculus GN=Pdia3 PE=1 SV=2	Enzymes	PDIA3_MOUSE	Pdia3	960090	2002000	1312200	2,02E+07	2,41E+07	2,17E+07	15,43
177	40S ribosomal protein S15 OS=Mus musculus GN=Rps15 PE=1 SV=2	Ribosome	RS15_MOUSE	Rps15	3,55E+07	9,43E+07	4,90E+07	1,15E+08	9,96E+07	1,42E+08	1,99
178	Heterogeneous nuclear ribonucleoprotein A3 OS=Mus musculus GN=Hnrnpa3 PE=1 SV=1	Other proteins	A2AL12_MOUSE (+4)	Hnrnpa3	2,08E+07	4,46E+07	2,47E+07	1,82E+07	3,34E+07	1,68E+07	0,76
179	Eukaryotic translation initiation factor 3 subunit B OS=Mus musculus GN=Eif3b PE=1 SV=1	Translation factors	EIF3B_MOUSE	Eif3b	4419900	1,67E+07	6473800	1,92E+07	2,67E+07	3,13E+07	2,79
180	Eukaryotic translation initiation factor 3 subunit E OS=Mus musculus GN=Eif3e PE=1 SV=1	Translation factors	EIF3E_MOUSE	Eif3e	5338500	9955000	7581200	9648500	1,24E+07	2,63E+07	2,11
181	Isoform 2 of Prostatic acid phosphatase OS=Mus musculus GN=Acpp	Enzymes	sp|Q8CE08-2|PPAP_MOUSE (+1)	Acpp	1,57E+07	3,28E+07	1,56E+07	2,69E+07	2,88E+07	3,17E+07	1,36
182	Myosin-14 OS=Mus musculus GN=Myh14 PE=1 SV=1	Filaments	K3W4R2_MOUSE (+3)	Myh14	608760	849860	316210	5712900	6510200	7181400	10,93
183	Axin-2 OS=Mus musculus GN=Axin2 PE=1 SV=1	Other proteins	A2ABR0_MOUSE (+1)	Axin2	0	0	0	6109000	0	1,31E+07	>30,00
184	Ribosomal protein OS=Mus musculus GN=Rpl10a PE=1 SV=1	Ribosome	Q5XJF6_MOUSE	Rpl10a	3237000	4,80E+07	1,93E+07	6,90E+07	6,69E+07	6,23E+07	2,81
185	Predicted gene, 37797 OS=Mus musculus GN=Gm37797 PE=4 SV=1	Other proteins	A0A1D5RLC8_MOUSE	Gm37797	1,59E+07	8054100	2,73E+07	2,09E+07	1,79E+07	6466200	0,88
186	Carbonic anhydrase 2 OS=Mus musculus GN=Ca2 PE=1 SV=4	Enzymes	CAH2_MOUSE	Ca2	1,14E+07	1,05E+07	1,48E+07	2,57E+07	3,19E+07	3,03E+07	2,39
187	Eukaryotic translation initiation factor 3 subunit L OS=Mus musculus GN=Eif3l PE=1 SV=1	Translation factors	EIF3L_MOUSE	Eif3l	4112900	8596000	3776900	1,54E+07	1,26E+07	1,39E+07	2,55
188	Eukaryotic translation initiation factor 2 subunit 3, X-linked OS=Mus musculus GN=Eif2s3x PE=1 SV=2	Translation factors	IF2G_MOUSE	Eif2s3x	6515000	1,39E+07	1,87E+07	3,43E+07	4,17E+07	3,93E+07	2,95
189	60S ribosomal protein L31 OS=Mus musculus GN=Rpl31 PE=1 SV=1	Ribosome	A0A0A6YX26_MOUSE (+1)	Rpl31	3,79E+07	4,51E+07	4,41E+07	1,07E+08	1,20E+08	8,16E+07	2,42
190	Plastin-3 (Fragment) OS=Mus musculus GN=Pls3 PE=1 SV=1	Other proteins	A0A1C7CYV0_MOUSE (+2)	Pls3	1366700	3910500	5265700	1,72E+07	2,17E+07	2,65E+07	6,20
191	Ribosomal protein L19 OS=Mus musculus GN=Rpl19 PE=1 SV=1	Ribosome	A2A547_MOUSE (+1)	Rpl19	3,31E+07	7908500	3,63E+07	5,49E+07	5,88E+07	1,39E+07	1,65
192	Aldehyde dehydrogenase family 3 member B2 OS=Mus musculus GN=Aldh3b2 PE=1 SV=1	Enzymes	AL3B2_MOUSE	Aldh3b2	9609900	1,41E+07	8338100	2,74E+07	3,55E+07	3,19E+07	2,96
193	Acyl-CoA-binding protein OS=Mus musculus GN=Dbi PE=1 SV=2	Other proteins	ACBP_MOUSE (+1)	Dbi	2,71E+07	3,82E+07	2,83E+07	6,09E+07	3,75E+07	3,89E+07	1,47
194	Hypoxanthine-guanine phosphoribosyltransferase OS=Mus musculus GN=Hprt1 PE=1 SV=3	Enzymes	HPRT_MOUSE	Hprt1	8627000	2,94E+07	5288100	2,48E+07	2,94E+07	3,30E+07	2,01
195	Macrophage migration inhibitory factor OS=Mus musculus GN=Mif PE=1 SV=2	Other proteins	MIF_MOUSE	Mif	2,55E+07	7,00E+07	2,09E+07	5,75E+07	4,96E+07	8,15E+07	1,62
196	40S ribosomal protein S14 OS=Mus musculus GN=Rps14 PE=1 SV=3	Ribosome	RS14_MOUSE	Rps14	5,81E+07	1,24E+07	9,53E+07	1,09E+08	1,29E+08	6,60E+07	1,83
**197**	**Cluster of Keratin-associated protein 19-3 OS=Mus musculus GN=Krtap19-3 PE=2 SV=1 (KR193_MOUSE)**	KRTAP	**KR193_MOUSE [2]**	**Krtap19-3 Cluster**	2,91E+08	4,46E+08	2,61E+08	3,47E+08	3,37E+08	3,65E+08	1,05
197,1	Keratin-associated protein 19-3 OS=Mus musculus GN=Krtap19-3 PE=2 SV=1	KRTAP	KR193_MOUSE	Krtap19-3	2,26E+08	3,81E+08	2,30E+08	2,72E+08	2,67E+08	3,20E+08	1,03
197,2	Keratin-associated protein 16-3 OS=Mus musculus GN=Krtap16-3 PE=2 SV=1	KRTAP	KR163_MOUSE	Krtap16-3	3,57E+08	5,11E+08	2,91E+08	4,23E+08	4,07E+08	4,11E+08	1,07
198	Corneodesmosin OS=Mus musculus GN=Cdsn PE=2 SV=2	Cell junctional proteins	CDSN_MOUSE	Cdsn	6426100	1,39E+07	1,34E+07	1,80E+07	1,35E+07	1,34E+07	1,33
199	Ezrin OS=Mus musculus GN=Ezr PE=1 SV=3	Other proteins	EZRI_MOUSE	Ezr	6021700	9790400	6274600	7546300	1,15E+07	9580600	1,30
200	1-phosphatidylinositol 4,5-bisphosphate phosphodiesterase delta-1 OS=Mus musculus GN=Plcd1 PE=1 SV=2	Enzymes	PLCD1_MOUSE	Plcd1	2456300	4197700	3796800	4516400	5896600	8590700	1,82
201	Eukaryotic translation initiation factor 2 subunit 1 OS=Mus musculus GN=Eif2s1 PE=1 SV=3	Translation factors	IF2A_MOUSE	Eif2s1	8811800	6269100	8857400	1,92E+07	2,78E+07	2,05E+07	2,82
202	60S ribosomal protein L17 OS=Mus musculus GN=Rpl17 PE=1 SV=1	Ribosome	Q6ZWZ7_MOUSE (+1)	Rpl17	1,64E+07	2,91E+07	1,96E+07	5,76E+07	7,00E+07	9,27E+07	3,38
203	40S ribosomal protein S24 OS=Mus musculus GN=Rps24 PE=4 SV=1	Ribosome	A0A286YEB7_MOUSE (+3)	Rps24	5,72E+07	2,74E+07	4,82E+07	1,06E+08	1,23E+08	1,17E+08	2,60
204	60S ribosomal protein L26 (Fragment) OS=Mus musculus GN=Rpl26 PE=1 SV=1	Ribosome	B1ARA3_MOUSE (+1)	Rpl26	3,65E+07	1,41E+07	4,52E+07	6,79E+07	5,47E+07	2,81E+07	1,57
205	Unconventional myosin-VI OS=Mus musculus GN=Myo6 PE=1 SV=1	Filaments	E9PVU0_MOUSE (+5)	Myo6	544280	1652700	696290	4248900	5073700	7817100	5,92
206	Peroxiredoxin-6 OS=Mus musculus GN=Prdx6 PE=1 SV=3	Other proteins	PRDX6_MOUSE (+1)	Prdx6	1,24E+07	1,89E+07	1,03E+07	4,12E+07	3,76E+07	3,93E+07	2,83
207	V-set and immunoglobulin domain-containing protein 8 OS=Mus musculus GN=Vsig8 PE=2 SV=2	Other proteins	sp|Q6P3A4|VSIG8_MOUSE	Vsig8	1,73E+07	1,18E+07	2,30E+07	2,99E+07	3,98E+07	1,27E+07	1,58
**208**	**Cluster of Filaggrin OS=Mus musculus GN=Flg PE=1 SV=1 (A0A0A6YXH0_MOUSE)**	Other proteins	**A0A0A6YXH0_MOUSE [3]**	**Flg Cluster**	2,07E+07	5,02E+07	1,18E+07	4,32E+07	1,69E+07	3,12E+07	1,10
208,1	Filaggrin OS=Mus musculus GN=Flg PE=1 SV=1	Other proteins	A0A0A6YXH0_MOUSE (+1)	Flg	2,90E+07	7,16E+07	1,70E+07	5,84E+07	2,27E+07	4,74E+07	1,09
208,2	Filaggrin OS=Mus musculus GN=Flg PE=1 SV=1	Other proteins	A0A0A6YW46_MOUSE	Flg	1,25E+07	2,88E+07	6608300	2,81E+07	1,12E+07	1,49E+07	1,13
209	Eukaryotic translation initiation factor 3 subunit F OS=Mus musculus GN=Eif3f PE=1 SV=2	Translation factors	EIF3F_MOUSE	Eif3f	1,55E+07	3,22E+07	1,90E+07	4,04E+07	4,99E+07	4,94E+07	2,10
210	Myosin-9 OS=Mus musculus GN=Myh9 PE=1 SV=4	Filaments	MYH9_MOUSE	Myh9	69201	692090	0	4928800	6062000	5239500	21,32
211	Keratin associated protein 16-7 OS=Mus musculus GN=Krtap21-1 PE=2 SV=1	KRTAP	B9EK12_MOUSE (+1)	Krtap21-1	1,15E+09	7,92E+08	1,03E+09	1,02E+09	1,20E+09	8,28E+08	1,03
212	Ras GTPase-activating-like protein IQGAP1 OS=Mus musculus GN=Iqgap1 PE=1 SV=2	Other proteins	IQGA1_MOUSE	Iqgap1	1556700	2205900	1384800	4184800	7683000	6556900	3,58
213	Hephaestin-like protein 1 OS=Mus musculus GN=Hephl1 PE=2 SV=2	Other proteins	HPHL1_MOUSE	Hephl1	2668300	1,04E+07	6271200	6308000	8030200	7068100	1,11
214	Importin subunit beta-1 OS=Mus musculus GN=Kpnb1 PE=1 SV=2	Other proteins	IMB1_MOUSE	Kpnb1	2225700	5858500	1329200	7985800	1,34E+07	1,03E+07	3,36
215	Casein kinase I isoform alpha OS=Mus musculus GN=Csnk1a1 PE=1 SV=1	Enzymes	E9PWB2_MOUSE (+4)	Csnk1a1	1,12E+07	1,63E+07	8413700	1,16E+07	1,18E+07	1,41E+07	1,05
216	60 kDa heat shock protein, mitochondrial OS=Mus musculus GN=Hspd1 PE=1 SV=1	Heat shock proteins	sp|P63038|CH60_MOUSE	Hspd1	4553600	1,17E+07	1,07E+07	1,53E+07	1,28E+07	2,17E+07	1,85
217	RNA-binding protein 3 OS=Mus musculus GN=Rbm3 PE=1 SV=1	Other proteins	Q8BG13_MOUSE (+1)	Rbm3	3,45E+07	2,80E+07	3,80E+07	4,74E+07	6,17E+07	4,65E+07	1,55
218	Bifunctional glutamate/proline--tRNA ligase OS=Mus musculus GN=Eprs PE=1 SV=4	Enzymes	SYEP_MOUSE	Eprs	1594300	4665300	2115200	7243400	6228500	9512400	2,74
219	Ubiquitin-like modifier-activating enzyme 1 OS=Mus musculus GN=Uba1 PE=1 SV=1	Enzymes	UBA1_MOUSE	Uba1	533100	1507200	1361100	5206700	6833700	6344600	5,41
220	UPF0415 protein C7orf25 homolog OS=Mus musculus PE=2 SV=1	Other proteins	CG025_MOUSE	C7orf25	2,51E+07	1,63E+07	2,31E+07	8773300	1,92E+07	2,01E+07	0,74
221	Isoform Cytoplasmic+peroxisomal of Peroxiredoxin-5, mitochondrial OS=Mus musculus GN=Prdx5	Other proteins	sp|P99029-2|PRDX5_MOUSE (+1	Prdx5	7530200	3,06E+07	5303800	2,96E+07	3,99E+07	4,87E+07	2,73
222	40S ribosomal protein S23 OS=Mus musculus GN=Rps23 PE=1 SV=3	Ribosome	RS23_MOUSE	Rps23	1,75E+07	2,29E+07	2,61E+07	3,19E+07	4,59E+07	4,40E+07	1,83
223	Nascent polypeptide-associated complex subunit alpha, muscle-specific form OS=Mus musculus GN=Naca PE=1 SV=2	Other proteins	NACAM_MOUSE (+1)	Naca	1513000	3393700	1547700	3695000	3882900	5250100	1,99
224	60S ribosomal protein L24 OS=Mus musculus GN=Rpl24 PE=1 SV=2	Ribosome	RL24_MOUSE	Rpl24	1,91E+07	3,78E+07	2,35E+07	6,08E+07	7,19E+07	5,90E+07	2,38
225	Thioredoxin OS=Mus musculus GN=Txn PE=1 SV=3	Other proteins	THIO_MOUSE	Txn	4,42E+07	6,75E+07	3,35E+07	8,36E+07	1,05E+08	1,03E+08	2,01
226	Myosin light polypeptide 6 OS=Mus musculus GN=Myl6 PE=1 SV=1	Filaments	A0A1W2P6F6_MOUSE (+3)	Myl6	5252400	5173700	3634700	2,08E+07	2,00E+07	2,80E+07	4,89
227	Isoform 2 of Thioredoxin reductase 1, cytoplasmic OS=Mus musculus GN=Txnrd1	Other proteins	sp|Q9JMH6-2|TRXR1_MOUSE (+1	Txnrd1	4748400	1,50E+07	7512400	5345900	1,60E+07	1,24E+07	1,24
228	26S proteasome non-ATPase regulatory subunit 2 OS=Mus musculus GN=Psmd2 PE=1 SV=1	Proteasome	PSMD2_MOUSE	Psmd2	1996600	4805700	3213000	1,18E+07	1,47E+07	1,26E+07	3,90
229	Serine/threonine-protein phosphatase 2A 65 kDa regulatory subunit A beta isoform OS=Mus musculus GN=Ppp2r1b PE=	Enzymes	2AAB_MOUSE (+5)	Ppp2r1b	1203400	7725300	3750400	1,34E+07	1,86E+07	2,01E+07	4,10
230	Eukaryotic translation initiation factor 3 subunit I OS=Mus musculus GN=Eif3i PE=1 SV=1	Translation factors	EIF3I_MOUSE	Eif3i	2392400	4797000	3123600	9939700	1,11E+07	9109900	2,92
231	ATP-dependent RNA helicase DDX3X OS=Mus musculus GN=Ddx3x PE=1 SV=3	Enzymes	DDX3X_MOUSE	Ddx3x	4618000	5901800	7114400	5615500	8117500	6600300	1,15
232	Nucleolin OS=Mus musculus GN=Ncl PE=1 SV=2	Other proteins	NUCL_MOUSE	Ncl	2108100	2593400	5121100	3730300	4093800	2192800	1,02
233	60S ribosomal protein L5 OS=Mus musculus GN=Rpl5 PE=1 SV=3	Ribosome	RL5_MOUSE	Rpl5	9882000	1,26E+07	8107700	8771200	1,77E+07	1,65E+07	1,41
234	60S ribosomal protein L35a OS=Mus musculus GN=Rpl35a PE=1 SV=2	Ribosome	RL35A_MOUSE	Rpl35a	2,15E+07	2028000	2,39E+07	3,31E+07	2,04E+07	7584900	1,29
235	Cornifin-B OS=Mus musculus GN=Sprr1b PE=2 SV=1	Other proteins	SPR1B_MOUSE	Sprr1b	1,43E+07	855810	1,56E+07	1,46E+07	1,33E+07	1386100	0,95
236	Ribosomal protein L23A, pseudogene 3 OS=Mus musculus GN=Rpl23a-ps3 PE=3 SV=1	Ribosome	A0A140T8M7_MOUSE (+1)	Rpl23a-ps3	7100900	2,92E+07	0	6,49E+07	7,29E+07	4,81E+07	5,13
237	Poly(rC)-binding protein 1 OS=Mus musculus GN=Pcbp1 PE=1 SV=1	Other proteins	PCBP1_MOUSE	Pcbp1	8477100	1,52E+07	1,03E+07	1,59E+07	1,78E+07	2,29E+07	1,67
238	GTP-binding nuclear protein Ran OS=Mus musculus GN=Ran PE=1 SV=3	Other proteins	RAN_MOUSE	Ran	1,08E+07	1,33E+07	1,39E+07	1,42E+07	1,92E+07	1,72E+07	1,33
239	60S ribosomal protein L32 OS=Mus musculus GN=Rpl32 PE=1 SV=2	Ribosome	RL32_MOUSE	Rpl32	1,66E+07	1,86E+07	1,20E+07	4,76E+07	4,65E+07	4,16E+07	2,87
240	Myeloid leukemia factor 1 OS=Mus musculus GN=Mlf1 PE=1 SV=1	Other proteins	MLF1_MOUSE	Mlf1	1,75E+07	6547700	1,78E+07	1,44E+07	1,00E+07	1,30E+07	0,90
241	Heterogeneous nuclear ribonucleoprotein A1 OS=Mus musculus GN=Hnrnpa1 PE=1 SV=1	Other proteins	Q5EBP8_MOUSE (+2)	Hnrnpa1	1,06E+07	1,28E+07	7504700	1,02E+07	1,14E+07	1,34E+07	1,13
242	60S ribosomal protein L36 OS=Mus musculus GN=Rpl36 PE=1 SV=1	Ribosome	Q6ZWZ4_MOUSE	Rpl36	3,33E+07	5,24E+07	3,16E+07	7,39E+07	9,16E+07	9,30E+07	2,20
243	60S ribosomal protein L21 OS=Mus musculus GN=Rpl21 PE=1 SV=1	Ribosome	Q9CQM8_MOUSE (+1)	Rpl21	1,43E+07	3,84E+07	1,84E+07	6,46E+07	8,51E+07	6,03E+07	2,95
244	60S ribosomal protein L13a OS=Mus musculus GN=Rpl13a PE=1 SV=4	Ribosome	RL13A_MOUSE	Rpl13a	1,02E+07	1,17E+07	1,27E+07	1,65E+07	1,49E+07	1,49E+07	1,34
245	Protein S100-A16 OS=Mus musculus GN=S100a16 PE=1 SV=1	Other proteins	S10AG_MOUSE	S100a16	6,24E+07	3,44E+07	8,58E+07	9,19E+07	5,44E+07	1,01E+07	0,86
246	Isoform 2 of Heterogeneous nuclear ribonucleoproteins A2/B1 OS=Mus musculus GN=Hnrnpa2b1	Other proteins	sp|O88569-2|ROA2_MOUSE (+2)	Hnrnpa2b1	6665700	6064900	1,23E+07	1,17E+07	9119200	7738100	1,14
247	Dynein light chain 1, cytoplasmic OS=Mus musculus GN=Dynll1 PE=1 SV=1	Filaments	DYL1_MOUSE	Dynll1	4,86E+07	4,00E+07	5,14E+07	6,59E+07	7,02E+07	3,46E+07	1,22
248	Glycine--tRNA ligase OS=Mus musculus GN=Gars PE=1 SV=1	Enzymes	GARS_MOUSE	Gars	1688800	2407100	1368500	3271400	5820600	8450900	3,21
249	Elongation factor 1-beta OS=Mus musculus GN=Eef1b PE=1 SV=5	Translation factors	EF1B_MOUSE	Eef1b	7229200	7469100	1,62E+07	3,54E+07	3,24E+07	5,16E+07	3,87
250	Glucose-6-phosphate isomerase OS=Mus musculus GN=Gpi PE=1 SV=4	Enzymes	G6PI_MOUSE	Gpi	1988400	9056200	4166500	1,06E+07	1,01E+07	1,45E+07	2,31
251	Histone H1.5 OS=Mus musculus GN=Hist1h1b PE=1 SV=2	Histones	H15_MOUSE	Hist1h1b	1,98E+07	1,42E+07	2,23E+07	3,00E+07	2,56E+07	1,43E+07	1,24
252	Polypyrimidine tract-binding protein 1 OS=Mus musculus GN=Ptbp1 PE=1 SV=2	Other proteins	PTBP1_MOUSE (+2)	Ptbp1	8612300	2,14E+07	8254300	1,09E+07	1,94E+07	1,91E+07	1,29
253	T-complex protein 1 subunit zeta OS=Mus musculus GN=Cct6a PE=1 SV=3	Chaperonin	TCPZ_MOUSE	Cct6a	3286300	4561500	7081900	2,26E+07	2,91E+07	2,94E+07	5,43
254	60S ribosomal protein L27a OS=Mus musculus GN=Rpl27a PE=1 SV=5	Ribosome	RL27A_MOUSE	Rpl27a	2,03E+07	2,98E+07	2,59E+07	4,07E+07	4,83E+07	4,48E+07	1,76
255	Plasminogen activator inhibitor 2, macrophage OS=Mus musculus GN=Serpinb2 PE=1 SV=1	Other proteins	PAI2_MOUSE	Serpinb2	6252500	1,82E+07	6080900	2,12E+07	1,47E+07	2,67E+07	2,05
256	Elongation factor 1-delta OS=Mus musculus GN=Eef1d PE=1 SV=1	Translation factors	A0A0R4J1E2_MOUSE (+8)	Eef1d	2954000	4858200	3191300	5876300	7905000	7489600	1,93
257	Ribonuclease inhibitor OS=Mus musculus GN=Rnh1 PE=1 SV=1	Other proteins	A0A1B0GSG5_MOUSE (+1)	Rnh1	4289600	6176700	3021700	1,78E+07	1,62E+07	1,27E+07	3,46
258	Tyrosine--tRNA ligase OS=Mus musculus GN=Yars PE=1 SV=1	Enzymes	A2A7S7_MOUSE (+1)	Yars	2239900	4668400	2712100	6265600	9734500	1,10E+07	2,80
259	Citrate synthase, mitochondrial OS=Mus musculus GN=Cs PE=1 SV=1	Enzymes	CISY_MOUSE	Cs	2658500	6690200	1728300	2,09E+07	2,17E+07	2,04E+07	5,69
261	Spliceosome RNA helicase Ddx39b OS=Mus musculus GN=Ddx39b PE=1 SV=1	Enzymes	DX39B_MOUSE	Ddx39b	5763700	1,04E+07	4439300	4575700	4808600	7043400	0,80
262	Spermatogenesis-associated protein 13 OS=Mus musculus GN=Spata13 PE=1 SV=1	Other proteins	E9Q3I3_MOUSE	Spata13	1,05E+07	5373800	1,46E+07	1,16E+07	1,02E+07	5493800	0,90
263	Phospholipase A2 OS=Mus musculus GN=Pla2g4b PE=4 SV=1	Enzymes	B7ZCM8_MOUSE (+3)	Pla2g4b	3010300	6184800	2660200	1956000	4091200	4137100	0,86
264	Peptidyl-prolyl cis-trans isomerase B OS=Mus musculus GN=Ppib PE=1 SV=2	Enzymes	PPIB_MOUSE	Ppib	2064200	1344600	1656100	1,47E+07	1,51E+07	2,03E+07	9,88

#	Identified proteins	Protein group	Accession number	Symbol	WT1	WT2	WT3	KO1	KO2	KO3	Ratio KO/WT
265	T-complex protein 1 subunit epsilon OS=Mus musculus GN=Cct5 PE=1 SV=1	Chaperonin	TCPE_MOUSE	Cct5	801920	1854700	1141700	6429300	7484700	8088100	5,79
266	Protein S100-A14 OS=Mus musculus GN=S100a14 PE=1 SV=1	Other proteins	S10AE_MOUSE	S100a14	8,19E+07	1,02E+08	8,79E+07	5,24E+07	7,17E+07	8,44E+07	0,77
267	Isoform 3 of Probable hydrolase PNKD OS=Mus musculus GN=Pnkd	Enzymes	sp|Q69ZP3-3|PNKD_MOUSE (+2)	Pnkd	6,32E+07	2,98E+07	6,47E+07	5,59E+07	6,21E+07	2,73E+07	0,92
268	Dehydrogenase/reductase SDR family member 1 OS=Mus musculus GN=Dhrs1 PE=1 SV=1	Enzymes	DHRS1_MOUSE	Dhrs1	6605400	9938100	4620200	2,03E+07	1,89E+07	3,34E+07	3,43
269	T-complex protein 1 subunit gamma OS=Mus musculus GN=Cct3 PE=1 SV=1	Chaperonin	E9Q133_MOUSE (+1)	Cct3	443090	0	0	1,40E+07	2,13E+07	1,14E+07	105,34
270	Creatine kinase B-type OS=Mus musculus GN=Ckb PE=1 SV=1	Enzymes	KCRB_MOUSE	Ckb	4951100	7260800	5337300	1,45E+07	2,21E+07	2,34E+07	3,42
271	Zinc finger protein 651 (Fragment) OS=Mus musculus GN=Zfp651 PE=1 SV=1	Other proteins	A0A1L1STM6_MOUSE	Zfp651	1,14E+07	4434600	8835300	5156400	3802500	4730900	0,56
272	Rho GDP-dissociation inhibitor 1 OS=Mus musculus GN=Arhgdia PE=1 SV=3	Other proteins	GDIR1_MOUSE	Arhgdia	3882700	1,42E+07	8598100	1,18E+07	1,36E+07	1,98E+07	1,70
273	Isoform Mt-VDAC1 of Voltage-dependent anion-selective channel protein 1 OS=Mus musculus GN=Vdac1	Other proteins	sp|Q60932-2|VDAC1_MOUSE (+1	Vdac1	4655800	5484700	4807000	1,45E+07	1,23E+07	1,27E+07	2,64
274	Eukaryotic translation initiation factor 3 subunit H OS=Mus musculus GN=Eif3h PE=1 SV=1	Translation factors	EIF3H_MOUSE	Eif3h	3064200	3493400	3584400	1,08E+07	9139900	8489200	2,81
275	Protein FAM83H OS=Mus musculus GN=Fam83h PE=1 SV=1	Other proteins	FA83H_MOUSE	Fam83h	3174500	1,15E+07	4526500	4045300	6252900	7480200	0,92
276	Histone H1.0 OS=Mus musculus GN=H1f0 PE=2 SV=4	Histones	H10_MOUSE	H1f0	2,01E+07	2,41E+07	2,88E+07	3,03E+07	2,64E+07	2,94E+07	1,18
277	Protein S100-A11 OS=Mus musculus GN=S100a11 PE=1 SV=1	Other proteins	S10AB_MOUSE	S100a11	2,68E+07	8,93E+07	5,85E+07	4,58E+07	4,47E+07	1,08E+08	1,14
278	Keratin-associated protein 22-2 OS=Mus musculus GN=Krtap22-2 PE=4 SV=1	KRTAP	J3QNX6_MOUSE	Krtap22-2	1,67E+08	1,46E+08	1,28E+08	1,22E+08	3,36E+07	1,23E+08	0,63
279	14-3-3 protein gamma OS=Mus musculus GN=Ywhag PE=1 SV=2	Other proteins	1433G_MOUSE	Ywhag	6,89E+07	1,08E+08	8,32E+07	1,44E+08	1,47E+08	1,64E+08	1,75
280	Thioredoxin domain-containing protein 17 OS=Mus musculus GN=Txndc17 PE=1 SV=1	Other proteins	TXD17_MOUSE	Txndc17	1,39E+07	1,89E+07	1,06E+07	3,75E+07	4,84E+07	3,80E+07	2,85
281	60S ribosomal protein L22 OS=Mus musculus GN=Rpl22 PE=1 SV=2	Ribosome	RL22_MOUSE	Rpl22	1,33E+07	7,80E+07	3,63E+07	9,83E+07	4,18E+07	1,32E+08	2,13
282	Plasminogen activator inhibitor 1 RNA-binding protein OS=Mus musculus GN=Serbp1 PE=1 SV=1	Other proteins	A0A0N4SV32_MOUSE (+4)	Serbp1	5887200	8310800	8694500	1,12E+07	1,11E+07	1,19E+07	1,49
283	Tropomyosin alpha-3 chain OS=Mus musculus GN=Tpm3 PE=1 SV=1	Filaments	D3Z6I8_MOUSE (+2)	Tpm3	2898900	0	3175900	7420200	1,10E+07	1,06E+07	4,79
284	Eukaryotic translation initiation factor 3 subunit D OS=Mus musculus GN=Eif3d PE=1 SV=2	Translation factors	EIF3D_MOUSE	Eif3d	2267500	6755500	4268600	6942900	1,27E+07	8707200	2,14
285	Isoform 1 of Core histone macro-H2A.1 OS=Mus musculus GN=H2afy	Histones	sp|Q9QZQ8-2|H2AY_MOUSE (+1	H2afy	9790100	1,89E+07	1,03E+07	1,32E+07	1,68E+07	1,70E+07	1,20
286	Isoform 3 of Programmed cell death 6-interacting protein OS=Mus musculus GN=Pdcd6ip	Other proteins	sp|Q9WU78-3|PDC6I_MOUSE (+	Pdcd6ip	2605300	3677700	3165000	2575300	5711800	5729700	1,48
287	Actin-related protein 3 OS=Mus musculus GN=Actr3 PE=1 SV=3	Filaments	ARP3_MOUSE	Actr3	3103200	4318800	6197800	2,07E+07	2,19E+07	2,55E+07	5,00
288	Protein S100-A4 (Fragment) OS=Mus musculus GN=S100a4 PE=1 SV=1	Other proteins	A0A0G2JGD2_MOUSE (+1)	S100a4	3,11E+07	6,64E+07	2,42E+07	4,88E+07	3,75E+07	6,38E+07	1,23
289	T-complex protein 1 subunit delta OS=Mus musculus GN=Cct4 PE=1 SV=1	Chaperonin	G5E839_MOUSE (+1)	Cct4	0	946210	745280	8778600	6203100	9747000	14,62
290	60S ribosomal protein L38 OS=Mus musculus GN=Rpl38 PE=1 SV=3	Ribosome	RL38_MOUSE	Rpl38	1,62E+07	4,70E+07	1,11E+07	9,30E+07	6,21E+07	5,43E+07	2,82
291	Serpin B11 OS=Mus musculus GN=Serpinb11 PE=2 SV=1	Other proteins	SPB11_MOUSE	Serpinb11	3309500	9561800	3942700	3541800	7213400	1,25E+07	1,39
292	Protein Niban OS=Mus musculus GN=Fam129a PE=1 SV=1	Other proteins	E9PYV4_MOUSE (+1)	Fam129a	0	496630	385870	4471500	8134500	1,15E+07	27,37
293	Endoplasmin OS=Mus musculus GN=Hsp90b1 PE=1 SV=2	Other proteins	ENPL_MOUSE	Hsp90b1	2176200	1565900	2904500	1,27E+07	9645800	5808100	4,24
294	Translationally-controlled tumor protein OS=Mus musculus GN=Tpt1 PE=1 SV=1	Other proteins	TCTP_MOUSE	Tpt1	2,22E+07	3,35E+07	3,08E+07	3,49E+07	2,44E+07	6,70E+07	1,46
295	60S ribosomal protein L35 OS=Mus musculus GN=Rpl35 PE=1 SV=1	Ribosome	RL35_MOUSE	Rpl35	4,82E+07	1,07E+07	4,75E+07	1,03E+08	9,87E+07	2,71E+07	2,15
296	Keratin-associated protein 19-5 OS=Mus musculus GN=Krtap19-5 PE=1 SV=1	KRTAP	F8VQ65_MOUSE	Krtap19-5	3,22E+07	6,73E+07	3,15E+07	5,69E+07	3,64E+07	6,66E+07	1,22
297	Eukaryotic translation initiation factor 5 OS=Mus musculus GN=Eif5 PE=1 SV=1	Translation factors	IF5_MOUSE	Eif5	494000	2549600	605090	2406100	2825500	5044900	2,82
298	Non-canonical poly(A) RNA polymerase PAPD5 OS=Mus musculus GN=Papd5 PE=1 SV=1	Enzymes	E9QJT6_MOUSE (+3)	Papd5	9352200	1,59E+07	2909200	7062400	3682900	4545800	0,54
299	Fatty acid synthase OS=Mus musculus GN=Fasn PE=1 SV=1	Enzymes	A0A0U1RNJ1_MOUSE (+1)	Fasn	662220	625960	550300	1712800	1602200	4992600	4,52
300	Annexin A7 OS=Mus musculus GN=Anxa7 PE=1 SV=2	Other proteins	ANXA7_MOUSE	Anxa7	4819000	1,23E+07	4628400	7490400	4535200	6806100	0,87
301	Beta-hexosaminidase subunit beta OS=Mus musculus GN=Hexb PE=1 SV=2	Enzymes	HEXB_MOUSE	Hexb	3636800	4974000	4046100	5077600	6275700	6619100	1,42
302	Lamin-B1 OS=Mus musculus GN=Lmnb1 PE=1 SV=3	Other proteins	LMNB1_MOUSE	Lmnb1	4853800	1,21E+07	4644800	8201600	1,22E+07	1,21E+07	1,51
303	40S ribosomal protein S21 OS=Mus musculus GN=Rps21 PE=1 SV=1	Ribosome	RS21_MOUSE	Rps21	2,37E+07	9593400	1,40E+07	1,50E+07	3,02E+07	6608800	1,10
304	T-complex protein 1 subunit alpha OS=Mus musculus GN=Tcp1 PE=1 SV=3	Chaperonin	sp|P11983|TCPA_MOUSE	Tcp1	0	0	773590	6425500	9471800	4228200	26,02
305	T-complex protein 1 subunit theta OS=Mus musculus GN=Cct8 PE=1 SV=3	Chaperonin	TCPQ_MOUSE	Cct8	0	0	0	5943700	6173800	7220900	>30,00
306	Serine protease inhibitor Kazal-type 6 OS=Mus musculus GN=Spink6 PE=2 SV=1	Other proteins	ISK6_MOUSE	Spink6	4,43E+07	2,98E+07	4,45E+07	3,82E+07	4,21E+07	1,96E+07	0,84
307	Heterogeneous nuclear ribonucleoprotein K OS=Mus musculus GN=Hnrnpk PE=1 SV=1	Other proteins	B2M1R6_MOUSE (+4)	Hnrnpk	1990600	6892000	5165100	5277200	8897100	3731400	1,27
308	Deoxyribonuclease-1-like 2 OS=Mus musculus GN=Dnase1l2 PE=2 SV=1	Enzymes	DNSL2_MOUSE	Dnase1l2	0	0	0	1,62E+07	1,45E+07	1,61E+07	>30,00
309	Methionine aminopeptidase 2 OS=Mus musculus GN=Metap2 PE=1 SV=1	Enzymes	MAP2_MOUSE (+2)	Metap2	1765300	1197500	2847000	7573900	3766100	5578200	2,91
310	Serine/threonine-protein phosphatase PP1-alpha catalytic subunit OS=Mus musculus GN=Ppp1ca PE=1 SV=1	Enzymes	PP1A_MOUSE (+3)	Ppp1ca	8625400	1,04E+07	7986300	1,13E+07	1,14E+07	1,20E+07	1,29
311	Adenylyl cyclase-associated protein 1 OS=Mus musculus GN=Cap1 PE=1 SV=4	Other proteins	CAP1_MOUSE	Cap1	2211600	3126600	4155600	1,12E+07	1,00E+07	1,11E+07	3,40
312	Ras-related protein Rab-18 OS=Mus musculus GN=Rab18 PE=1 SV=2	Other proteins	RAB18_MOUSE	Rab18	0	1706400	0	1,30E+07	1,36E+07	1,11E+07	22,07
313	Filamin-B OS=Mus musculus GN=Flnb PE=1 SV=3	Filaments	FLNB_MOUSE	Flnb	267320	0	560900	498860	337540	848710	2,03
314	Cytochrome c oxidase subunit 2 OS=Mus musculus GN=Mtco2 PE=1 SV=1	Enzymes	COX2_MOUSE	Mt-co2	1,11E+07	1,69E+07	5030800	2,15E+07	1,71E+07	2,99E+07	2,08
315	Creatine kinase U-type, mitochondrial OS=Mus musculus GN=Ckmt1 PE=1 SV=1	Enzymes	KCRU_MOUSE	Ckmt1	2335700	1,28E+07	3281100	1,28E+07	1,30E+07	1,85E+07	2,41
316	T-complex protein 1 subunit eta OS=Mus musculus GN=Cct7 PE=1 SV=1	Chaperonin	TCPH_MOUSE	Cct7	732390	1966200	2518200	6675400	1,13E+07	8202100	5,01
317	Isoform 2 of Sarcoplasmic/endoplasmic reticulum calcium ATPase 2 OS=Mus musculus GN=Atp2a2	Enzymes	sp|O55143-2|AT2A2_MOUSE (+1	Atp2a2	0	0	0	4264900	4862800	5241100	>30,00
318	Histone H2A.V OS=Mus musculus GN=H2afv PE=1 SV=3	Histones	H2AV_MOUSE (+1)	H2afv	4,30E+08	4,94E+08	4,55E+08	6,65E+08	8,44E+08	3,57E+08	1,35
319	Predicted gene 6358 OS=Mus musculus GN=Gm6358 PE=4 SV=1	KRTAP	A0A087WPH4_MOUSE (+4)	Krtap Gm6358	9,27E+08	2,11E+08	2,11E+08	1,34E+08	1,71E+08	1,46E+08	0,33
320	Keratin, type I cytoskeletal 28 OS=Mus musculus GN=Krt28 PE=1 SV=1	Keratins	K1C28_MOUSE	Krt28	4,86E+08	1,51E+08	4,99E+08	3,52E+08	1,60E+08	1,29E+08	0,56
321	6-phosphogluconate dehydrogenase, decarboxylating OS=Mus musculus GN=Pgd PE=1 SV=3	Enzymes	6PGD_MOUSE	Pgd	1342800	2103500	2342300	3001700	3547500	5051200	2,00
322	Trifunctional enzyme subunit alpha, mitochondrial OS=Mus musculus GN=Hadha PE=1 SV=1	Enzymes	ECHA_MOUSE	Hadha	961330	4513200	3887800	7792200	1,09E+07	4593500	2,49
323	Threonine--tRNA ligase, cytoplasmic OS=Mus musculus GN=Tars PE=1 SV=2	Enzymes	SYTC_MOUSE	Tars	276790	1772200	0	1896100	2407300	1797500	2,98
324	Vacuolar protein sorting-associated protein 35 OS=Mus musculus GN=Vps35 PE=1 SV=1	Other proteins	VPS35_MOUSE	Vps35	1075300	2592600	0	1646500	1885000	1207000	1,29
325	Calmodulin-like protein 3 OS=Mus musculus GN=Calml3 PE=2 SV=1	Other proteins	CALL3_MOUSE	Calml3	1,54E+07	6,95E+07	1,36E+07	1,00E+08	7,57E+07	1,27E+08	3,08
326	Aspartate aminotransferase, mitochondrial OS=Mus musculus GN=Got2 PE=1 SV=1	Enzymes	AATM_MOUSE	Got2	622180	2151200	1380400	4257000	3587800	5767200	3,28
327	Alpha-centractin OS=Mus musculus GN=Actr1a PE=1 SV=1	Other proteins	ACTZ_MOUSE	Actr1a	5022200	2115700	4569400	1,03E+07	2708600	4270100	1,48
328	Actin-related protein 2/3 complex subunit 4 OS=Mus musculus GN=Arpc4 PE=1 SV=3	Other proteins	ARPC4_MOUSE	Arpc4	7752900	7864000	4589400	2,58E+07	2,39E+07	1,51E+07	3,21
329	Plectin OS=Mus musculus GN=Plec PE=1 SV=1	Cell junctional proteins	E9Q3W4_MOUSE (+16)	Plec	299870	159450	460140	347560	202930	174530	0,79
330	Transgelin-2 OS=Mus musculus GN=Tagln2 PE=1 SV=4	Other proteins	TAGL2_MOUSE	Tagln2	2352300	0	2950500	1,08E+07	1,22E+07	1,27E+07	6,73
331	T-complex protein 1 subunit beta OS=Mus musculus GN=Cct2 PE=1 SV=4	Chaperonin	TCPB_MOUSE	Cct2	0	0	0	5819400	6566500	9460300	>30,00
332	Ubiquitin carboxyl-terminal hydrolase isozyme L3 OS=Mus musculus GN=Uchl3 PE=1 SV=2	Enzymes	UCHL3_MOUSE	Uchl3	2273700	5000300	1900000	8609300	1,23E+07	6923300	3,03
334	Glycolipid transfer protein OS=Mus musculus GN=Gltp PE=1 SV=3	Other proteins	GLTP_MOUSE	Gltp	1857700	3265800	1587300	3265300	5405800	1,01E+07	2,79
335	Cell division control protein 42 homolog OS=Mus musculus GN=Cdc42 PE=1 SV=2	Other proteins	sp|P60766|CDC42_MOUSE	Cdc42	0	2100400	0	9254800	1,08E+07	6510200	12,67
336	MCG119409 OS=Mus musculus GN=Sprr2b PE=2 SV=1	Other proteins	A9JTY7_MOUSE (+1)	Sprr2b	7811900	0	1,09E+07	6221400	1,99E+07	2909000	1,55
337	Eukaryotic translation initiation factor 5B OS=Mus musculus GN=Eif5b PE=1 SV=2	Translation factors	IF2P_MOUSE	Eif5b	1500800	2475400	505650	3475400	3696300	3722000	2,43
338	Phosphate carrier protein, mitochondrial OS=Mus musculus GN=Slc25a3 PE=1 SV=1	Other proteins	MPCP_MOUSE	Slc25a3	2547900	3403800	2167600	7712900	1,05E+07	7080900	3,11
339	V-type proton ATPase subunit B, brain isoform OS=Mus musculus GN=Atp6v1b2 PE=1 SV=1	Enzymes	VATB2_MOUSE	Atp6v1b2	3289700	5794100	1965400	8194900	8976100	1,01E+07	2,47
340	Suprabasin OS=Mus musculus GN=Sbsn PE=1 SV=1	Other proteins	E9QPB2_MOUSE (+1)	Sbsn	2267700	1624700	1469800	3097300	9564200	170330	2,39
341	Calpain-12 OS=Mus musculus GN=Capn12 PE=3 SV=1	Enzymes	E9QL26_MOUSE (+1)	Capn12	1090400	3523800	2136000	433140	1051300	0	0,22
342	Casein kinase I isoform epsilon OS=Mus musculus GN=Csnk1e PE=1 SV=2	Enzymes	KC1E_MOUSE (+3)	Csnk1e	4166200	5622000	3991600	2994100	2857200	3949600	0,71
343	26S proteasome non-ATPase regulatory subunit 1 OS=Mus musculus GN=Psmd1 PE=1 SV=1	Proteasome	PSMD1_MOUSE	Psmd1	186540	300580	0	2411800	2801900	741620	12,23
344	Isoform 2 of Ras-related protein Rab-6A OS=Mus musculus GN=Rab6a	Other proteins	sp|P35279-2|RAB6A_MOUSE (+1	Rab6a	2804000	3238000	2217900	1,27E+07	9788300	1,08E+07	4,02
345	V-type proton ATPase catalytic subunit A OS=Mus musculus GN=Atp6v1a PE=1 SV=2	Enzymes	sp|P50516|VATA_MOUSE	Atp6v1a	418870	484880	0	1583600	4294400	3096000	9,93
346	Acyl-coenzyme A thioesterase THEM5 OS=Mus musculus GN=Them5 PE=1 SV=1	Enzymes	A0A0G2JEK7_MOUSE (+2)	Them5	3818600	4266200	1686100	9914100	1,28E+07	6021300	2,94
347	Isoform 2 of Transcription factor BTF3 OS=Mus musculus GN=Btf3	Other proteins	sp|Q64152-2|BTF3_MOUSE (+1)	Btf3	1,61E+07	3,66E+07	1,31E+07	4,11E+07	3,82E+07	5,34E+07	2,02
349	40S ribosomal protein S28 (Fragment) OS=Mus musculus GN=Rps28 PE=1 SV=1	Ribosome	G3UYV7_MOUSE (+1)	Rps28	2,23E+07	9503400	2,57E+07	2,09E+07	2,75E+07	1,32E+07	1,07
351	Spectrin alpha chain, non-erythrocytic 1 OS=Mus musculus GN=Sptan1 PE=1 SV=1	Other proteins	A3KGU5_MOUSE (+5)	Sptan1	204540	897910	451240	229150	332550	372310	0,60
352	26S proteasome non-ATPase regulatory subunit 6 OS=Mus musculus GN=Psmd6 PE=1 SV=1	Proteasome	PSMD6_MOUSE	Psmd6	1575100	791580	1468300	2849500	4883800	5973000	3,57
353	Desmocollin 1, isoform CRA_a OS=Mus musculus GN=Dsc1 PE=1 SV=1	Cell junctional proteins	Q32ME9_MOUSE (+2)	Dsc1	1092000	2329900	4531700	2606600	2812900	2527300	1,00
354	G-protein coupled receptor family C group 5 member D OS=Mus musculus GN=Gprc5d PE=2 SV=2	Other proteins	sp|Q9JIL6|GPC5D_MOUSE	Gprc5d	5719700	3138500	8969200	8585500	6702400	2594000	1,00
355	Small nuclear ribonucleoprotein Sm D3 OS=Mus musculus GN=Snrpd3 PE=1 SV=1	Other proteins	SMD3_MOUSE	Snrpd3	1854000	3087100	1778300	5628400	5206300	3179600	2,09
356	Cullin-associated NEDD8-dissociated protein 1 OS=Mus musculus GN=Cand1 PE=1 SV=2	Other proteins	CAND1_MOUSE	Cand1	232980	1084300	0	2237300	1102900	4472600	5,93
357	Prohibitin-2 OS=Mus musculus GN=Phb2 PE=1 SV=1	Other proteins	PHB2_MOUSE	Phb2	486410	1157300	0	2603600	4275700	5685500	7,64
358	Keratin-associated protein 27-1 OS=Mus musculus GN=Krtap27-1 PE=4 SV=1	KRTAP	E9PX37_MOUSE	Krtap27-1	3,69E+07	0	3,52E+07	1,08E+07	2,73E+07	4980700	0,60
359	Ras-related protein Rab-10 OS=Mus musculus GN=Rab10 PE=1 SV=1	Other proteins	RAB10_MOUSE	Rab10	3080700	5078600	3851800	1,04E+07	1,08E+07	1,20E+07	2,76
360	60S ribosomal protein L34 OS=Mus musculus GN=Rpl34 PE=1 SV=2	Ribosome	RL34_MOUSE	Rpl34	2,53E+07	0	2,47E+07	4,01E+07	3,65E+07	5566600	1,65
362	Carboxylic ester hydrolase OS=Mus musculus GN=Ces2g PE=1 SV=1	Enzymes	E9PV38_MOUSE	Ces2g	1685800	1543500	2857100	7651900	1,05E+07	1,50E+07	5,46
363	Glucose-6-phosphate 1-dehydrogenase X OS=Mus musculus GN=G6pdx PE=1 SV=3	Enzymes	G6PD1_MOUSE	G6pdx	0	0	0	1990300	4138800	4331300	>30,00
364	26S proteasome regulatory subunit 10B OS=Mus musculus GN=Psmc6 PE=1 SV=1	Proteasome	PRS10_MOUSE	Psmc6	0	661480	0	5149000	3069000	4059000	18,56
365	Isoform 1 of Cytosolic 5'-nucleotidase 3A OS=Mus musculus GN=Nt5c3a	Enzymes	sp|Q9D020-1|5NT3A_MOUSE (+1	Nt5c3a	9817700	9385300	8808300	8627600	9088300	1,40E+07	1,13
366	Isoform 2 of Cysteine--tRNA ligase, cytoplasmic OS=Mus musculus GN=Cars	Enzymes	sp|Q9ER72-2|SYCC_MOUSE (+1)	Cars	521740	0	0	5318500	2988900	2885300	21,45
367	ATP-binding cassette sub-family D member 3 OS=Mus musculus GN=Abcd3 PE=1 SV=1	Other proteins	A0A0G2JDI9_MOUSE (+1)	Abcd3	485560	1060100	918680	2115300	3167000	1266800	2,66
368	Protein S100-A6 OS=Mus musculus GN=S100a6 PE=1 SV=3	Other proteins	S10A6_MOUSE	S100a6	2,83E+07	7,22E+07	4,57E+07	6,18E+07	5,98E+07	8,89E+07	1,44
369	Cornifelin OS=Mus musculus GN=Cnfn PE=1 SV=1	Other proteins	CNFN_MOUSE (+1)	Cnfn	8,18E+07	1,94E+08	8,58E+07	1,22E+08	1,48E+08	2,26E+08	1,37
370	Ubiquitin-conjugating enzyme E2 N OS=Mus musculus GN=Ube2n PE=1 SV=1	Enzymes	UBE2N_MOUSE	Ube2n	0	2862900	0	1,60E+07	1,37E+07	1,19E+07	14,51
371	Vinculin OS=Mus musculus GN=Vcl PE=1 SV=4	Other proteins	VINC_MOUSE	Vcl	204160	1461100	1598900	1259200	996290	887370	0,96
372	Importin-5 OS=Mus musculus GN=Ipo5 PE=1 SV=3	Other proteins	sp|Q8BKC5|IPO5_MOUSE	Ipo5	323230	1711400	509780	1504700	1453500	2891500	2,30
373	60S ribosomal protein L28 OS=Mus musculus GN=Rpl28 PE=1 SV=2	Ribosome	RL28_MOUSE	Rpl28	1,20E+07	0	1,51E+07	6655900	4993300	8724000	0,75
374	Proteolipid protein 2 OS=Mus musculus GN=Plp2 PE=1 SV=1	Other proteins	PLP2_MOUSE	Plp2	5105600	1689000	5100200	7122900	3324700	1995500	1,05
375	Neurofilament light polypeptide OS=Mus musculus GN=Nefl PE=1 SV=5	Other proteins	NFL_MOUSE	Nefl	1,67E+08	9,84E+07	1,43E+08	1,72E+08	1,58E+08	9,72E+07	1,05
376	Annexin A8 OS=Mus musculus GN=Anxa8 PE=1 SV=2	Other proteins	ANXA8_MOUSE	Anxa8	1246500	4596900	786860	6799100	4013100	2628300	2,03
377	Proteasome subunit alpha type-2 OS=Mus musculus GN=Psma2 PE=1 SV=3	Proteasome	PSA2_MOUSE	Psma2	0	4751600	2460900	1,32E+07	1,32E+07	1,92E+07	6,32
378	Eukaryotic translation initiation factor 3 subunit G OS=Mus musculus GN=Eif3g PE=1 SV=2	Translation factors	EIF3G_MOUSE	Eif3g	1979700	3067100	0	3443600	3540900	3515400	2,08
379	Nck-associated protein 1 OS=Mus musculus GN=Nckap1 PE=1 SV=1	Other proteins	A2AS98_MOUSE (+2)	Nckap1	1622600	648500	1849700	1310100	698870	718390	0,66
380	Prosaposin OS=Mus musculus GN=Psap PE=1 SV=1	Other proteins	E9PZ00_MOUSE (+4)	Psap	2603900	9317500	3415100	0	2808300	4251700	0,46
381	Eukaryotic peptide chain release factor subunit 1 OS=Mus musculus GN=Etf1 PE=1 SV=4	Translation factors	ERF1_MOUSE	Etf1	0	960840	2063100	3938600	4111800	1678100	3,22
382	MCG11048, isoform CRA_c OS=Mus musculus GN=Tecr PE=1 SV=1	Other proteins	G3UWE1_MOUSE (+2)	Tecr	667460	1374400	0	6570700	4699000	3881800	7,42
383	Malate dehydrogenase, cytoplasmic OS=Mus musculus GN=Mdh1 PE=1 SV=3	Enzymes	MDHC_MOUSE	Mdh1	0	1699600	0	4863700	2305700	2203100	5,51
384	Proteasome subunit alpha type-6 OS=Mus musculus GN=Psma6 PE=1 SV=1	Proteasome	PSA6_MOUSE	Psma6	0	2586800	0	1,09E+07	1,20E+07	6771400	11,49
385	ATP-dependent RNA helicase A OS=Mus musculus GN=Dhx9 PE=1 SV=1	Enzymes	A0A087WPL5_MOUSE (+1)	Dhx9	587710	1034300	788230	214110	818770	575640	0,67
386	Eukaryotic translation initiation factor 2B, subunit 3 OS=Mus musculus GN=Eif2b3 PE=1 SV=1	Translation factors	B1AUN2_MOUSE (+2)	Eif2b3	2733200	5160400	3581800	5856400	1,03E+07	9953100	2,28
387	Peroxiredoxin-4 (Fragment) OS=Mus musculus GN=Prdx4 PE=1 SV=1	Other proteins	B1AZS9_MOUSE (+1)	Prdx4	0	1,28E+07	4073800	2,16E+07	2,26E+07	2,12E+07	3,88
388	Isoform 2 of Rotatin OS=Mus musculus GN=Rttn	Other proteins	sp|Q8R4Y8-2|RTTN_MOUSE (+1)	Rttn	1592200	0	1456100	2977900	2902400	762700	2,18
389	Calreticulin OS=Mus musculus GN=Calr PE=1 SV=1	Other proteins	CALR_MOUSE	Calr	726170	0	765740	5509900	3304700	4840900	9,15
390	Isoform 2 of Poly(U)-specific endoribonuclease OS=Mus musculus GN=Endou	Enzymes	sp|Q3V188-2|ENDOU_MOUSE (+	Endou	0	0	0	8103300	1,26E+07	5596400	>30,00
391	Isoform 2 of Proteasome-associated protein ECM29 homolog OS=Mus musculus GN=Ecm29	Proteasome	sp|Q6PDI5-2|ECM29_MOUSE (+1	Ecm29	68058	0	136450	188840	1676000	598410	12,04
392	26S proteasome regulatory subunit 6A OS=Mus musculus GN=Psmc3 PE=1 SV=1	Proteasome	A2AGN7_MOUSE (+2)	Psmc3	0	0	0	2299200	1096700	3315600	>30,00
393	Heterogeneous nuclear ribonucleoprotein A/B OS=Mus musculus GN=Hnrnpab PE=1 SV=1	Other proteins	Q20BD0_MOUSE (+2)	Hnrnpab	874830	5785300	2341200	4589200	6665800	1,23E+07	2,62
394	Heterogeneous nuclear ribonucleoprotein Q OS=Mus musculus GN=Syncrip PE=1 SV=1	Other proteins	A0A0R4J259_MOUSE (+5)	Syncrip	807720	1256700	717350	2221000	2206800	1588300	2,16
395	Calmodulin-1 OS=Mus musculus GN=Calm1 PE=1 SV=1	Other proteins	CALM1_MOUSE (+2)	Calm1	7859100	6185600	7822500	2,43E+07	2,60E+07	3,37E+07	3,84
396	40S ribosomal protein S27 (Fragment) OS=Mus musculus GN=Rps27 PE=1 SV=1	Ribosome	A0A0G2JDW7_MOUSE (+2)	Rps27	1,07E+07	8542200	1,19E+07	2,64E+07	7666100	6838100	1,32
397	60S ribosomal protein L39 OS=Mus musculus GN=Rpl39 PE=1 SV=2	Ribosome	RL39_MOUSE	Rpl39	3,00E+07	3,41E+07	2,39E+07	8,59E+07	4,56E+07	5,91E+07	2,17
399	26S proteasome non-ATPase regulatory subunit 3 OS=Mus musculus GN=Psmd3 PE=1 SV=3	Proteasome	PSMD3_MOUSE	Psmd3	862980	410620	0	520580	3381200	930990	3,79
400	Alcohol dehydrogenase [NADP(+)] OS=Mus musculus GN=Akr1a1 PE=1 SV=3	Enzymes	AK1A1_MOUSE	Akr1a1	332370	4489100	0	179040	5099400	6570600	2,46
401	Isoform 2 of Protein transport protein Sec31A OS=Mus musculus GN=Sec31a	Other proteins	sp|Q3UPL0-2|SC31A_MOUSE (+1	Sec31a	1388900	5858400	1225400	2424300	2230300	0	0,55
402	ATP synthase subunit O, mitochondrial OS=Mus musculus GN=Atp5o PE=1 SV=1	Enzymes	ATPO_MOUSE	Atp5o	1792300	8280400	0	1,17E+07	1,41E+07	1,37E+07	3,93
403	Eukaryotic translation initiation factor 3 subunit J-A OS=Mus musculus GN=Eif3j1 PE=2 SV=1	Translation factors	EI3JA_MOUSE (+1)	Eif3j1	1998600	3059500	0	6558700	5958400	8191000	4,09
404	Superoxide dismutase [Cu-Zn] OS=Mus musculus GN=Sod1 PE=1 SV=2	Enzymes	SODC_MOUSE	Sod1	2784600	3711300	2112300	7444900	4273600	3430100	1,76
405	Phosphatidylethanolamine-binding protein 1 OS=Mus musculus GN=Pebp1 PE=1 SV=3	Other proteins	PEBP1_MOUSE	Pebp1	3388200	5712500	0	1,28E+07	1,92E+07	1,31E+07	4,97
406	Transcription cofactor vestigial-like protein 1 OS=Mus musculus GN=Vgll1 PE=1 SV=1	Other proteins	VGLL1_MOUSE	Vgll1	0	0	0	0	3,52E+07	0	>30,00
407	Glutamine--tRNA ligase OS=Mus musculus GN=Qars PE=1 SV=2	Enzymes	D3Z158_MOUSE (+1)	Qars	273160	777850	0	1996300	2105400	1103600	4,95
408	Predicted gene 20425 OS=Mus musculus GN=Gm20425 PE=4 SV=1	Other proteins	E9Q035_MOUSE (+1)	Gm20425	609690	0	1147800	2464100	1324500	1612400	3,07
409	Arginine--tRNA ligase, cytoplasmic OS=Mus musculus GN=Rars PE=1 SV=2	Enzymes	SYRC_MOUSE	Rars	394370	0	0	1459300	2835900	2035000	16,05
410	Coatomer subunit beta OS=Mus musculus GN=Copb1 PE=1 SV=1	Other proteins	COPB_MOUSE	Copb1	319170	0	216240	1919100	1894100	1029200	9,04
411	Trifunctional enzyme subunit beta, mitochondrial (Fragment) OS=Mus musculus GN=Hadhb PE=1 SV=1	Enzymes	D3YXU1_MOUSE (+1)	Hadhb	1964400	2863600	2710100	3072200	4048100	4336500	1,52
412	RAB1A, member RAS oncogene family OS=Mus musculus GN=Rab1a PE=1 SV=1	Other proteins	Q5SW87_MOUSE (+1)	Rab1a	4526200	5134900	5296100	1,61E+07	1,12E+07	9711000	2,47
413	Reticulon OS=Mus musculus GN=Rtn4 PE=1 SV=1	Other proteins	Q8BH78_MOUSE	Rtn4	1400600	5950900	901730	9497400	3161900	4392400	2,07
414	Isoform 3 of Reticulon-3 OS=Mus musculus GN=Rtn3	Other proteins	sp|Q9ES97-3|RTN3_MOUSE	Rtn3	4406900	1,26E+07	0	2,30E+07	2,69E+07	3,20E+07	4,81
415	Calnexin OS=Mus musculus GN=Canx PE=1 SV=1	Other proteins	CALX_MOUSE	Canx	0	708830	0	772240	2287200	3201400	8,83
416	Serine/threonine-protein phosphatase 2A activator OS=Mus musculus GN=Ptpa PE=1 SV=1	Enzymes	PTPA_MOUSE	Ptpa	2914800	2122100	0	5472600	1897100	3107200	2,08
417	Cytochrome P450, family 4, subfamily f, polypeptide 39 OS=Mus musculus GN=Cyp4f39 PE=1 SV=1	Other proteins	Q8BGU0_MOUSE	Cyp4f39	520580	817590	837980	2631400	2867600	1736000	3,32
418	Tripartite motif-containing protein 29 OS=Mus musculus GN=Trim29 PE=1 SV=1	Other proteins	TRI29_MOUSE	Trim29	0	1947800	1183600	749260	2138600	0	0,92
419	Eukaryotic translation initiation factor 6 OS=Mus musculus GN=Eif6 PE=1 SV=2	Translation factors	IF6_MOUSE	Eif6	1,09E+07	9574800	0	2,01E+07	8857700	1,37E+07	2,08
420	26S proteasome non-ATPase regulatory subunit 12 OS=Mus musculus GN=Psmd12 PE=1 SV=4	Proteasome	PSD12_MOUSE	Psmd12	0	0	0	1348900	1811200	2879000	>30,00
421	Translation initiation factor eIF-2B subunit alpha OS=Mus musculus GN=Eif2b1 PE=1 SV=1	Translation factors	EI2BA_MOUSE	Eif2b1	0	0	2900600	4750800	9431200	9900100	8,30
422	26S proteasome regulatory subunit 6B OS=Mus musculus GN=Psmc4 PE=1 SV=1	Proteasome	A0A140LIZ5_MOUSE (+1)	Psmc4	0	1285600	869650	5569100	7337100	7670300	9,55
423	V-type proton ATPase subunit E 1 OS=Mus musculus GN=Atp6v1e1 PE=1 SV=2	Enzymes	VATE1_MOUSE	Atp6v1e1	1801100	3931700	2869700	4858000	7841700	5083500	2,07
424	Glutathione S-transferase Mu 1 OS=Mus musculus GN=Gstm1 PE=1 SV=2	Enzymes	A2AE89_MOUSE (+1)	Gstm1	1442500	3657400	1773000	4037600	5247300	2152000	1,66
425	6-phosphogluconolactonase OS=Mus musculus GN=Pgls PE=1 SV=1	Enzymes	6PGL_MOUSE (+1)	Pgls	1970000	5869800	1938800	1951500	8474900	9737600	2,06
426	Protein/nucleic acid deglycase DJ-1 OS=Mus musculus GN=Park7 PE=1 SV=1	Enzymes	PARK7_MOUSE	Park7	2925900	1,34E+07	7313700	9363100	1,26E+07	7595700	1,25
427	Isoform 2 of Protein FAM118B OS=Mus musculus GN=Fam118b	Other proteins	sp|Q8C569-2|F118B_MOUSE (+1	Fam118b	1769500	3453300	0	0	0	4116700	0,79
429	Mitogen-activated protein kinase kinase kinase 19 (Fragment) OS=Mus musculus GN=Map3k19 PE=4 SV=2	Enzymes	A0A087WS76_MOUSE (+2)	Map3k19	415750	0	0	715220	1090300	0	4,34
430	Isocitrate dehydrogenase [NAD] subunit, mitochondrial OS=Mus musculus GN=Idh3a PE=1 SV=1	Enzymes	A0A1L1STE6_MOUSE (+1)	Idh3a	806990	2293000	940360	2852000	2745800	3666500	2,29
431	Actin-related protein 2 OS=Mus musculus GN=Actr2 PE=1 SV=1	Filaments	ARP2_MOUSE	Actr2	0	0	0	6992400	3627200	7596100	>30,00
432	Actin-related protein 2/3 complex subunit 2 OS=Mus musculus GN=Arpc2 PE=1 SV=3	Filaments	ARPC2_MOUSE (+1)	Arpc2	0	0	0	7644000	2363700	1423700	>30,00
433	ATP synthase subunit gamma OS=Mus musculus GN=Atp5c1 PE=1 SV=1	Enzymes	A2AKU9_MOUSE (+4)	Atp5c1	0	3018600	6627700	5342800	3368200	8992100	1,84
434	26S proteasome non-ATPase regulatory subunit 11 OS=Mus musculus GN=Psmd11 PE=1 SV=3	Proteasome	PSD11_MOUSE	Psmd11	1165400	0	2299200	594430	2416400	1191300	1,21

#	Identified proteins	Protein group	Accession number	Symbol	WT1	WT2	WT3	KO1	KO2	KO3	Ratio KO/WT
435	Dolichyl-diphosphooligosaccharide--protein glycosyltransferase subunit 1 OS=Mus musculus GN=Rpn1 PE=1 SV=1	Enzymes	RPN1_MOUSE	Rpn1	0	0	0	1282600	1695100	1713800	>30,00
436	2-oxoglutarate dehydrogenase, mitochondrial OS=Mus musculus GN=Ogdh PE=1 SV=1	Enzymes	Z4YJV4_MOUSE (+4)	Ogdh	368480	421640	0	881870	224870	1518700	3,32
437	Isoform 2 of Eukaryotic peptide chain release factor GTP-binding subunit ERF3A OS=Mus musculus GN=Gspt1	Translation factors	sp|Q8R050-2|ERF3A_MOUSE (+1	Gspt1	2005800	812770	0	658920	4099200	1883800	2,36
438	ADP-ribosylation factor-like protein 8B OS=Mus musculus GN=Arl8b PE=1 SV=1	Other proteins	A0A0N4SVB8_MOUSE (+3)	Arl8b	6439700	0	5697100	8147600	1,14E+07	0	1,61
439	Glutathione S-transferase omega-1 OS=Mus musculus GN=Gsto1 PE=1 SV=2	Enzymes	GSTO1_MOUSE	Gsto1	0	2030900	0	3185400	3350700	5737200	6,04
440	F-actin-capping protein subunit beta (Fragment) OS=Mus musculus GN=Capzb PE=1 SV=6	Other proteins	A0A0A0MQI9_MOUSE (+3)	Capzb	4546100	1,15E+07	7629000	2,08E+07	2,47E+07	1,62E+07	2,61
441	Obg-like ATPase 1 OS=Mus musculus GN=Ola1 PE=1 SV=1	Enzymes	B1AYJ9_MOUSE (+2)	Ola1	0	2193900	2486800	1879400	0	4688800	1,40
442	Protein transport protein Sec23B (Fragment) OS=Mus musculus GN=Sec23b PE=1 SV=1	Other proteins	A2ANA0_MOUSE (+3)	Sec23b	904980	983850	1080900	948510	1087900	1121500	1,06
443	Spermatogenesis-associated protein 5 OS=Mus musculus GN=Spata5 PE=1 SV=1	Other proteins	A0A0A0MQ80_MOUSE (+7)	Spata5	1197700	1802300	837050	2861500	4127400	3655000	2,77
444	MCG5400 OS=Mus musculus GN=Myl12a PE=1 SV=1	Other proteins	Q6ZWQ9_MOUSE	Myl12a	0	0	0	1,79E+07	1,08E+07	6271600	>30,00
445	Electron transfer flavoprotein subunit alpha, mitochondrial OS=Mus musculus GN=Etfa PE=1 SV=2	Other proteins	ETFA_MOUSE	Etfa	0	0	1171100	4955500	3430400	9295600	15,10
446	Proteasome subunit alpha type-5 OS=Mus musculus GN=Psma5 PE=1 SV=1	Proteasome	PSA5_MOUSE	Psma5	0	2151600	0	4918600	5629500	1,57E+07	12,20
447	Eukaryotic translation initiation factor 3 subunit K OS=Mus musculus GN=Eif3k PE=1 SV=1	Translation factors	sp|Q9DBZ5|EIF3K_MOUSE	Eif3k	0	0	0	1,22E+07	7661200	5150100	>30,00
448	Coatomer subunit delta OS=Mus musculus GN=Arcn1 PE=1 SV=2	Other proteins	COPD_MOUSE	Arcn1	0	0	0	1304600	1642800	983900	>30,00
449	Actin-related protein 2/3 complex subunit 1A OS=Mus musculus GN=Arpc1a PE=1 SV=1	Filaments	ARC1A_MOUSE	Arpc1a	0	1710900	807690	2437400	5128500	1724000	3,69
450	Valine--tRNA ligase (Fragment) OS=Mus musculus GN=Vars PE=1 SV=1	Enzymes	G3UY93_MOUSE (+1)	Vars	0	638220	304460	2008200	1658900	1894100	5,90
451	Ras-related protein Rab-11A OS=Mus musculus GN=Rab11a PE=4 SV=1	Other proteins	E9Q3P9_MOUSE (+4)	Rab11a	3191000	2401500	1449400	1,05E+07	6756800	2387000	2,78
452	26S proteasome non-ATPase regulatory subunit 13 OS=Mus musculus GN=Psmd13 PE=1 SV=1	Proteasome	PSD13_MOUSE	Psmd13	541440	0	627410	2160700	2269000	4246100	7,42
453	Three prime repair exonuclease 2 OS=Mus musculus GN=Trex2 PE=2 SV=1	Enzymes	TREX2_MOUSE	Trex2	1838700	7361000	4043000	1931100	2522800	5487500	0,75
454	Glutathione S-transferase Mu 5 OS=Mus musculus GN=Gstm5 PE=1 SV=1	Enzymes	E9PV63_MOUSE (+2)	Gstm5	3013200	8702000	2423600	6426300	3988000	4459800	1,05
455	60S ribosomal protein L36a OS=Mus musculus GN=Rpl36a PE=1 SV=2	Ribosome	RL36A_MOUSE	Rpl36a	5965000	2026600	5055500	0	2933400	4794600	0,59
456	Heterogeneous nuclear ribonucleoprotein L (Fragment) OS=Mus musculus GN=Hnrnpl PE=1 SV=1	Ribosome	G5E924_MOUSE (+1)	Hnrnpl	1007500	5041300	3229500	3147200	2870600	2450800	0,91
457	ATP synthase subunit f, mitochondrial OS=Mus musculus GN=Atp5j2 PE=1 SV=3	Enzymes	ATPK_MOUSE (+1)	Atp5j2	3422800	9555200	4831300	8633000	8032600	9184800	1,45
458	Isoform 2 of 60S ribosomal protein L22-like 1 OS=Mus musculus GN=Rpl22l1	Ribosome	sp|Q9D7S7-2|RL22L_MOUSE (+1)	Rpl22l1	3862000	4566200	3921600	5431900	6170300	8341800	1,61
459	Coatomer subunit gamma-1 OS=Mus musculus GN=Copg1 PE=1 SV=1	Other proteins	COPG1_MOUSE (+3)	Copg1	223000	0	0	586570	722170	1126900	10,92
460	Serine/threonine-protein kinase N3 (Fragment) OS=Mus musculus GN=Pkn3 PE=1 SV=1	Enzymes	A2BE96_MOUSE (+1)	Pkn3	1,92E+07	2,96E+07	1,83E+07	1787500	2,18E+07	0	0,35
461	Envoplakin OS=Mus musculus GN=Evpl PE=1 SV=3	Cell junctional proteins	EVPL_MOUSE	Evpl	119080	200280	786080	0	377990	0	0,34
462	Heat shock 70 kDa protein 4 OS=Mus musculus GN=Hspa4 PE=1 SV=1	Heat shock proteins	HSP74_MOUSE (+1)	Hspa4	0	0	0	0	864730	1952900	>30,00
463	Ras-related protein Rab-25 OS=Mus musculus GN=Rab25 PE=1 SV=2	Other proteins	RAB25_MOUSE	Rab25	1117600	2959200	1345900	1,35E+07	0	6569900	3,70
464	Dolichyl-diphosphooligosaccharide--protein glycosyltransferase subunit 2 OS=Mus musculus GN=Rpn2 PE=1 SV=1	Enzymes	A2ACG7_MOUSE (+1)	Rpn2	0	1588200	0	3647300	1655200	3392800	5,47
465	ATP-binding cassette sub-family E member 1 OS=Mus musculus GN=Abce1 PE=1 SV=1	Other proteins	ABCE1_MOUSE	Abce1	0	0	0	931380	2321500	4260500	>30,00
466	Importin-7 OS=Mus musculus GN=Ipo7 PE=1 SV=2	Other proteins	IPO7_MOUSE	Ipo7	0	0	1260800	400510	0	2458200	2,27
467	Synaptic vesicle membrane protein VAT-1 homolog OS=Mus musculus GN=Vat1 PE=1 SV=3	Other proteins	VAT1_MOUSE	Vat1	1206500	0	0	1933000	2601800	5068100	7,96
468	26S proteasome non-ATPase regulatory subunit 7 OS=Mus musculus GN=Psmd7 PE=1 SV=2	Proteasome	PSMD7_MOUSE	Psmd7	0	0	0	1,04E+07	9279600	8170100	>30,00
469	WD repeat-containing protein 1 OS=Mus musculus GN=Wdr1 PE=1 SV=3	Other proteins	WDR1_MOUSE	Wdr1	0	0	0	2725400	5977200	3852600	>30,00
470	Thimet oligopeptidase OS=Mus musculus GN=Thop1 PE=1 SV=1	Enzymes	A0A0R4IZY0_MOUSE (+1)	Thop1	432810	1930600	566300	0	2196700	3084300	1,80
471	Superoxide dismutase [Mn], mitochondrial OS=Mus musculus GN=Sod2 PE=1 SV=3	Enzymes	SODM_MOUSE	Sod2	0	3994100	0	5030000	5377900	9272800	4,93
472	Isoform 2 of Poly(rC)-binding protein 2 OS=Mus musculus GN=Pcbp2	Other proteins	sp|Q61990-2|PCBP2_MOUSE (+2	Pcbp2	4206800	5638400	4378800	8750900	7825600	6865300	1,65
473	Heterogeneous nuclear ribonucleoprotein H OS=Mus musculus GN=Hnrnph1 PE=1 SV=3	Other proteins	HNRH1_MOUSE (+1)	Hnrnph1	1728800	1866100	1939300	3071000	924350	0	0,72
474	Exportin-1 OS=Mus musculus GN=Xpo1 PE=1 SV=1	Other proteins	XPO1_MOUSE	Xpo1	246740	0	202550	615210	632770	863310	4,70
475	Proteasome subunit alpha type-1 OS=Mus musculus GN=Psma1 PE=1 SV=1	Proteasome	PSA1_MOUSE	Psma1	0	2200900	0	2544700	6200000	4617900	6,07
476	Arachidonate 12-lipoxygenase, 12R-type OS=Mus musculus GN=Alox12b PE=1 SV=1	Enzymes	LX12B_MOUSE	Alox12b	1568300	1259700	0	783670	1335500	2757100	1,72
477	60S ribosomal protein L29 OS=Mus musculus GN=Gm17669 PE=3 SV=1	Ribosome	F6QL70_MOUSE (+2)	Rpl29	1,42E+07	0	1,06E+07	3,08E+07	1,92E+07	0	2,02
478	Actin-related protein 2/3 complex subunit 3 OS=Mus musculus GN=Arpc3 PE=1 SV=3	Filaments	ARPC3_MOUSE (+1)	Arpc3	0	0	0	3798600	3242500	1707000	>30,00
479	Microtubule-associated proteins 1A/1B light chain 3B OS=Mus musculus GN=Map1lc3b PE=1 SV=3	Filaments	MLP3B_MOUSE	Map1lc3b	0	0	0	4,16E+07	4,00E+07	3,52E+07	>30,00
480	Costars family protein ABRACL (Fragment) OS=Mus musculus GN=Abracl PE=1 SV=1	Other proteins	A0A1W2P7X0_MOUSE (+2)	Abracl	0	0	6768900	1,36E+07	1,23E+07	2,36E+07	7,30
481	Ubiquitin carboxyl-terminal hydrolase 29 (Fragment) OS=Mus musculus GN=Usp29 PE=2 SV=1	Enzymes	Q8CAH4_MOUSE (+1)	Usp29	5974200	0	7324600	8659800	1,07E+07	0	1,46
482	Protein disulfide-isomerase A6 OS=Mus musculus GN=Pdia6 PE=1 SV=3	Enzymes	PDIA6_MOUSE (+1)	Pdia6	0	0	0	4250500	1544600	6819000	>30,00
483	Sulfotransferase OS=Mus musculus GN=Sult2b1 PE=1 SV=1	Enzymes	A0A1B0GR49_MOUSE (+3)	Sult2b1	0	2476000	0	1770900	3020700	5861900	4,30
484	Proteasome subunit alpha type-7 OS=Mus musculus GN=Psma7 PE=1 SV=1	Proteasome	PSA7_MOUSE	Psma7	0	0	0	4038300	5704600	2743000	>30,00
485	Proteasome subunit beta type-6 OS=Mus musculus GN=Psmb6 PE=1 SV=3	Proteasome	PSB6_MOUSE	Psmb6	0	0	0	1,02E+07	3991400	1,55E+07	>30,00
486	ATP-dependent 6-phosphofructokinase OS=Mus musculus GN=Pfkp PE=1 SV=1	Enzymes	Q8C605_MOUSE (+2)	Pfkp	0	0	0	1004800	1016700	653590	>30,00
487	Cysteine protease OS=Mus musculus GN=Atg4b PE=1 SV=1	Enzymes	A0A0R4J065_MOUSE (+1)	Atg4b	326750	0	0	600990	2060300	1315000	12,17
488	Transforming protein RhoA (Fragment) OS=Mus musculus GN=Rhoa PE=1 SV=1	Other proteins	A0A0A6YXF6_MOUSE (+1)	Rhoa	0	0	4809700	2569900	0	3071900	1,17
489	Hydroperoxide isomerase ALOXE3 OS=Mus musculus GN=Aloxe3 PE=1 SV=2	Enzymes	LOXE3_MOUSE	Aloxe3	1492700	1138700	2157900	748630	682730	980390	0,50
490	Protein phosphatase methylesterase 1 OS=Mus musculus GN=Ppme1 PE=1 SV=5	Enzymes	PPME1_MOUSE	Ppme1	552690	0	0	2430100	2626200	1514000	11,89
491	DnaJ homolog subfamily A member 2 OS=Mus musculus GN=Dnaja2 PE=1 SV=1	Other proteins	DNJA2_MOUSE	Dnaja2	0	0	743210	0	646400	1087400	2,33
492	ATP-binding cassette sub-family F member 1 OS=Mus musculus GN=Abcf1 PE=1 SV=1	Other proteins	ABCF1_MOUSE	Abcf1	0	0	282620	1317100	626190	534890	8,77
493	Probable ATP-dependent RNA helicase DDX6 OS=Mus musculus GN=Ddx6 PE=1 SV=1	Enzymes	DDX6_MOUSE	Ddx6	652240	2752200	841010	952060	1269600	2670200	1,15
495	Apoptosis-associated speck-like protein containing a CARD OS=Mus musculus GN=Pycard PE=1 SV=1	Other proteins	ASC_MOUSE	Pycard	2075700	0	1602200	2384800	3186500	4145900	2,64
496	Collagen alpha-1(VI) chain OS=Mus musculus GN=Col6a1 PE=1 SV=1	Other proteins	CO6A1_MOUSE	Col6a1	311930	2061200	382560	437250	1012200	636940	0,76
497	Mitochondrial fission 1 protein OS=Mus musculus GN=Fis1 PE=1 SV=1	Other proteins	FIS1_MOUSE	Fis1	2400300	4156700	2609700	3993700	6362500	5709900	1,75
498	Microtubule-associated proteins 1A/1B light chain 3A OS=Mus musculus GN=Map1lc3a PE=1 SV=1	Filaments	MLP3A_MOUSE	Map1lc3a	0	0	0	3,11E+07	2,84E+07	3,17E+07	>30,00
499	Insulin-degrading enzyme (Fragment) OS=Mus musculus GN=Ide PE=1 SV=1	Other proteins	F6RPJ9_MOUSE (+1)	Ide	0	1217500	0	331240	0	3514800	3,16
500	Proteasome subunit alpha type-4 OS=Mus musculus GN=Psma4 PE=1 SV=1	Proteasome	PSA4_MOUSE	Psma4	0	0	2201100	4373400	8957200	3090200	7,46
501	Testis-expressed gene 264 OS=Mus musculus GN=Tex264 PE=1 SV=1	Other proteins	E9Q137_MOUSE	Tex264	0	0	0	2814200	7789900	0	>30,00
502	Target of Myb protein 1 OS=Mus musculus GN=Tom1 PE=1 SV=1	Other proteins	Q3UDC3_MOUSE (+1)	Tom1	0	0	0	551390	5051200	0	>30,00
503	Splicing factor, proline- and glutamine-rich OS=Mus musculus GN=Sfpq PE=1 SV=1	Other proteins	SFPQ_MOUSE	Sfpq	1420700	2166600	1623600	2433400	1373200	0	0,73
504	Protein unc-45 homolog A OS=Mus musculus GN=Unc45a PE=1 SV=2	Other proteins	UN45A_MOUSE	Unc45a	0	568370	1210300	562890	759010	1601400	1,64
505	Eukaryotic translation initiation factor 4E OS=Mus musculus GN=Eif4e PE=1 SV=1	Translation factors	A0A0G2JFB4_MOUSE (+3)	Eif4e	0	0	6773100	0	3532400	1,88E+07	3,29
506	Alpha-1-antitrypsin 1-1 OS=Mus musculus GN=Serpina1a PE=1 SV=1	Other proteins	A0A0A0MQA3_MOUSE (+3)	Serpina1a	947560	2090800	0	4322900	0	3045100	2,42
507	Adenylate kinase isoenzyme 1 (Fragment) OS=Mus musculus GN=Ak1 PE=1 SV=1	Enzymes	Z4YN97_MOUSE (+2)	Ak1	4226500	0	0	1,37E+07	0	7790200	5,09
508	Annexin (Fragment) OS=Mus musculus GN=Anxa4 PE=1 SV=3	Other proteins	A0A0N4SW89_MOUSE (+1)	Anxa4	0	0	1410100	5561900	4136200	1243600	7,76
509	Translation initiation factor eIF-2B subunit epsilon OS=Mus musculus GN=Eif2b5 PE=1 SV=1	Translation factors	EI2BE_MOUSE	Eif2b5	629510	2288700	0	1351000	1588200	2954600	2,02
511	Exportin-2 OS=Mus musculus GN=Cse1l PE=1 SV=1	Other proteins	E9Q1T9_MOUSE (+1)	Cse1l	4,60E+07	6,37E+07	4,56E+07	4,12E+07	0	5,38E+07	0,61
512	Kinesin-like protein (Fragment) OS=Mus musculus GN=Kif5b PE=1 SV=1	Enzymes	E9QAK5_MOUSE (+1)	Kif5b	0	0	0	0	563970	2206300	>30,00
513	Serpin B13 OS=Mus musculus GN=Serpinb13 PE=3 SV=1	Other proteins	D6RHN5_MOUSE (+1)	Serpinb13	2341800	1,29E+07	3749200	6607100	8021600	1,07E+07	1,33
514	Atlastin-3 OS=Mus musculus GN=Atl3 PE=1 SV=1	Other proteins	E9PYT3_MOUSE (+1)	Atl3	0	0	0	2179900	1336400	2221000	>30,00
515	Cytoskeleton-associated protein 4 OS=Mus musculus GN=Ckap4 PE=1 SV=2	Other proteins	CKAP4_MOUSE	Ckap4	0	0	0	798240	0	638120	>30,00
516	Cytochrome b-c1 complex subunit 2, mitochondrial OS=Mus musculus GN=Uqcrc2 PE=1 SV=1	Other proteins	QCR2_MOUSE	Uqcrc2	1492300	2058100	1636700	1633700	1666600	4101600	1,43
517	CD9 antigen OS=Mus musculus GN=Cd9 PE=1 SV=2	Other proteins	CD9_MOUSE	Cd9	3368100	0	6292300	0	0	0	0,00
518	Importin subunit alpha-3 OS=Mus musculus GN=Kpna4 PE=1 SV=1	Other proteins	A0A0B4J1E7_MOUSE (+1)	Kpna4	795190	1589900	0	2356600	2016400	3322000	3,23
519	MCG10748, isoform CRA_b OS=Mus musculus GN=Rap1a PE=1 SV=1	Other proteins	A0A0G2JDL9_MOUSE (+3)	Rap1a	1635500	2809400	2833100	2693300	2856100	0	0,76
520	Eukaryotic translation initiation factor 1 OS=Mus musculus GN=Eif1 PE=1 SV=2	Translation factors	EIF1_MOUSE	Eif1	2549800	0	0	4196800	5746200	8489600	7,23
521	Eukaryotic translation initiation factor 1A, X-chromosomal OS=Mus musculus GN=Eif1ax PE=2 SV=3	Translation factors	IF1AX_MOUSE (+1)	Eif1ax	0	1854900	0	2296100	2886400	3131500	4,48
522	FAS-associated factor 2 (Fragment) OS=Mus musculus GN=Faf2 PE=1 SV=1	Other proteins	D3YUP4_MOUSE (+2)	Faf2	2847100	4596900	3567100	5891900	8971600	8164600	2,09
523	Multiple inositol polyphosphate phosphatase 1 OS=Mus musculus GN=Minpp1 PE=1 SV=3	Enzymes	MINP1_MOUSE	Minpp1	0	0	0	1536300	1931500	1871600	>30,00
524	Protein SET (Fragment) OS=Mus musculus GN=Set PE=1 SV=1	Other proteins	A2BE92_MOUSE (+3)	Set	1,44E+07	0	2491900	1,04E+07	1,27E+07	0	1,37
525	Aspartate--tRNA ligase, cytoplasmic OS=Mus musculus GN=Dars PE=1 SV=2	Enzymes	SYDC_MOUSE	Dars	0	0	0	469700	1517000	1577000	>30,00
526	Isoform 2 of Heterogeneous nuclear ribonucleoprotein U OS=Mus musculus GN=Hnrnpu	Other proteins	sp|Q8VEK3-2|HNRPU_MOUSE (+	Hnrnpu	0	865170	1076600	582630	0	2841100	1,76
527	Peptidyl-tRNA hydrolase 2, mitochondrial OS=Mus musculus GN=Ptrh2 PE=1 SV=1	Enzymes	PTH2_MOUSE	Ptrh2	1775800	5041500	0	0	1465400	1,24E+07	2,03
528	Microtubule-actin cross-linking factor 1 OS=Mus musculus GN=Macf1 PE=1 SV=1	Filaments	A0A0A0MQA6_MOUSE (+11)	Macf1	0	0	47423	77413	94252	92356	5,57
529	2-amino-3-ketobutyrate coenzyme A ligase, mitochondrial OS=Mus musculus GN=Gcat PE=1 SV=2	Enzymes	KBL_MOUSE	Gcat	0	0	1001000	2433600	0	4758700	7,19
530	Isoform 2 of Neutral alpha-glucosidase AB OS=Mus musculus GN=Ganab	Enzymes	sp|Q8BHN3-2|GANAB_MOUSE (+	Ganab	0	0	0	0	246510	0	>30,00
531	Toll-interacting protein OS=Mus musculus GN=Tollip PE=1 SV=1	Other proteins	Q8C5G6_MOUSE (+1)	Tollip	1400200	0	0	3794400	2520100	0	4,51
532	F-actin-capping protein subunit alpha-2 (Fragment) OS=Mus musculus GN=Capza2 PE=1 SV=1	Filaments	A0A0N4SVM0_MOUSE (+2)	Capza2	0	0	0	2665300	3205200	3511800	>30,00
533	Vimentin OS=Mus musculus GN=Vim PE=1 SV=1	Other proteins	A0A0A6YWC8_MOUSE (+1)	Vim	0	1,22E+08	1,80E+08	0	1,08E+08	1,13E+08	0,73
534	Ubiquitin-conjugating enzyme E2 D3 (Fragment) OS=Mus musculus GN=Ube2d3 PE=1 SV=1	Enzymes	A0A0G2JE32_MOUSE (+3)	Ube2d3	0	1,82E+07	1,33E+07	1,38E+07	1,65E+07	0	0,96
535	Transmembrane emp24 domain-containing protein 10 OS=Mus musculus GN=Tmed10 PE=1 SV=1	Other proteins	A0A1Y7VM54_MOUSE (+1)	Tmed10	0	0	0	4887400	5828500	8132500	>30,00
536	Heterogeneous nuclear ribonucleoprotein M OS=Mus musculus GN=Hnrnpm PE=1 SV=1	Other proteins	B8JK32_MOUSE (+3)	Hnrnpm	0	890160	279130	643940	0	780610	1,22
537	Dual specificity phosphatase 21 OS=Mus musculus GN=Dusp21 PE=1 SV=1	Enzymes	DUS21_MOUSE	Dusp21	0	1,37E+07	0	0	0	1,17E+07	0,85
538	CAD protein OS=Mus musculus GN=Cad PE=1 SV=1	Other proteins	E9QAI5_MOUSE (+4)	Cad	74991	186790	129160	272280	345970	334560	2,44
539	rRNA 2'-O-methyltransferase fibrillarin OS=Mus musculus GN=Fbl PE=1 SV=2	Enzymes	FBRL_MOUSE	Fbl	2766400	1899900	2616600	3652300	3201100	1984600	1,21
540	Microsomal glutathione S-transferase 3 OS=Mus musculus GN=Mgst3 PE=1 SV=1	Enzymes	MGST3_MOUSE	Mgst3	2404400	0	0	0	4423600	4761600	3,82
541	Cytochrome c oxidase subunit 6B1 OS=Mus musculus GN=Cox6b1 PE=1 SV=1	Enzymes	A0A140LIU3_MOUSE (+1)	Cox6b1	4312700	0	0	7633300	1,29E+07	1,43E+07	8,09
542	Ras-related protein Rab-27B OS=Mus musculus GN=Rab27b PE=1 SV=3	Other proteins	RB27B_MOUSE	Rab27b	0	1276800	0	2665500	2351600	3268100	6,49
543	Nucleophosmin OS=Mus musculus GN=Npm1 PE=1 SV=1	Other proteins	NPM_MOUSE (+3)	Npm1	1306200	1738500	5075600	0	0	0	0,00
544	26S proteasome regulatory subunit 8 OS=Mus musculus GN=Psmc5 PE=1 SV=1	Proteasome	PRS8_MOUSE (+1)	Psmc5	0	0	0	0	3162300	2603200	>30,00
545	Acetyl-CoA acetyltransferase, mitochondrial OS=Mus musculus GN=Acat1 PE=1 SV=1	Enzymes	THIL_MOUSE	Acat1	0	0	0	0	1,03E+07	6486500	>30,00
546	Inverted formin-2 OS=Mus musculus GN=Inf2 PE=1 SV=1	Other proteins	E9QLA5_MOUSE (+2)	Inf2	0	0	0	363380	437990	0	>30,00
547	Dipeptidyl peptidase 3 OS=Mus musculus GN=Dpp3 PE=1 SV=2	Enzymes	DPP3_MOUSE	Dpp3	0	0	0	1060600	0	643230	>30,00
548	Eukaryotic translation initiation factor 2 subunit 2 OS=Mus musculus GN=Eif2s2 PE=1 SV=1	Translation factors	IF2B_MOUSE	Eif2s2	0	0	0	1646100	3351100	2061600	>30,00
549	26S proteasome regulatory subunit 7 OS=Mus musculus GN=Psmc2 PE=1 SV=5	Proteasome	PRS7_MOUSE (+1)	Psmc2	0	0	0	2121000	1972800	918510	>30,00
550	Isoform 2 of General vesicular transport factor p115 OS=Mus musculus GN=Uso1	Other proteins	sp|Q9Z1Z0-2|USO1_MOUSE (+2)	Uso1	0	931700	951940	789620	0	0	0,42
551	Asparagine--tRNA ligase, cytoplasmic OS=Mus musculus GN=Nars PE=1 SV=2	Enzymes	SYNC_MOUSE	Nars	0	0	0	454390	585290	2522200	>30,00
552	AHNAK nucleoprotein 2 (Fragment) OS=Mus musculus GN=Ahnak2 PE=1 SV=1	Other proteins	F7DBB3_MOUSE	Ahnak2	0	0	0	354390	916380	1311600	>30,00
553	Collagen alpha-2(I) chain OS=Mus musculus GN=Col1a2 PE=1 SV=2	Other proteins	CO1A2_MOUSE	Col1a2	128990	0	422330	215580	0	0	0,39
554	Lymphocyte antigen 6D OS=Mus musculus GN=Ly6d PE=1 SV=1	Other proteins	LY6D_MOUSE	Ly6d	0	9985300	0	9874000	0	1,10E+07	2,09
555	Ubiquitin thioesterase OTU1 OS=Mus musculus GN=Yod1 PE=1 SV=1	Enzymes	OTU1_MOUSE	Yod1	0	1418100	0	2515000	3043600	0	3,92
556	Dermokine (Fragment) OS=Mus musculus GN=Dmkn PE=1 SV=1	Other proteins	A0A087WQH3_MOUSE (+7)	Dmkn	0	1480000	0	2329300	1435500	0	2,54
557	Cytoplasmic dynein 1 intermediate chain 2 OS=Mus musculus GN=Dync1i2 PE=1 SV=1	Other proteins	A2BFF5_MOUSE (+4)	Dync1i2	1007800	0	0	825060	570510	2810900	4,17
558	UDP-glucose:glycoprotein glucosyltransferase 1 OS=Mus musculus GN=Uggt1 PE=1 SV=4	Enzymes	UGGG1_MOUSE	Uggt1	0	0	0	906570	1242700	0	>30,00
559	Ubiquitin thioesterase OTUB1 OS=Mus musculus GN=Otub1 PE=1 SV=1	Enzymes	D3YWF6_MOUSE (+1)	Otub1	0	0	1224500	3477900	2304200	2097800	6,44
560	Hypoxia up-regulated protein 1 OS=Mus musculus GN=Hyou1 PE=1 SV=1	Other proteins	HYOU1_MOUSE	Hyou1	0	0	0	0	657020	791500	>30,00
561	V-set and immunoglobulin domain-containing protein 10-like OS=Mus musculus GN=Vsig10l PE=4 SV=1	Other proteins	A0A0N4SUI1_MOUSE (+1)	Vsig10l	0	0	0	0	926020	0	>30,00
562	ATP synthase subunit d, mitochondrial OS=Mus musculus GN=Atp5h PE=1 SV=3	Enzymes	ATP5H_MOUSE (+1)	Atp5h	0	0	0	4936100	2802700	7090900	>30,00
563	Ubiquitin-conjugating enzyme E2 variant 2 OS=Mus musculus GN=Ube2v2 PE=1 SV=1	Enzymes	A6X925_MOUSE (+5)	Ube2v2	0	0	0	0	1950000	0	>30,00
564	10 kDa heat shock protein, mitochondrial OS=Mus musculus GN=Hspe1 PE=1 SV=2	Heat shock proteins	CH10_MOUSE	Hspe1	0	8884400	0	1644600	0	1,20E+07	1,53
565	Uncharacterized protein OS=Mus musculus PE=4 SV=1	Other proteins	A0A1W2P6Q8_MOUSE (+5)	Cdc5l	1162700	0	0	2683800	2046900	2571000	6,28
566	ADP-ribosylation factor-like protein 1 OS=Mus musculus GN=Arl1 PE=1 SV=1	Other proteins	ARL1_MOUSE	Arl1	0	0	2140200	1910300	9515300	0	5,34
567	Prostaglandin E synthase 3 OS=Mus musculus GN=Ptges3 PE=1 SV=1	Enzymes	D3Z7C6_MOUSE (+1)	Ptges3	0	0	0	5125800	6333800	5810400	>30,00
568	Small nuclear ribonucleoprotein E OS=Mus musculus GN=Snrpe PE=1 SV=1	Other proteins	E9Q0W8_MOUSE (+2)	Snrpe	6114400	1,32E+07	7041900	0	0	0	0,00
569	Transmembrane emp24 domain-containing protein 2 (Fragment) OS=Mus musculus GN=Tmed2 PE=1 SV=1	Other proteins	F6V6T4_MOUSE (+2)	Tmed2	1731600	0	0	1864500	0	0	1,08
570	Serine/threonine-protein phosphatase 2A catalytic subunit alpha isoform OS=Mus musculus GN=Ppp2ca PE=1 SV=1	Enzymes	PP2AA_MOUSE (+1)	Ppp2ca	0	3244000	0	1957000	0	4261100	1,92
571	Barrier-to-autointegration factor OS=Mus musculus GN=Banf1 PE=1 SV=1	Other proteins	BAF_MOUSE	Banf1	2069200	5099200	3041100	3955800	4709900	0	0,85
572	Staphylococcal nuclease domain-containing protein 1 OS=Mus musculus GN=Snd1 PE=1 SV=1	Other proteins	SND1_MOUSE	Snd1	438090	0	491210	588660	0	1232000	1,96
573	Ig kappa chain V-V region HP 123E6 OS=Mus musculus PE=1 SV=1	Other proteins	KV5AD_MOUSE	KV5AD	4,84E+07	0	0	3,32E+07	3,24E+07	0	1,35
574	Alpha-2-HS-glycoprotein OS=Mus musculus GN=Ahsg PE=1 SV=1	Other proteins	FETUA_MOUSE	Ahsg	0	0	1839000	3020900	4386400	3410800	5,88
575	Cystatin-C OS=Mus musculus GN=Cst3 PE=1 SV=2	Other proteins	CYTC_MOUSE	Cst3	0	6831800	3522500	5370500	5091800	0	1,01
577	Complement component 1 Q subcomponent-binding protein, mitochondrial OS=Mus musculus GN=C1qbp PE=1 SV=1	Other proteins	C1QBP_MOUSE (+1)	C1qbp	0	0	0	0	9504200	1,24E+07	>30,00
578	eIF-2-alpha kinase activator GCN1 OS=Mus musculus GN=Gcn1 PE=1 SV=1	Other proteins	GCN1_MOUSE	Gcn1	221030	0	0	0	161240	0	0,73
579	26S proteasome non-ATPase regulatory subunit 8 OS=Mus musculus GN=Psmd8 PE=1 SV=2	Proteasome	PSMD8_MOUSE (+2)	Psmd8	0	0	0	881630	0	5686200	>30,00
580	Basic leucine zipper and W2 domain-containing protein 1 OS=Mus musculus GN=Bzw1 PE=1 SV=1	Other proteins	A0A087WQS2_MOUSE (+1)	Bzw1	0	0	0	0	4004500	0	>30,00
581	GRIP and coiled-coil domain containing 2 OS=Mus musculus GN=Gcc2 PE=1 SV=1	Other proteins	B2RSU7_MOUSE (+3)	Gcc2	0	211340	152210	0	0	0	0,00
582	Basic leucine zipper and W2 domain-containing protein 2 OS=Mus musculus GN=Bzw2 PE=1 SV=1	Other proteins	BZW2_MOUSE	Bzw2	0	0	0	0	1622900	0	>30,00
583	Proteasome subunit beta type-3 OS=Mus musculus GN=Psmb3 PE=1 SV=1	Proteasome	PSB3_MOUSE	Psmb3	0	0	0	8140200	4974800	1191400	>30,00
584	1-acylglycerol-3-phosphate O-acyltransferase ABHD5 OS=Mus musculus GN=Abhd5 PE=1 SV=1	Enzymes	sp|Q9DBL9|ABHD5_MOUSE	Abhd5	0	0	0	4157600	2622500	4056700	>30,00
585	Spectrin beta chain OS=Mus musculus GN=Sptbn2 PE=1 SV=1	Other proteins	Q68FG2_MOUSE	Sptbn2	0	418020	0	108540	157710	0	0,64
586	UBX domain-containing protein 1 OS=Mus musculus GN=Ubxn1 PE=1 SV=1	Other proteins	UBXN1_MOUSE	Ubxn1	0	0	0	2734700	1608200	1122400	>30,00
587	Nap1l4 protein OS=Mus musculus GN=Nap1l4 PE=1 SV=1	Other proteins	B7ZNL2_MOUSE (+1)	Nap1l4	0	6333800	0	1738200	2091200	0	0,60
588	Nucleolar protein 58 OS=Mus musculus GN=Nop58 PE=1 SV=1	Other proteins	A0A0A0MQ76_MOUSE (+1)	Nop58	1175700	0	0	2,39E+07	0	0	20,36
589	Very-long-chain 3-oxoacyl-CoA reductase OS=Mus musculus GN=Hsd17b12 PE=1 SV=1	Enzymes	sp|O70503|DHB12_MOUSE	Hsd17b12	0	0	0	4079400	1529400	0	>30,00
590	Proteasome subunit beta type-5 OS=Mus musculus GN=Psmb5 PE=1 SV=3	Proteasome	PSB5_MOUSE	Psmb5	0	0	0	2336800	0	2944600	>30,00
591	Dolichyl-diphosphooligosaccharide--protein glycosyltransferase 48 kDa subunit OS=Mus musculus GN=Ddost PE=1 SV=2	Enzymes	OST48_MOUSE	Ddost	1309600	0	0	5570100	978030	0	5,00
592	N-alpha-acetyltransferase 15, NatA auxiliary subunit OS=Mus musculus GN=Naa15 PE=1 SV=1	Enzymes	A0A0A6YW80_MOUSE (+2)	Naa15	231180	773760	0	0	1165300	0	1,16
593	NHP2-like protein 1 OS=Mus musculus GN=Snu13 PE=1 SV=4	Other proteins	NH2L1_MOUSE	Snu13	5390900	1,57E+07	0	0	1,06E+07	0	0,50
594	Disks large-associated protein 4 OS=Mus musculus GN=Dlgap4 PE=1 SV=1	Other proteins	B7ZNS2_MOUSE (+7)	Dlgap4	606410	986130	315150	0	0	1126000	0,59
595	3-ketoacyl-CoA thiolase A, peroxisomal (Fragment) OS=Mus musculus GN=Acaa1a PE=1 SV=1	Enzymes	H3BJC1_MOUSE (+4)	Acaa1a	0	1,20E+07	3979400	8980600	1,02E+07	0	1,21
596	Prefoldin subunit 3 OS=Mus musculus GN=Vbp1 PE=1 SV=2	Other proteins	PFD3_MOUSE (+1)	Vbp1	0	2006900	0	1999600	2407800	0	2,20
597	Isoform 2 of Unconventional myosin-Ic OS=Mus musculus GN=Myo1c	Filaments	sp|Q9WTI7-2|MYO1C_MOUSE (+	Myo1c	0	0	243240	340870	214470	0	2,28
598	Arginase-1 OS=Mus musculus GN=Arg1 PE=1 SV=1	Enzymes	ARGI1_MOUSE	Arg1	0	0	0	1485900	0	2174800	>30,00
599	MCG6846, isoform CRA_c OS=Mus musculus GN=Prpsap1 PE=1 SV=1	Other proteins	B1AT82_MOUSE (+1)	Prpsap1	485340	0	889650	0	1230100	1358200	1,88
600	Autophagy-related protein 9B OS=Mus musculus GN=Atg9b PE=2 SV=2	Other proteins	ATG9B_MOUSE	Atg9b	518990	1183700	464780	782270	0	0	0,36
602	Glutathione S-transferase A1 OS=Mus musculus GN=Gsta1 PE=1 SV=2	Enzymes	GSTA1_MOUSE (+1)	Gsta1	0	2767700	0	1327500	1467600	2099500	1,77

#	Identified proteins	Protein group	Accession number	Symbol	WT1	WT2	WT3	KO1	KO2	KO3	Ratio KO/WT
603	Major vault protein OS=Mus musculus GN=Mvp PE=1 SV=1	Other proteins	E9Q3X0_MOUSE (+1)	Mvp	0	485330	295440	0	702450	978010	2,15
604	Sec24-related gene family, member C (S. cerevisiae) (Fragment) OS=Mus musculus GN=Sec24c PE=4 SV=1	Other proteins	A0A286YD08_MOUSE (+2)	Sec24c	0	4134200	2444800	0	3221800	0	0,49
605	Small nuclear ribonucleoprotein Sm D1 OS=Mus musculus GN=Snrpd1 PE=1 SV=1	Other proteins	SMD1_MOUSE	Snrpd1	0	0	0	0	0	1,14E+07	>30,00
606	E3 ubiquitin-protein ligase HUWE1 OS=Mus musculus GN=Huwe1 PE=1 SV=1	Enzymes	A2AFQ0_MOUSE (+4)	Huwe1	0	0	0	0	87421	69579	>30,00
607	Alcohol dehydrogenase 6B (class V) OS=Mus musculus GN=Adh6b PE=3 SV=1	Enzymes	A0A1D5RMB0_MOUSE	Adh6b	0	4545300	0	0	0	634780	0,14
608	Catenin alpha-1 OS=Mus musculus GN=Ctnna1 PE=1 SV=1	Other proteins	CTNA1_MOUSE	Ctnna1	0	2535600	0	0	1613600	0	0,64
609	Filaggrin OS=Mus musculus GN=Flg PE=1 SV=1	Other proteins	A0A0A6YXI5_MOUSE	Flg	3082300	9603100	0	0	5952500	1,24E+07	1,45
610	MCG123521, isoform CRA_a OS=Mus musculus GN=Mettl7a3 PE=4 SV=1	Other proteins	G3X9G9_MOUSE	Mettl7a3	0	0	0	4260900	0	3460000	>30,00
611	Calcium-binding and coiled-coil domain-containing protein 1 OS=Mus musculus GN=Calcoco1 PE=1 SV=2	Other proteins	CACO1_MOUSE (+1)	Calcoco1	0	0	0	988430	0	1271600	>30,00
612	DCC-interacting protein 13-beta (Fragment) OS=Mus musculus GN=Appl2 PE=1 SV=2	Other proteins	D3Z340_MOUSE (+1)	Appl2	0	5166500	0	0	6022300	0	1,17
613	Lactoylglutathione lyase OS=Mus musculus GN=Glo1 PE=1 SV=3	Enzymes	LGUL_MOUSE	Glo1	0	0	0	2008200	0	5209400	>30,00
614	Polypyrimidine tract-binding protein 3 OS=Mus musculus GN=Ptbp3 PE=1 SV=1	Other proteins	G3UXA6_MOUSE (+3)	Ptbp3	957010	0	0	4200900	9067800	0	13,86
615	Annexin A5 OS=Mus musculus GN=Anxa5 PE=1 SV=1	Other proteins	ANXA5_MOUSE	Anxa5	0	0	0	0	0	1547800	>30,00
616	RAS-related C3 botulinum substrate 3, isoform CRA_a (Fragment) OS=Mus musculus GN=Rac3 PE=1 SV=1	Other proteins	A2AC13_MOUSE (+3)	Rac3	0	0	0	2978700	2838200	0	>30,00
617	Lysine--tRNA ligase OS=Mus musculus GN=Kars PE=1 SV=1	Enzymes	Q8R2P8_MOUSE (+1)	Kars	0	0	0	546550	1906500	0	>30,00
618	Proteasome subunit beta type-1 OS=Mus musculus GN=Psmb1 PE=1 SV=1	Proteasome	PSB1_MOUSE	Psmb1	0	0	0	3035600	0	3555200	>30,00
619	Probable ATP-dependent RNA helicase DDX5 OS=Mus musculus GN=Ddx5 PE=1 SV=2	Enzymes	DDX5_MOUSE (+2)	Ddx5	0	0	1411500	2048700	1863300	0	2,77
620	Elongation of very long chain fatty acids protein 1 OS=Mus musculus GN=Elovl1 PE=1 SV=1	Other proteins	ELOV1_MOUSE	Elovl1	0	0	0	5661400	6305700	9890600	>30,00
621	Ly6/PLAUR domain-containing protein 3 OS=Mus musculus GN=Lypd3 PE=2 SV=1	Other proteins	LYPD3_MOUSE	Lypd3	0	5243600	2768000	0	0	3993200	0,50
622	Mimecan OS=Mus musculus GN=Ogn PE=1 SV=1	Other proteins	MIME_MOUSE	Ogn	0	4502700	0	0	1548400	0	0,34
623	phospholipase A2 inhibitor and Ly6/PLAUR domain-containing protein OS=Mus musculus GN=Pinlyp PE=2 SV=1	Enzymes	PINLY_MOUSE	Pinlyp	0	3461000	0	3081300	0	0	0,89
624	40S ribosomal protein S29 OS=Mus musculus GN=Rps29 PE=3 SV=2	Ribosome	RS29_MOUSE	Rps29	0	0	0	1,28E+07	1,32E+07	0	>30,00
625	T-complex protein 11-like protein 2 OS=Mus musculus GN=Tcp11l2 PE=1 SV=1	Other proteins	T11L2_MOUSE	Tcp11l2	0	610990	0	863100	541190	0	2,30
626	Proteasome subunit alpha type-3 OS=Mus musculus GN=Psma3 PE=1 SV=3	Proteasome	PSA3_MOUSE	Psma3	0	0	0	2351300	2013200	1690200	>30,00
627	Glutathione S-transferase (Fragment) OS=Mus musculus GN=Gsta3 PE=1 SV=1	Enzymes	A0A087WQI6_MOUSE (+1)	Gsta3	0	0	0	5447900	0	2,30E+07	>30,00
628	Transmembrane emp24 domain-containing protein 9 OS=Mus musculus GN=Tmed9 PE=4 SV=1	Other proteins	A0A286YDS5_MOUSE (+1)	Tmed9	0	0	0	0	876860	4580000	>30,00
629	Argininosuccinate synthase OS=Mus musculus GN=Ass1 PE=1 SV=1	Enzymes	ASSY_MOUSE	Ass1	0	0	0	0	0	2646900	>30,00
630	Inorganic pyrophosphatase OS=Mus musculus GN=Ppa1 PE=1 SV=1	Enzymes	IPYR_MOUSE	Ppa1	0	0	581630	0	0	2524400	4,34
631	Nuclear pore complex protein Nup160 OS=Mus musculus GN=Nup160 PE=1 SV=2	Other proteins	NU160_MOUSE	Nup160	0	574520	0	0	190670	460680	1,13
632	Ubiquitin-conjugating enzyme E2 L3 OS=Mus musculus GN=Ube2l3 PE=1 SV=1	Enzymes	UB2L3_MOUSE	Ube2l3	0	0	0	4530800	4926300	6264500	>30,00
633	Glyoxalase domain-containing protein 4 OS=Mus musculus GN=Glod4 PE=1 SV=1	Other proteins	E9Q197_MOUSE (+4)	Glod4	0	0	0	1353700	1700400	1795900	>30,00
634	Breast cancer type 2 susceptibility protein homolog (Fragment) OS=Mus musculus GN=Brca2 PE=1 SV=1	Other proteins	A0A0J9YVI7_MOUSE (+1)	Brca2	3969300	6251300	0	0	0	0	0,00
635	TAR DNA-binding protein 43 (Fragment) OS=Mus musculus GN=Tardbp PE=1 SV=1	Other proteins	A0A087WQA5_MOUSE (+8)	Tardbp	1557500	0	0	0	1690900	0	1,09
636	Ras-related protein Ral-A OS=Mus musculus GN=Rala PE=1 SV=1	Other proteins	RALA_MOUSE (+1)	Rala	0	4732500	2633200	0	0	0	0,00
637	Protein S100-A10 OS=Mus musculus GN=S100a10 PE=1 SV=2	Other proteins	S10AA_MOUSE	S100a10	0	5988100	0	0	0	1,03E+07	1,71
638	NADH-cytochrome b5 reductase OS=Mus musculus GN=Cyb5r3 PE=1 SV=1	Enzymes	F2Z456_MOUSE (+2)	Cyb5r3	0	0	0	1650500	1526000	1718300	>30,00
639	NADH dehydrogenase [ubiquinone] 1 alpha subcomplex subunit 10, mitochondrial (Fragment) OS=Mus musculus GN=N	Enzymes	A0A087WR38_MOUSE (+1)	Ndufa10	0	0	0	3178200	0	8647000	>30,00
640	MCG1051009 OS=Mus musculus GN=Serpina3k PE=1 SV=1	Other proteins	A0A0R4J0I1_MOUSE	Serpina3k	622990	0	0	1661500	0	1760300	5,49
641	Protein S100-A9 OS=Mus musculus GN=S100a9 PE=1 SV=3	Other proteins	S10A9_MOUSE	S100a9	0	0	0	5428200	0	0	>30,00
643	Exocyst complex component 1 OS=Mus musculus GN=Exoc1 PE=1 SV=4	Other proteins	EXOC1_MOUSE (+2)	Exoc1	0	301330	0	273390	0	444340	2,38
644	WW domain-binding protein 2 OS=Mus musculus GN=Wbp2 PE=1 SV=1	Other proteins	WBP2_MOUSE	Wbp2	0	1183800	0	0	0	1218800	1,03
645	Pre-mRNA-processing-splicing factor 8 OS=Mus musculus GN=Prpf8 PE=1 SV=2	Other proteins	PRP8_MOUSE	Prpf8	0	261990	0	0	328810	256720	2,23
646	Protein FAM98A OS=Mus musculus GN=Fam98a PE=1 SV=1	Other proteins	FA98A_MOUSE	Fam98a	440610	697200	0	0	0	1233900	1,08
647	Isoform 2 of Disheveled-associated activator of morphogenesis 1 OS=Mus musculus GN=Daam1	Other proteins	sp|Q8BPM0-2|DAAM1_MOUSE (	Daam1	122440	370440	0	0	0	245580	0,50
648	EF-hand domain-containing protein D1 OS=Mus musculus GN=Efhd1 PE=1 SV=1	Other proteins	EFHD1_MOUSE	Efhd1	0	0	0	9787500	0	0	>30,00
650	Serine/threonine-protein phosphatase 2A 55 kDa regulatory subunit B alpha isoform OS=Mus musculus GN=Ppp2r2a PE	Enzymes	2ABA_MOUSE (+6)	Ppp2r2a	0	0	1067300	0	0	1399100	1,31
652	Retinol dehydrogenase 12 OS=Mus musculus GN=Rdh12 PE=1 SV=1	Enzymes	A0A0R4J1M3_MOUSE (+1)	Rdh12	0	0	0	0	1882600	1433500	>30,00
653	Proline-rich protein 9 OS=Mus musculus GN=Prr9 PE=4 SV=1	Other proteins	PRR9_MOUSE	Prr9	0	0	0	0	9105900	1,66E+07	>30,00
654	MCG21235 OS=Mus musculus GN=Serpinb3b PE=2 SV=1	Other proteins	Q9D1Q5_MOUSE	Serpinb3b	0	1542400	0	0	0	1386300	0,90
655	ATP-citrate synthase OS=Mus musculus GN=Acly PE=1 SV=1	Enzymes	ACLY_MOUSE (+1)	Acly	0	0	410990	0	0	409610	1,00
656	Ectopic P granules protein 5 homolog OS=Mus musculus GN=Epg5 PE=1 SV=2	Other proteins	EPG5_MOUSE	Epg5	0	0	0	0	0	325550	>30,00
657	Isoform Short of Serine/arginine-rich splicing factor 3 OS=Mus musculus GN=Srsf3	Other proteins	sp|P84104-2|SRSF3_MOUSE (+1)	Srsf3	0	0	0	0	0	4622500	>30,00
658	Keratinocyte differentiation-associated protein OS=Mus musculus GN=Krtdap PE=1 SV=1	Other proteins	A0A087WRR2_MOUSE (+3)	Krtdap	0	4,60E+07	2,41E+07	0	0	0	0,00
659	Cingulin-like 1 OS=Mus musculus GN=Cgnl1 PE=1 SV=1	Other proteins	B2RSU6_MOUSE (+4)	Cgnl1	823730	0	0	0	0	0	0,00
660	MCG22610 OS=Mus musculus GN=Rnf39 PE=2 SV=1	Other proteins	A2RT81_MOUSE	Rnf39	0	0	0	0	634340	1831800	>30,00
661	Leukotriene A-4 hydrolase OS=Mus musculus GN=Lta4h PE=1 SV=4	Enzymes	LKHA4_MOUSE	Lta4h	0	0	0	0	1321900	0	>30,00
662	Enhancer of rudimentary homolog OS=Mus musculus GN=Erh PE=1 SV=1	Other proteins	ERH_MOUSE	Erh	0	6504600	0	0	0	0	0,00
664	Cytochrome b5 type B OS=Mus musculus GN=Cyb5b PE=1 SV=1	Other proteins	CYB5B_MOUSE	Cyb5b	0	0	0	6068300	0	8279300	>30,00
665	Cytochrome b-c1 complex subunit 1, mitochondrial OS=Mus musculus GN=Uqcrc1 PE=1 SV=2	Other proteins	QCR1_MOUSE	Uqcrc1	0	0	0	2995500	0	0	>30,00
666	Phospholipase A-2-activating protein OS=Mus musculus GN=Plaa PE=1 SV=4	Enzymes	PLAP_MOUSE	Plaa	0	0	0	0	644250	733160	>30,00
667	Bcl-2-modifying factor OS=Mus musculus GN=Bmf PE=4 SV=1	Other proteins	A2AV74_MOUSE (+2)	Bmf	0	0	9,12E+07	0	1,12E+08	0	1,23
668	Dynamin-2 OS=Mus musculus GN=Dnm2 PE=1 SV=2	Other proteins	F8WIV5_MOUSE (+4)	Dnm2	0	0	0	256630	675280	0	>30,00
670	NADH-ubiquinone oxidoreductase 75 kDa subunit, mitochondrial OS=Mus musculus GN=Ndufs1 PE=1 SV=2	Enzymes	NDUS1_MOUSE	Ndufs1	0	0	789100	0	1164000	0	1,48
671	Serpin B12 OS=Mus musculus GN=Serpinb12 PE=2 SV=1	Other proteins	SPB12_MOUSE	Serpinb12	0	1863100	0	1193400	0	0	0,64
672	Aminoacyl tRNA synthase complex-interacting multifunctional protein 2 OS=Mus musculus GN=Aimp2 PE=1 SV=2	Enzymes	AIMP2_MOUSE (+1)	Aimp2	0	0	0	0	2648600	1885300	>30,00
673	Ras-related protein Rab-2A OS=Mus musculus GN=Rab2a PE=1 SV=1	Other proteins	RAB2A_MOUSE	Rab2a	0	1387600	0	840590	0	0	0,61
674	High mobility group protein B1 OS=Mus musculus GN=Hmgb1 PE=1 SV=1	Other proteins	A0A0J9YUD8_MOUSE (+2)	Hmgb1	0	3277100	0	0	5051700	0	1,54
675	Splicing factor 1 (Fragment) OS=Mus musculus GN=Sf1 PE=1 SV=1	Other proteins	D3YVH4_MOUSE (+6)	Sf1	0	0	0	3047600	0	0	>30,00
677	Zinc finger, C3H1-type-containing OS=Mus musculus GN=Zfc3h1 PE=1 SV=1	Other proteins	B2RT41_MOUSE	Zfc3h1	0	0	0	0	0	3,59E+07	>30,00
678	Vesicle-trafficking protein SEC22b OS=Mus musculus GN=Sec22b PE=1 SV=1	Other proteins	E9Q6R3_MOUSE (+1)	Sec22b	0	0	0	2594100	0	0	>30,00
679	Acetyl-CoA acetyltransferase, cytosolic OS=Mus musculus GN=Acat2 PE=1 SV=1	Other proteins	G3XA25_MOUSE (+1)	Acat2	0	0	0	1365100	2957600	0	>30,00
680	Lipopolysaccharide-responsive and beige-like anchor protein OS=Mus musculus GN=Lrba PE=1 SV=1	Other proteins	A0A0A6YXL6_MOUSE (+3)	Lrba	0	0	0	185520	0	0	>30,00
681	Cytoplasmic FMR1-interacting protein 1 OS=Mus musculus GN=Cyfip1 PE=1 SV=1	Other proteins	A0A0R4J119_MOUSE (+5)	Cyfip1	192640	0	0	0	0	0	0,00
683	Proteasome activator complex subunit 1 (Fragment) OS=Mus musculus GN=Psme1 PE=1 SV=1	Proteasome	G3UWN9_MOUSE (+3)	Psme1	0	0	0	0	2020000	0	>30,00
684	Peroxisomal carnitine O-octanoyltransferase OS=Mus musculus GN=Crot PE=1 SV=1	Enzymes	OCTC_MOUSE	Crot	473730	0	0	0	0	0	0,00
688	Signal recognition particle subunit SRP68 OS=Mus musculus GN=Srp68 PE=1 SV=1	Other proteins	A2AAN2_MOUSE (+1)	Srp68	0	0	0	0	0	1173600	>30,00
689	Mitochondrial-processing peptidase subunit alpha OS=Mus musculus GN=Pmpca PE=1 SV=1	Enzymes	A2AIW9_MOUSE (+1)	Pmpca	0	0	0	0	0	765070	>30,00
690	Transmembrane protein 62 OS=Mus musculus GN=Tmem62 PE=1 SV=1	Other proteins	A2AQ56_MOUSE (+1)	Tmem62	0	0	0	0	0	2020800	>30,00
691	Transcription factor BTF3 homolog 4 OS=Mus musculus GN=Btf3l4 PE=1 SV=1	Other proteins	BT3L4_MOUSE	Btf3l4	0	0	0	0	0	1,07E+07	>30,00
692	START domain-containing protein 10 OS=Mus musculus GN=Stard10 PE=1 SV=1	Other proteins	E9PVP0_MOUSE (+5)	Stard10	0	0	0	0	0	1598900	>30,00
693	Fructose-1,6-bisphosphatase isozyme 2 OS=Mus musculus GN=Fbp2 PE=1 SV=2	Enzymes	F16P2_MOUSE	Fbp2	0	0	0	0	0	2907600	>30,00
694	Spermatogenesis-associated protein 31 OS=Mus musculus GN=Spata31 PE=1 SV=1	Other proteins	SPT31_MOUSE	Spata31	364050	0	0	0	0	0	0,00
695	Sodium/potassium-transporting ATPase subunit alpha-1 OS=Mus musculus GN=Atp1a1 PE=1 SV=1	Enzymes	AT1A1_MOUSE	Atp1a1	0	0	1006300	0	0	0	0,00
696	Aquaporin-3 OS=Mus musculus GN=Aqp3 PE=1 SV=1	Other proteins	AQP3_MOUSE	Aqp3	0	0	6903900	0	0	0	0,00
697	Epidermal growth factor receptor kinase substrate 8-like protein 1 (Fragment) OS=Mus musculus GN=Eps8l1 PE=1 SV=1	Enzymes	E9PWX4_MOUSE (+2)	Eps8l1	0	0	0	0	0	4250500	>30,00
698	Collagen alpha-1(VII) chain OS=Mus musculus GN=Col7a1 PE=1 SV=3	Other proteins	CO7A1_MOUSE	Col7a1	0	0	0	0	0	229050	>30,00
699	Carbonyl reductase [NADPH] 3 OS=Mus musculus GN=Cbr3 PE=1 SV=1	Enzymes	CBR3_MOUSE	Cbr3	0	0	0	0	0	1830000	>30,00
700	Trk-fused OS=Mus musculus GN=Tfg PE=1 SV=1	Other proteins	B8JJG6_MOUSE (+4)	Tfg	0	0	0	0	2168500	0	>30,00
701	CAAX prenyl protease 1 homolog OS=Mus musculus GN=Zmpste24 PE=1 SV=2	Enzymes	FACE1_MOUSE	Zmpste24	0	0	0	0	4275600	0	>30,00
702	Septin-7 OS=Mus musculus GN=Sept7 PE=1 SV=2	Other proteins	E9Q1G8_MOUSE (+2)	Sept7	0	0	939900	0	0	0	0,00
703	Gigaxonin (Fragment) OS=Mus musculus GN=Gan PE=1 SV=1	Other proteins	F6TZU3_MOUSE (+1)	Gan	576320	0	0	0	0	0	0,00
704	Transmembrane protein 43 OS=Mus musculus GN=Tmem43 PE=1 SV=1	Other proteins	TMM43_MOUSE	Tmem43	0	0	0	0	0	3570700	>30,00
705	Isoform 2 of Protein AATF OS=Mus musculus GN=Aatf	Other proteins	sp|Q9JKX4-2|AATF_MOUSE (+2)	Aatf	1679900	0	0	0	0	0	0,00
706	Prenylcysteine oxidase (Fragment) OS=Mus musculus GN=Pcyox1 PE=1 SV=7	Enzymes	D3Z275_MOUSE (+2)	Pcyox1	0	0	0	0	2600400	0	>30,00
707	RIKEN cDNA 9230117E20 OS=Mus musculus GN=Spink12 PE=4 SV=1	Other proteins	A0A0R4J109_MOUSE (+1)	Spink12	0	0	0	5448600	0	0	>30,00
708	Cytoplasmic dynein 1 light intermediate chain 1 OS=Mus musculus GN=Dync1li1 PE=1 SV=1	Other proteins	DC1L1_MOUSE	Dync1li1	0	0	0	0	1391900	0	>30,00
709	Adenylosuccinate lyase OS=Mus musculus GN=Adsl PE=1 SV=1	Enzymes	E9Q242_MOUSE (+2)	Adsl	0	0	0	0	0	730210	>30,00
711	Translation initiation factor eIF-2B subunit beta OS=Mus musculus GN=Eif2b2 PE=1 SV=1	Translation factors	EI2BB_MOUSE	Eif2b2	0	0	0	0	0	2446300	>30,00
712	Protein canopy homolog 2 OS=Mus musculus GN=Cnpy2 PE=1 SV=1	Other proteins	CNPY2_MOUSE	Cnpy2	0	0	0	0	2930600	0	>30,00
713	Isoform 2 of Non-POU domain-containing octamer-binding protein OS=Mus musculus GN=Nono	Other proteins	sp|Q99K48-2|NONO_MOUSE (+1	Nono	0	1489200	0	8113300	0	0	5,45
714	Isoform 2 of Acetyl-CoA carboxylase 1 OS=Mus musculus GN=Acaca	Enzymes	sp|Q5SWU9-2|ACACA_MOUSE (+	Acaca	0	0	0	216120	0	0	>30,00
715	Asporin OS=Mus musculus GN=Aspn PE=1 SV=1	Other proteins	ASPN_MOUSE	Aspn	0	1646600	0	0	0	0	0,00
716	26S proteasome non-ATPase regulatory subunit 14 OS=Mus musculus GN=Psmd14 PE=1 SV=2	Proteasome	PSDE_MOUSE	Psmd14	0	0	0	0	3439700	0	>30,00
717	Carbonyl reductase [NADPH] 2 OS=Mus musculus GN=Cbr2 PE=1 SV=1	Enzymes	CBR2_MOUSE (+1)	Cbr2	0	0	0	1964500	0	0	>30,00
718	Importin 4, isoform CRA_a OS=Mus musculus GN=Ipo4 PE=1 SV=1	Other proteins	D6RES6_MOUSE (+2)	Ipo4	0	0	0	1713200	0	0	>30,00
719	GTP-binding protein 1 OS=Mus musculus GN=Gtpbp1 PE=1 SV=2	Other proteins	GTPB1_MOUSE	Gtpbp1	0	0	0	0	816340	0	>30,00
720	Histidine--tRNA ligase, cytoplasmic OS=Mus musculus GN=Hars PE=1 SV=2	Enzymes	SYHC_MOUSE	Hars	0	0	0	0	770260	0	>30,00
721	V-type proton ATPase subunit H OS=Mus musculus GN=Atp6v1h PE=1 SV=1	Enzymes	A0A0A6YWP6_MOUSE (+2)	Atp6v1h	0	0	0	1639900	0	0	>30,00

Notes:

Search Engine: MaxQuant

Version: 1.5.7.4
Fragment Tolerance: 20 PPM (Monoisotopic)
Fixed Modifications: +57 on C (Carbamidomethyl)
Variable Modifications: +1 on NQ (Deamidated), +16 on M (Oxidation), +42 on n (Acetyl)
Digestion Enzyme: stricttrypsin
Max Missed Cleavages: 2

Scaffold: Version: Scaffold_4.8.4

Protein Grouping Strategy: Experiment-wide grouping with protein cluster analysis
Peptide Thresholds: 0.0% minimum
Protein Thresholds: 20.0% minimum and 1 peptide minimum
Peptide FDR: 0.4% (Decoy)
Protein FDR: 1.4% (Decoy)
